# The 2023 wearable photoplethysmography roadmap

**DOI:** 10.1088/1361-6579/acead2

**Published:** 2023-11-29

**Authors:** Peter H Charlton, John Allen, Raquel Bailón, Stephanie Baker, Joachim A Behar, Fei Chen, Gari D Clifford, David A Clifton, Harry J Davies, Cheng Ding, Xiaorong Ding, Jessilyn Dunn, Mohamed Elgendi, Munia Ferdoushi, Daniel Franklin, Eduardo Gil, Md Farhad Hassan, Jussi Hernesniemi, Xiao Hu, Nan Ji, Yasser Khan, Spyridon Kontaxis, Ilkka Korhonen, Panicos A Kyriacou, Pablo Laguna, Jesús Lázaro, Chungkeun Lee, Jeremy Levy, Yumin Li, Chengyu Liu, Jing Liu, Lei Lu, Danilo P Mandic, Vaidotas Marozas, Elisa Mejía-Mejía, Ramakrishna Mukkamala, Meir Nitzan, Tania Pereira, Carmen C Y Poon, Jessica C Ramella-Roman, Harri Saarinen, Md Mobashir Hasan Shandhi, Hangsik Shin, Gerard Stansby, Toshiyo Tamura, Antti Vehkaoja, Will Ke Wang, Yuan-Ting Zhang, Ni Zhao, Dingchang Zheng, Tingting Zhu

**Affiliations:** 1 Department of Public Health and Primary Care, University of Cambridge, Cambridge, CB1 8RN, United Kingdom; 2 Research Centre for Biomedical Engineering, City, University of London, London, EC1V 0HB, United Kingdom; 3 Research Centre for Intelligent Healthcare, Coventry University, Coventry, CV1 5RW, United Kingdom; 4 Faculty of Medical Sciences, Newcastle University, Newcastle upon Tyne, NE2 4HH, United Kingdom; 5 Biomedical Signal Interpretation and Computational Simulation (BSICoS) Group, Aragon Institute of Engineering Research (I3A), IIS Aragon, University of Zaragoza, E-50018 Zaragoza, Spain; 6 CIBER-BBN, Instituto de Salud Carlos III, C/Monforte de Lemos 3-5, E-28029 Madrid, Spain; 7 College of Science and Engineering, James Cook University, Cairns, 4878 Queensland, Australia; 8 Faculty of Biomedical Engineering, Technion Israel Institute of Technology, Haifa, 3200003, Israel; 9 Department of Electrical and Electronic Engineering, Southern University of Science and Technology, Shenzhen, 518055 Guandong, People’s Republic of China; 10 Department of Biomedical Informatics, Emory University, Atlanta, GA 30322, United States of America; 11 Coulter Department of Biomedical Engineering, Georgia Institute of Technology, Atlanta, GA 30332, United States of America; 12 Department of Engineering Science, University of Oxford, Oxford, OX3 7DQ, United Kingdom; 13 Department of Electrical and Electronic Engineering, Imperial College London, London, SW7 2AZ, United Kingdom; 14 Department of Biomedical Engineering, Georgia Institute of Technology, Atlanta, GA 30332, United States of America; 15 Department of Biomedical Engineering, Emory University, Atlanta, GA 30322, United States of America; 16 School of Life Science and Technology, University of Electronic Science and Technology of China, Chengdu, 611731, People’s Republic of China; 17 Department of Biomedical Engineering, Duke University, Durham, NC 27708-0187, United States of America; 18 Department of Biostatistics & Bioinformatics, Duke University, Durham, NC 27708-0187, United States of America; 19 Duke Clinical Research Institute, Durham, NC 27705-3976, United States of America; 20 Biomedical and Mobile Health Technology Laboratory, Department of Health Sciences and Technology, ETH Zurich, Zurich, 8008, Switzerland; 21 Department of Electrical and Computer Engineering, University of Southern California, 90089, Los Angeles, California, United States of America; 22 The Institute for Technology and Medical Systems (ITEMS), Keck School of Medicine, University of Southern California, Los Angeles, CA 90033, United States of America; 23 Institute of Biomedical Engineering, Translational Biology & Engineering Program, Ted Rogers Centre for Heart Research, University of Toronto, Toronto, M5G 1M1, Canada; 24 Finnish Cardiovascular Research Center Tampere, Faculty of Medicine and Health Technology, Tampere University, Tampere, 33720, Finland; 25 Tampere Heart Hospital, Wellbeing Services County of Pirkanmaa, Tampere, 33520, Finland; 26 Nell Hodgson Woodruff School of Nursing, Emory University, Atlanta, 30322, Georgia, United States of America; 27 Department of Biomedical Informatics, School of Medicine, Emory University, Atlanta, 30322, Georgia, United States of America; 28 Department of Computer Sciences, College of Arts and Sciences, Emory University, Atlanta, GA 30322, United States of America; 29 Hong Kong Center for Cerebrocardiovascular Health Engineering (COCHE), Hong Kong Science and Technology Park, Hong Kong, 999077, People’s Republic of China; 30 Digital Health Devices Division, Medical Device Evaluation Department, National Institute of Food and Drug Safety Evaluation, Ministry of Food and Drug Safety, Cheongju, 28159, Republic of Korea; 31 Faculty of Electrical and Computer Engineering, Technion Institute of Technology, Haifa, 3200003, Israel; 32 State Key Laboratory of Bioelectronics, School of Instrument Science and Engineering, Southeast University, Nanjing 210096, People’s Republic of China; 33 Analog Devices Inc, San Jose, CA 95124, United States of America; 34 Department of Electronics Engineering, Kaunas University of Technology, 44249 Kaunas, Lithuania; 35 Biomedical Engineering Institute, Kaunas University of Technology, 44249 Kaunas, Lithuania; 36 Department of Bioengineering and Department of Anesthesiology and Perioperative Medicine, University of Pittsburgh, Pittsburgh, Pennsylvania, United States of America; 37 Department of Physics/Electro-Optic Engineering, Lev Academic Center, 91160 Jerusalem, Israel; 38 INESC TEC—Institute for Systems and Computer Engineering, Technology and Science, Porto, 4200-465, Portugal; 39 Faculty of Engineering, University of Porto, Porto, 4200-465, Portugal; 40 GMed IT, Hong Kong, People’s Republic of China; 41 Department of Biomedical Engineering and Herbert Wertheim College of Medicine, Florida International University, Miami, FL 33174, United States of America; 42 Department of Digital Medicine, Asan Medical Center, University of Ulsan College of Medicine, Seoul, 05505, Republic of Korea; 43 Northern Vascular Centre, Freeman Hospital, Newcastle upon Tyne, NE7 7DN, United Kingdom; 44 Future Robotics Organization, Waseda University, Tokyo, 1698050, Japan; 45 PulseOn Ltd, Espoo, 02150, Finland; 46 Department of Biomedical Engineering, City University of Hong Kong, Hong Kong, 999077, People’s Republic of China; 47 Department of Electronic Engineering, The Chinese University of Hong Kong, Hong Kong

**Keywords:** blood pressure, cardiovascular, fitness, physiological monitoring, sensor, signal processing, smartwatch

## Abstract

Photoplethysmography is a key sensing technology which is used in wearable devices such as smartwatches and fitness trackers. Currently, photoplethysmography sensors are used to monitor physiological parameters including heart rate and heart rhythm, and to track activities like sleep and exercise. Yet, wearable photoplethysmography has potential to provide much more information on health and wellbeing, which could inform clinical decision making. This Roadmap outlines directions for research and development to realise the full potential of wearable photoplethysmography. Experts discuss key topics within the areas of sensor design, signal processing, clinical applications, and research directions. Their perspectives provide valuable guidance to researchers developing wearable photoplethysmography technology.

## 1. Introduction

### Peter H Charlton

Department of Public Health and Primary Care, University of Cambridge, Cambridge, United Kingdom Research Centre for Biomedical Engineering, City, University of London, London, United Kingdom

The widespread use of wearable devices provides opportunity to monitor health unobtrusively and at scale in daily life. Wearables such as smartwatches and fitness trackers commonly use the optical sensing technology ‘photoplethysmography’ to acquire an arterial pulse wave signal, from which a wealth of physiological information can be derived. Several promising applications of wearable photoplethysmography are either being translated into clinical practice or in development, including detecting abnormal heart rhythms, monitoring blood pressure, and identifying sleep disorders. There is great potential for wearable photoplethysmography to improve health and wellbeing, meaning much further work is warranted to realise its full benefits.

The field of wearable photoplethysmography is a highly attractive area for research and development. Photoplethysmography entered clinical use in the 1980s in the form of pulse oximeters, revolutionising the measurement of arterial blood oxygen saturation, and pulse oximeters remain widely used in many clinical settings. In the 2010s, photoplethysmography was incorporated into consumer wearables such as smartwatches and fitness trackers, which are now used by millions each day. The technology is simple enough to be widely understood: an optical sensor for measuring the blood pulse. And yet it is complex enough to capture the imagination of researchers worldwide: there is no consensus yet on the physiological origins of the photoplethysmogram signal (section 24). The potential benefits have been the subject of high-impact publications: studies of multiple wearable photoplethysmography devices have found them to be a useful tool for detecting atrial fibrillation and prompting potentially life-saving treatments (Guo *et al* 2019, Perez *et al* 2019, Lubitz *et al* 2022). It is deemed important enough for a researcher in the field to have been nominated for a Nobel Prize (Miyasaka *et al* 2021). Wearable photoplethysmography is convenient enough to facilitate unobtrusive health monitoring in millions of individuals (Natarajan *et al* 2020, Radin *et al* 2020). Nonetheless, ‘Convenience alone is insufficient. The approach has to work’ (section 21). In other words, to realise the full benefits of wearable photoplethysmography, the technology should be developed to provide extensive physiological information which can be reliably used in clinical decision making.

### Overview

**Figure 1.**
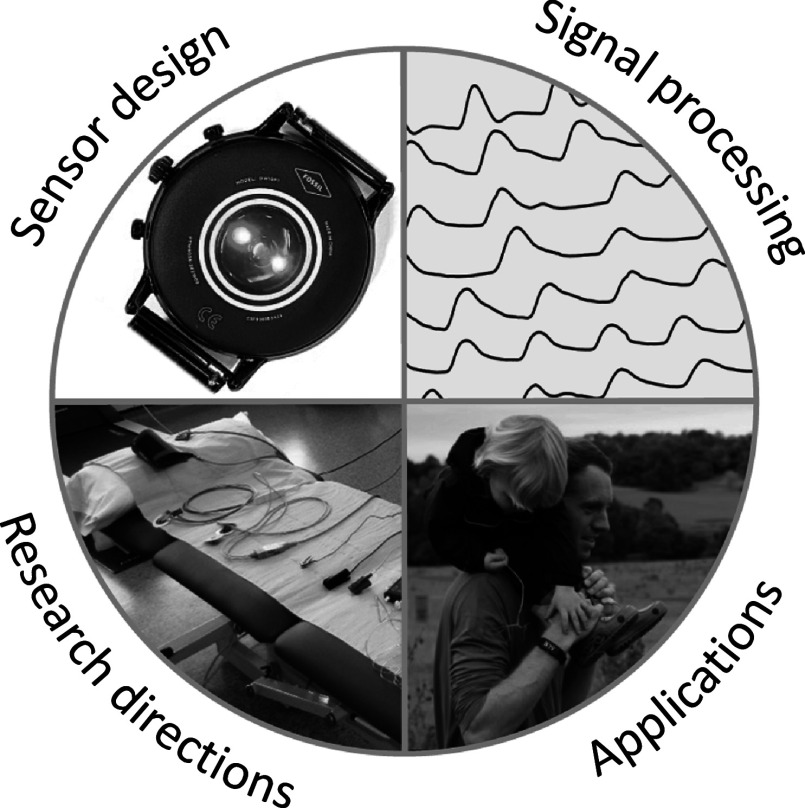
The four areas of wearable photoplethysmography covered in this Roadmap. Sources: the ‘sensor design’ panel is adapted from: Marozas and Charlton, ‘Wearable photoplethysmography devices’, Zenodo, 2021, https://doi.org/10.5281/zenodo.4601548 (*CC BY 4.0*). The ‘Research directions’ panel is adapted from: Charlton, ‘Presentation of: An assessment of algorithms to estimate respiratory rate from the electrocardiogram and photoplethysmogram’, Zenodo, 2016, https://doi.org/10.5281/zenodo.6402455 (*CC BY 4.0*).

In this Roadmap, experts provide their perspectives on the future development of wearable photoplethysmography. The Roadmap is intended to help guide future research and development in the field. The Roadmap covers a range of key topics within the field of wearable photoplethysmography. The section on each topic provides a concise overview of the topic’s current status, the key challenges ahead, and the advances in science and technology which could address these challenges. The sections stand alone, so can be read individually. Topics lie within four areas, as illustrated in figure 1:

1. **Sensor design**: innovative approaches to photoplethysmography sensor design are discussed, including flexible sensors, in-ear sensors (‘hearables’), and multi-wavelength photoplethysmography.
2. **Signal processing**: advances in photoplethysmography signal processing for physiological monitoring are presented, including developments in blood pressure, respiratory and sleep monitoring. These are considered alongside strategies to handle low quality signals and motion artifact.
3. **Applications**: clinical and consumer applications of wearable photoplethysmography are discussed, ranging from established applications such as the detection of atrial fibrillation, to emerging applications such as mental health assessment.
4. **Research directions**: key directions for future research are highlighted, including developing and validating techniques for blood pressure monitoring, and investigating sources of inaccuracy in wearable photoplethysmography measurements.

### Themes

Several key themes emerge from the Roadmap which could inform overall strategies for research and development:

• Expanding functionality: much research is focused on expanding the functionality of wearable photoplethysmography devices, such as developing and validating techniques to monitor novel parameters such as blood pressure, oxygen saturation, and even detailed respiratory metrics such as inhalation and exhalation times. The expanding functionality of devices will enable detailed physiological assessments to be performed unobtrusively in daily life, which otherwise could only be performed in clinical settings.
• Optimising sensor design: the design of photoplethysmography sensors strongly affects both the quality of the acquired signals, and the physiological information contained within them. Key design decisions include: the anatomical site of the sensor (noting that the wrist is not ideal for many applications), the mode of sensor attachment, and the wavelength(s) of light to be used. These decisions can all influence PPG pulse wave morphology and resulting measurements. Decisions are often a compromise between making wearable photoplethysmography devices as unobtrusive and as accurate as possible.
• Approaches to signal processing: a range of approaches have been proposed to process photoplethysmography signals, and it is not yet clear which provide the best performance, nor whether different approaches are best suited to different applications. Approaches include traditional pulse wave analysis, and more recently pulse wave decomposition and deep learning.
• Identifying potential applications: new potential applications for wearable photoplethysmography will emerge as the functionality of the technology expands. In particular, the ability to reliably monitor blood pressure and oxygen saturation would open new opportunities. There is potential for data-driven health interventions and services, as well as applications in resource-constrained settings.
• Gaining trust: the successful use of wearable photoplethysmography devices to improve health and well-being will require the trust of multiple stakeholders including clinicians, policy makers, and most importantly device users. Important approaches to gain trust include ensuring that applications are supported by a strong evidence-base, and that personal data are handled appropriately.

### Challenges and solutions

Several key challenges and potential solutions emerge from the Roadmap:

• Signal quality: the photoplethysmogram signal is highly susceptible to noise caused by motion artifact and other sources. Different approaches have been proposed to identify and handle low quality signals, ranging from optimising sensor design, to developing signal processing techniques to delineate low quality periods and recover noise-free signals.
• Signal processing resources: the development of signal processing algorithms is greatly aided by open datasets, and open code for existing algorithms. The acquisition of datasets in daily life, from patients with diseases of interest, can be facilitated by devices which provide the raw photoplethysmogram signal.
• Validating devices: processes for validating devices should be carefully designed to provide a comprehensive understanding of real-world performance across subjects with different characteristics. Work has begun on creating standards and recommended protocols to guide robust validation studies.
• Sources of inaccuracy: studies have identified several potential sources of inaccuracy of wearable photoplethysmography devices. Work is ongoing to identify further sources, and to mitigate against them.
• Equity: when developing wearable photoplethysmography devices, it is important to be mindful of the equity of access to devices and their performance. Key considerations are the cost of devices, and the performance of devices in subjects with different characteristics (most notably, subjects with different skin types).
• Establishing best practices: it may be beneficial to establish best practices in wearable photoplethysmography across several areas, including: sensor design; signal quality assessment; signal processing algorithms; benchmark datasets; measurement protocols; and standardising evaluation protocols.

### Outlook

Wearable photoplethysmography is a valuable tool for unobtrusive physiological monitoring. Despite having entered the consumer market only a decade ago, it is already being used to guide clinical decision making in selected applications. There is potential for wearable photoplethysmography to be used for many more health and wellbeing applications. High quality, patient-centred research and development is required to realise this potential for the benefit of society.

### Further reading

This Roadmap provides a concise overview of several topics in the field of wearable photoplethysmography, focusing on directions for future work. For further information on selected topics, please see the references in table 1.

**Table 1.** Further reading on wearable photoplethysmography.

Topic	References
Wearable photoplethysmography: an overview	Charlton *et al* (2022)
Skin-compatible wearable photoplethysmography sensors	Lee *et al* (2021b)
Hearables	Masè *et al* (2020)
Multiwavelength photoplethysmography	Ray *et al* (2023)
Pulse rate variability	Mejia-Mejia *et al* (2020)
Respiratory rate	Charlton *et al* (2018)
Wearable pulse oximetry	Tamura (2019)
Signal quality assessment	Desquins *et al* (2022)
Motion artifact	Park *et al* (2022b)
Applications	Loh *et al* (2022)
Atrial fibrillation detection	Pereira *et al* (2020)
Pulse oximetry biomarkers	Levy *et al* (2021b)
Mental health assessment	Hickey *et al* (2021)
Cuffless blood pressure	Mukkamala *et al* (2022b)
Vascular age assessment	Charlton *et al* (2022)
Sources of inaccuracy	Fine *et al* (2021b)
Alternative sensing technologies	Meng *et al* (2022)
Establishing best practices	Charlton *et al* (2022)

## Acknowledgments

This work was supported by the British Heart Foundation under Grant FS/20/20/34626, and COST Action CA18216 VascAgeNet, supported by COST (European Cooperation in Science and Technology, www.cost.eu). The authors have confirmed that any identifiable participants in this study have given their consent for publication.

## SENSOR DESIGN

## 2. Photoplethysmography with emerging materials and sensors

### Munia Ferdoushi^1,2^, Md Farhad Hassan^1,2^ and Yasser Khan^1,2,*^

^1^Department of Electrical and Computer Engineering, University of Southern California, Los Angeles, CA 90089

^2^The Institute for Technology and Medical Systems (ITEMS), Keck School of Medicine, University of Southern California, Los Angeles, CA 90033

### Status

**Figure 2.**
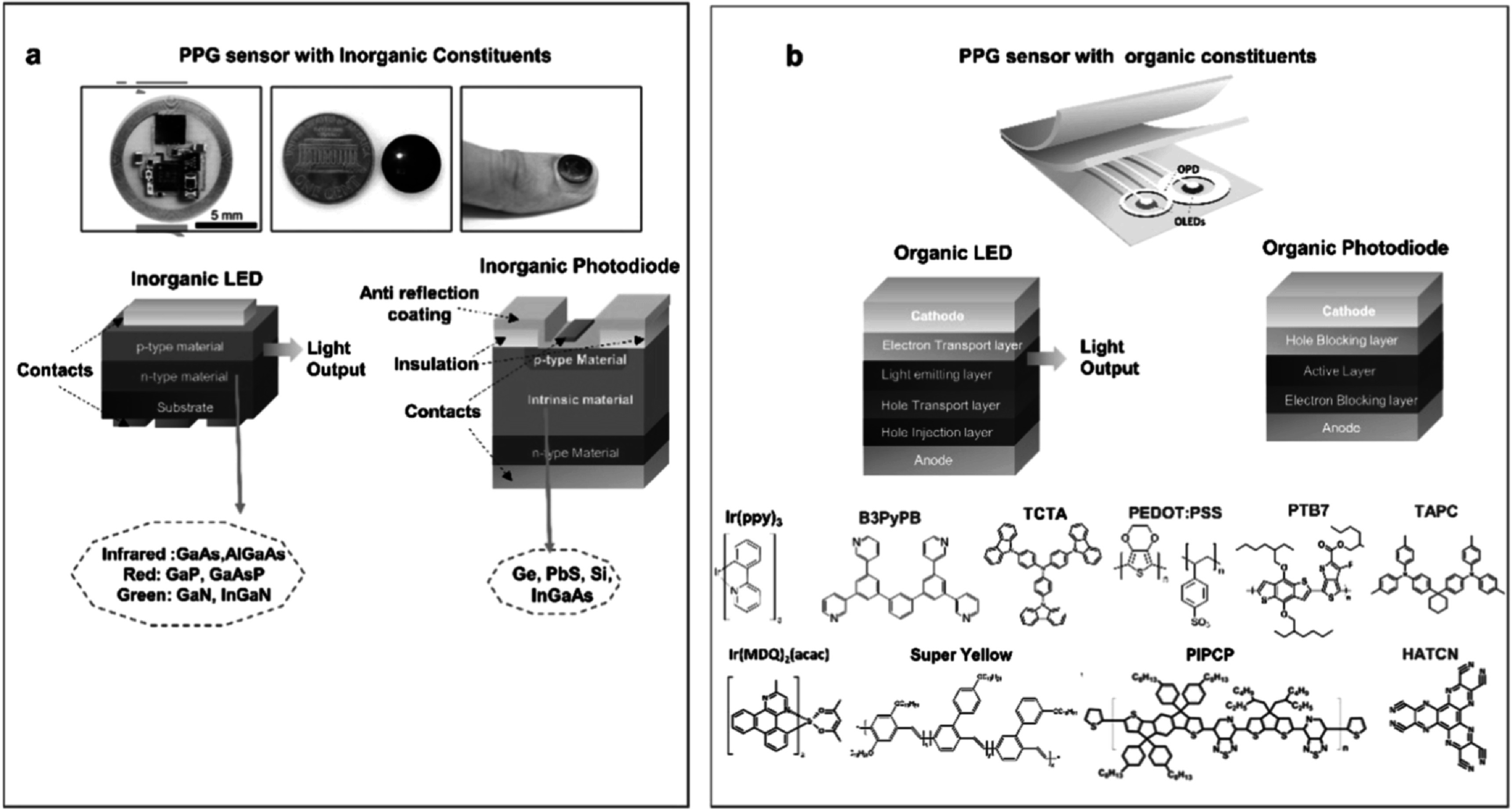
Device structure and constituent materials of inorganic and emerging organic PPG sensors. (a) Image of an inorganic PPG sensor and conventional structure of inorganic LEDs and PDs. (b) Schematic of a representative organic PPG sensor and device structure of OLEDs and OPDs along with commonly used materials for various layers (Ir(ppy)_3_: green light emitting material, B3PyPB: electron-transport/hole blocking material, TCTA: hole-transport and hole-injection material, PEDOT:PSS: hole transport material, PTB7: active layer of OPD, TAPC: hole-transport material, Ir(MDQ)_2_(acac): orange-red light emitting material, Super Yellow: light emitting material, PIPCP: active layer in near-IR. detector, HATCN: hole-injection material). Sources: (a) is adapted from Kim *et al* (2017) with permission (Copyright 2016, John Wiley and Sons).

In the last decade, PPG sensors have transcended their necessity in the health sector as they entered the burgeoning industry of wearable technology being an integral part of fitness tracking gadgets like smartwatches or wristbands. Most commercial PPG sensors in such wearable devices utilize traditional LEDs (inorganic III–V compound semiconductor-based LEDs) as light source and silicon photodiodes as the photodetector, which are limited by their rigidity, bulky nature, and high expense associated with scalable fabrication. To tackle these issues, researchers have been working on developing new types of flexible PPG sensors based on various emerging material technologies which can ensure skin conformability, the versatility of design, lower power consumption, mass-production scalability, and an overall improved user experience. Such flexibility is of great importance in neo-natal and infant care where inconspicuous and flexible sensors can significantly aid in monitoring vital signs of the delicate organs which could otherwise be discomforted by rigid electronic components. Organic materials are particularly relevant in this regard due to their inherent mechanical softness enabling the formation of flexible, lightweight organic optoelectronic devices prepared with solution processing, printing, or coating techniques that allow freedom in design. Figures 2(a) and (b) represent the device structure of PPG sensors with organic and inorganic constituents along with the chemical structure of commonly used organic compounds in recent devices.

The first all-organic transmission mode PPG sensor with an organic photodiode (OPD) and organic LEDs (OLED) was reported by Lochner *et al* (2014). For their pioneering device, they used bulk heterojunction solution-processed OPD consisting of PTB7:PC_71_BM, and polyfluorene-based printed green and red OLEDs having emission peaks at 510 nm and 632 nm, respectively. Bansal *et al* (2015) demonstrated a reflective organic PPG sensor using an alternative approach with a single OLED that emits in both red and near-infrared regions, and two OPDs with filters that distinguish between the two relevant spectra. Subsequently, researchers worked on making the sensor more skin conformable by reducing thickness, eliminating bulky electronic interfaces, improving the efficiency of the device, or enabling the use of near-infrared (NIR) LEDs.

An ultra-flexible photonic PPG sensor with only 3 *μ*m thickness and capable of withstanding high compressive strain was developed by researchers at Tokyo University using green and red Polymer LEDs (PLEDs) (Yokota *et al* 2016). Later they demonstrated a PPG sensor using near-infrared (IR) OPDs based on narrow-bandgap PIPCP polymer (Park *et al* 2018). A self-powered PPG sensor has been demonstrated recently by their group that uses an organic photovoltaic (OPV) module to power a single PLED emitting in the green/yellow region of the spectrum with the polymer Super yellow as the emission layer (Jinno *et al* 2021).

In order to achieve flexibility, J A Rogers’ team focused on miniaturizing the inorganic sensor rather than using soft mechanics as they developed a near field communication (NFC) enabled wireless optoelectronic system for PPG monitoring (Kim *et al* 2017). The use of NFC technology for power delivery and data transmission along with serpentine interconnects allowed them to achieve flexibility while maintaining small form factors due to the absence of bulky electronic interfaces as used in previous organic PPG sensors. Li *et al* (2017) demonstrated a flexible inorganic pulse oximeter using specific strain-isolation design, nanodiamond thinning, and hybrid transfer printing of LEDs, PDs, and interconnects.

Arias and coworkers leveraged the scalability of organic sensors to create a 2D oxygenation map of an organ using an organic reflectance oximeter array (Khan *et al* 2018) and later developed a PPG sensor that can utilize ambient light, thus eliminating the need for OLEDs and their driving circuitry (Han *et al* 2020).

Due to the design versatility of OLEDs and OPDs, researchers can explore various sensor configurations to find the combination that uses the least amount of power and has the best signal-to-noise ratio. Such design freedom was utilized by Lee *et al* (2018b) to develop a wrap-around OPD layout in which an ‘8’ shaped OPD surrounded the LEDs, ensuring the complete utilization of the light from OLEDs and thereby obtaining highly efficient operation with less than 50 *μ*W power consumption. Recently their group utilized a hybrid sensor by combining inorganic LEDs with OPDs that consumed below 35 *μ*W power and maintained stable operation for more than 40 d (Lee *et al* 2021a).

### Current and future challenges

**Figure 3.**
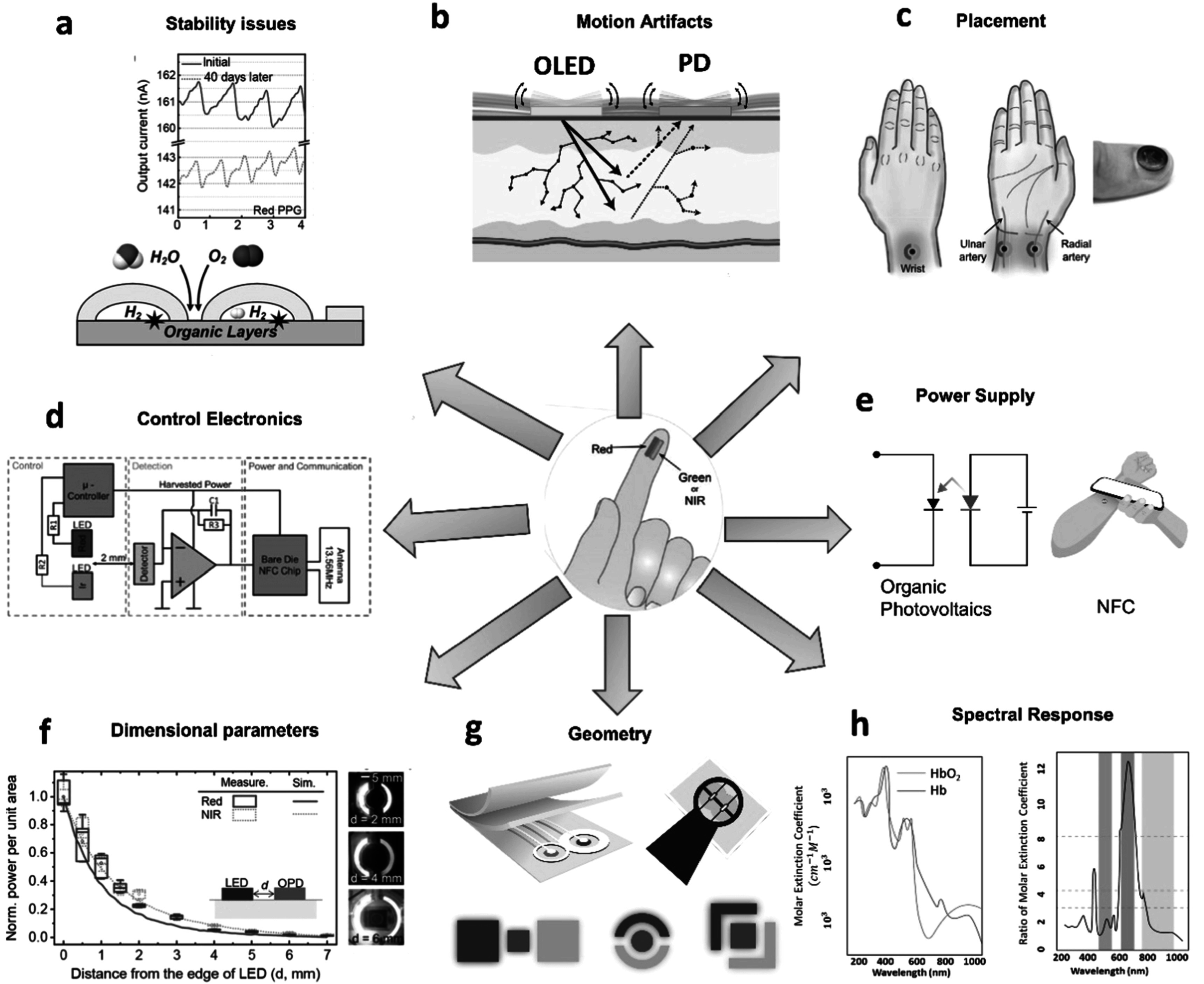
Potential aspects and issues addressed through the incorporation of emerging materials in PPG sensors- (a) Ensuring stable operation through the elimination of environment-caused degradation. (b) Addressing motion artifact-related noise in PPG signal. (c) Optimal placement of the sensor. (d) Design of flexible control circuits. (e) Mode of powering such as wireless power transmission or organic battery technology. (d) Design of flexible control circuits. (e) Mode of powering such as wireless power transmission or organic battery technology. (f) Optimization of dimensional parameters such as distance between source and detector. (g) Innovation in geometry to maximize efficiency. (h) Choice of suitable light source considering the extinction properties of blood and organs. Sources: (a) Reproduced from Lee *et al* (2021a) Copyright 2021, AAAS. (c) Reproduced from Khan *et al* (2019) (CC BY 4.0); and Kim *et al* (2017) Copyright 2016, John Wiley and Sons. Khan *et al* (2019) refers to: Khan *et al* ‘Organic multi-channel optoelectronic sensors for wearable health monitoring’, IEEE Access, 2019, https://doi.org/10.1109/ACCESS.2019.2939798 (CC BY 4.0). (d) Reproduced from Kim *et al* (2017) Copyright 2016, John Wiley and Sons. (f) Adapted from Lee *et al* (2021a) Copyright 2021, AAAS; and Khan *et al* (2019) (CC BY 4.0).

PPG sensors using emerging and organic materials are still at the research stage. Various aspects of these sensors need to be addressed for their widespread commercialization. Some such challenges hindering their growth are discussed in this section and illustrated in figure 3.

#### Ensuring stable operation

A significant obstacle to the long-term usage of flexible PPG sensors is their insufficient operational stability under ambient conditions. Due to the reaction of constituent organic molecules in LEDs and PDs with water and oxygen molecules in the air, particularly at the electrode interface, the PPG signal quality can significantly deteriorate over time (figure 3(a)). Yokota *et al* (2016) used a passivation layer consisting of SiON and Parylene in their photonic sensor that showed stable operation for up to 4 d. This duration is still very low compared to inorganic PPG sensors. Particularly for OLEDs, the degradation is accelerated by high-brightness operation.

#### Noise reduction

Skin-conformable PPG sensors suffer from various degrees of noise, such as electrical, optical, and mechanical noise due to poor contact between sensors and substrates during deformation, thermal noise due to body heat, electromagnetic interference, and motion artifacts (figure 3(b)). Frequencies in a typical PPG waveform range from 0.5 to 4 Hz, hence a suitable bandpass filter can be used to remove high-frequency noises beyond this range, such as thermal or electrical noise. The frequency band of motion artifacts caused by organ movement or sensor displacement overlaps with the PPG signal as they range from 0.1 to 10 Hz. Filtering, therefore, fails to effectively eliminate motion distortions from the measured signal. Baseline wandering is another source of error in the measurement of the AC component of the PPG signal caused by respiration, sympathetic nervous system activities, and thermoregulation. Parasitic current in the OPD due to ambient light also interferes with PPG measurement. Eliminating these noises to improve the SNR of the PPG signal is necessary to establish the reliability of measurement.

#### Biosafety and durability

It is necessary to ensure that continuous long-term use of the sensors does not cause irritation, inflammation, or discomfort and that the sensors are protected from degradation due to the accumulation of skin cells and are water-resistant. The heating of the body part during the operation of the sensor must also be minimized. Optimal placement of sensors to ensure minimal discomfort while maximizing signal quality is a major challenge (figure 3(c)).

#### Need for flexible interface

Most of the organic PPG sensors developed till now require an analog front end for driving the LEDs, data acquisition, and a battery system for power delivery along with hard-wired connections. These are made of rigid inorganic components, which ultimately limit the form-factor minimization of organic sensors. Depending on the driving cycle and device active area the driving current of OLEDs and PLEDs can significantly vary within 0.5–10 mA cm^−2^. The driving voltage usually lies between 1.5 and 9 V with its nominal value being around 5 V. Efficient and flexible circuitry compatible with the high drive current and voltage requirements of organic electronics is necessary to truly achieve the advantages of emerging materials in PPG sensors (figure 3(d)).

#### Improving power conversion efficiency

To reduce heat production and eliminate the requirement for a large inorganic battery, which restricts the size and form factor of PPG sensors, operational power must be reduced and leakage power must be minimized during standby mode. Self-powered PPG sensors with built-in photovoltaic cells can help in this regard (figure 3(e)). The utilization of PPG sensors for continuous PPG monitoring ultimately depends on achieving improved efficiency of the constituent LEDs and PDs, which requires innovations in design parameters such as sensor dimension and geometry, as shown in figures 3(f) and (g). Ensuring desired spectral response from the designed LEDs and PDs to maximize power utilization is another important challenge to be considered (figure 3(h)).

### Advances in science and technology to meet challenges

Considering the aforementioned challenges, innovative measures need to be taken for long-term, widespread use of PPG sensors, which are summarized below.

1. The formation of a thin and high-quality passivation layer on a flexible substrate is an essential requirement to improve the stability of PPG sensors. Suitable encapsulation technologies that can protect the organic devices from ambient air and moisture while not blocking sweat glands can also lead to stable operation. Yokota *et al* (2016) recently reported an inverted PLED with a novel electron injection layer that significantly improved stability in ambient light without requiring any passivation layer. Development in such material technologies can further contribute to stability enhancement. Other approaches to tackle the stability issue can be the usage of inorganic LEDs or ambient light sources instead of the OLEDs, which are the main bottleneck for prolonged operation.
2. High-frequency noise in PPG signals can be eliminated using suitable filtering algorithms. Using black ink on LED walls can eliminate the noise due to the direct coupling of OLED and OPD. Adding redundancy to measurements with the multi-channel operation (Khan *et al* 2019) or using utilizing the polarizability of scattered light (Hwang Lee *et al* 2022) are some of the hardware-based approaches in motion-artifact reduction. Various signal processing algorithms such as adaptive filtering, independent component analysis, or wavelet-based techniques are also being employed to denoise PPG signals from motion artifacts. Baseline drift can be attenuated with estimation techniques, high pass filtering, or interpolation methods. The accuracy of these methods along with their computation complexity poses a major challenge for the research community.
3. Development in material and encapsulation technology is required to ensure sensors that do not cause irritation or discomfort to the user. Optimized power utilization also needs to be ensured to reduce thermal discomfort. It is also necessary to ensure consistent sensor performance for different demographics irrespective of their color, race, or physique.
4. The efficiency of emerging PPG sensors can be improved through the effective use of light by utilizing innovative geometry of the LEDs and OPDs such as bracket, annular, or ‘8’ shaped wrap-around geometry. Optimization of device dimension, shape, OLED-OPD distance, the effect of placement, and other aspects of power consumption provides a wide-ranging area for future study. Furthermore, by utilizing ambient light from the sun or conventional light sources with spectrally selective OPDs, the need for LEDs and associated power to drive them can be eliminated, as demonstrated by Han *et al* However, their ambient light PPG sensor had restricted usage compared to conventional sensors which must be addressed in future research.
5. Development of flexible, compact, and lightweight interconnects, ICs, batteries, and voltage regulators are required to utilize organic sensors’ form factor advantage to the full. Wireless NFC-enabled transceivers and power delivery systems, self-powered devices with organic PV, and body-coupled energy harvesting could represent potential approaches to facilitate PPG sensors with overall compact form factors.
6. By utilizing novel material technologies for LEDs and Photodiodes, PPG sensor design can be improved. Perovskite LEDs, in contrast to organic semiconductors, constitute a low-cost strategy because of their inexpensive solution processability (Tan *et al* 2014). However, further improvement in their electroluminescence efficiency is needed to obtain a viable signal which could be utilized in PPG applications. Colloidal Quantum dot LEDs have the added advantages of bandgap tunability and high external quantum efficiency (Shirasaki *et al* 2013), which make them ideal for multi-wavelength PPG applications. The detection of PPG signals over a wide range of wavelengths can be greatly improved by photodetectors with enhanced photo-response achieved by using plasmonic nanoparticles (Liu *et al* 2011). Further research into the use of graphene and other 2D semiconductors in these PDs is needed to enhance the photo-response and subsequently the quality of the PPG signal.

## Concluding remarks

To summarize, the opportunity to obtain a stable, highly efficient, ultra-flexible PPG sensor that can be seamlessly integrated into daily life requires further comprehensive research. Innovation in LEDs, photodiodes, and control circuitry through new material technology and efficient circuit design can significantly aid in achieving the final goal.

## 3. In-ear photoplethysmography for respiratory monitoring

### Harry J Davies and Danilo P Mandic

Department of Electrical and Electronic Engineering, Imperial College London, London, SW7 2AZ, United Kingdom

### Status

**Figure 4.**
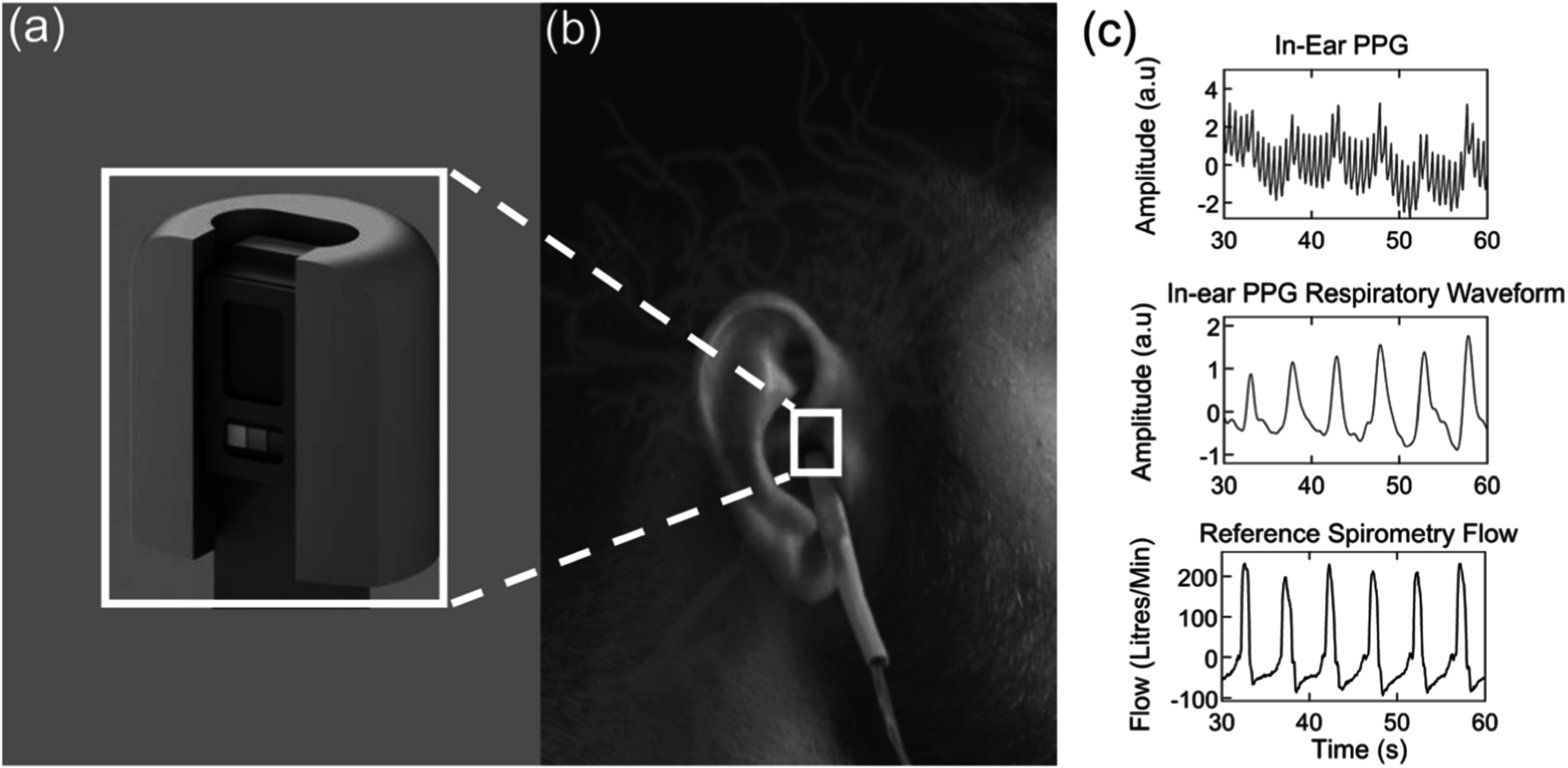
The in-ear PPG sensor with positioning highlighted and sample respiratory modulations shown. (a) A zoom in of the in-ear PPG sensor. (b) The placement of the sensor within the ear canal with arteries supplying the brain highlighted in red. (c) Exemplar in-ear PPG waveforms with 1:3 inspiration to expiration, with the raw in-ear PPG in blue, the conditioned respiratory waveform in red and the reference spirometry flow in grey. Adapted from: Davies *et al* (2020) and Davies *et al* (2022).

In the past decade, so called *hearables* have been introduced as an alternative to conventional wrist-worn wearables, with the functionality of several physiological recording modalities from electroencephalography (EEG) and electrocardiography (ECG) to photoplethysmography (PPG). In-ear PPG, introduced in the literature as a measurement site in 2007 (Vogel *et al* 2007), and the corresponding blood oxygen estimates (SpO_2_), offer many advantages over conventional wrist and finger recording sites. A major obstacle to wearable PPG is constriction of peripheral blood flow that occurs with low temperatures, but in-ear PPG has been shown to be resistant to these effects due to the characteristic of the ear canal (Budidha and Kyriacou 2018). Moreover, in-ear SpO_2_ has shown high accuracy in the detection of hypoxia (Venema *et al* 2012), while the proximity to the carotid artery which supplies the brain with oxygenated blood enables in-ear probes to measure oxygen at minimal delay when compared with finger based SpO_2_ probes (Davies *et al* 2020). Importantly, in-ear PPG has been shown to have stronger respiration induced intensity variations than the finger by an order of magnitude (Budidha and Kyriacou 2018) (Davies *et al* 2022) together with stronger respiratory pulse amplitude variations (Davies *et al* 2022), as illustrated in figure 4. These properties are likely due to the high density of vasculature at the ear, which similarly enables the use of miniaturised low power PPG chips.

### Current and future challenges

Leveraging the detailed respiratory variations that can be recorded from in-ear PPG has enabled the classification of chronic obstructive pulmonary disorder (COPD) against non-obstructive respiratory diseases, and healthy controls, both older and younger (Davies *et al* 2022). This was achieved by measuring the imbalance between inspiration and expiration that occurs with obstructive lung disease, given the exaggerated effect of obstruction on expiration. Whilst respiratory information from PPG tends to focus on respiration rate and thus peak timing, this discovery goes a step further by treating inspiration and expiration as two separate entities both in timing and amplitude. A significant limitation of this method, however, is the impact of motion artefacts. Although motion artefacts also impact heart rate and respiratory rate estimation, recovering peak timing in the presence of motion is more straightforward than the amplitude or specific waveform characteristics. For this reason, classification of COPD from in-ear PPG only works well when the subject is still, and the same applies more broadly to recordings of in-ear SpO_2_.

Truly wearable monitoring of respiratory diseases requires both accurate and sensitive oxygen measures and respiratory information that can be recorded consistently during movement, rather than just at rest. This is particularly important in the case of severe respiratory diseases, where oxygen levels can decrease during movement and their detection can inform key decisions around patient treatment. Similarly, combining oxygen desaturation measures with detailed PPG derived respiratory waveforms would likely increase classification accuracy and value to clinicians. Current in-ear probes somewhat address this issue with different sized ear buds to ensure a good fit in all ear canals, but much future work needs to be accomplished to enable truly 24/7 wearable monitoring of in-ear PPG.

### Advances in science and technology to meet challenges

**Figure 5.**
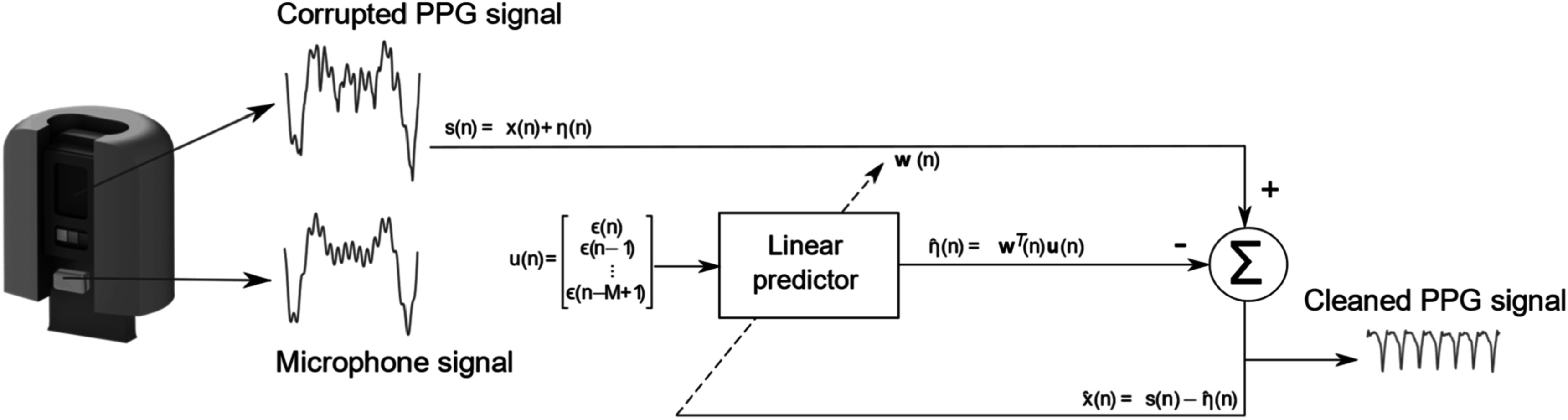
Overview of the adaptive noise cancellation of in-ear PPG with a micro-electrical-mechanical systems (MEMs) microphone used as a noise reference to detect minor motion between the sensor and the skin. Source: Adapted from Davies *et al* (2020) (CC BY 4.0).

To achieve stable continuous in-ear PPG in the presence of artefacts, significant advances are needed in both hardware and signal processing to preserve the exact pulse and respiration induced intensity variation waveforms. Given this, key directions for the future development of in-ear PPG include the stabilisation of hardware within the ear canal, and the online removal of artefacts via an appropriate reference signal that is highly correlated with the artefacts. Stabilising the PPG sensor with respect to the skin is straightforward with an adhesive outside of the ear canal, but within the ear canal this method becomes impractical. Rather than stabilising a single sensor, one potential avenue involves the combination of many light emitting diodes and photodetectors on a single ear bud, allowing algorithms to dynamically select channels with the best contact at any given time point. A viable method of artefact removal is to employ an additional sensing modality that correlates with the artefact but not with the signal of interest, and in turn this sensor can be used as a reference to remove all artefact-correlated signal from the raw signal, leaving the physiological signal of interest. Existing literature has focused on using accelerometers to provide this reference and remove artefacts, such as those that arise from walking, but artefacts that impact ear sensors tend to occur from smaller movements such as that of the jaw. Specifically, the actions of talking, chewing and general head movements present major artefacts to ear canal derived physiological signals. To this end, micro-electrical-mechanical systems (MEMs) microphones, capable of detecting minor movements between the sensor and skin, have proven effective at removing these ear-specific motion artefacts from ear-EEG (Goverdovsky *et al* 2015). By the same principle, the use of MEMs microphones may provide an effective artefact reference and thus enable a stable 24/7 in-ear PPG trace. This principle is outlined in figure 5.

### Concluding remarks

The in-ear location for PPG provides low latency blood oxygen estimates and highly detailed breathing waveforms. The combination of these physiological properties with next generation signal processing techniques and multimodal sensing has the potential to revolutionise the wearable monitoring of chronic respiratory disorders.

## Acknowledgments

They authors give special thanks to the UKRI PhD scholarship funding, and support from the MURI/EPSRC grant EP/P008461.

## 4. Wearable multi-wavelength photoplethysmography

### Jing Liu^1^, Daniel Franklin^2^ and Ni Zhao^3^

^1^Analog Devices Inc

^2^Institute of Biomedical Engineering, Translational Biology & Engineering Program, Ted Rogers Centre for Heart Research, University of Toronto

^3^Department of Electronic Engineering, The Chinese University of Hong Kong

### Status

**Figure 6.**
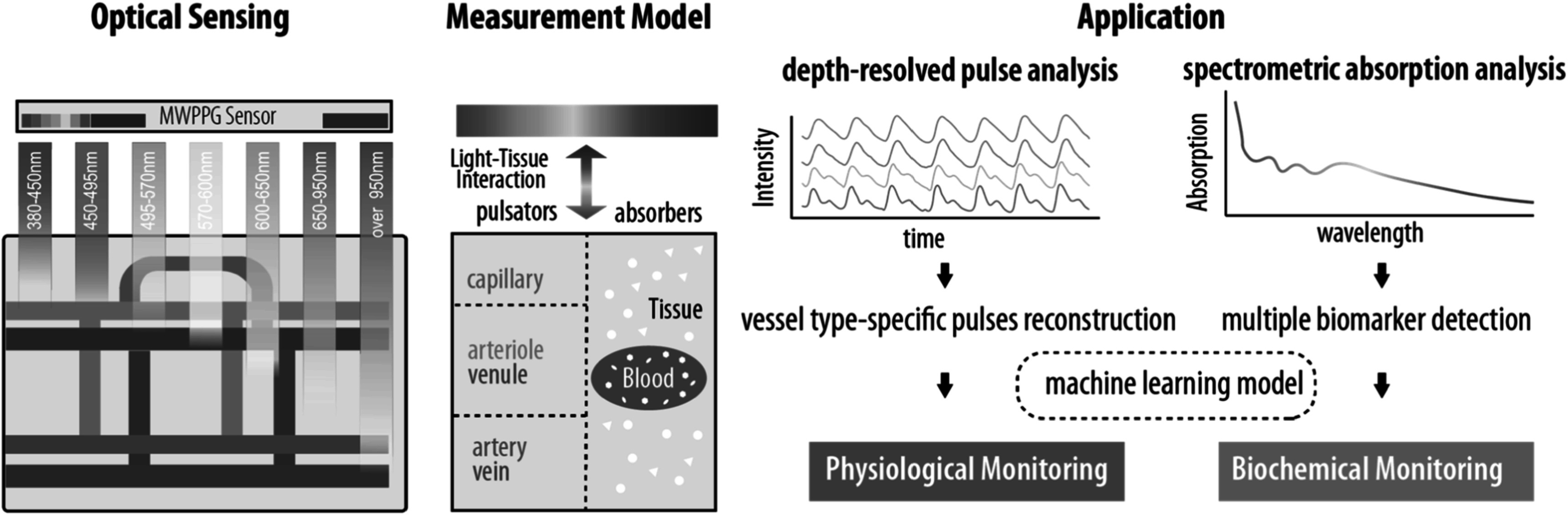
Multi-wavelength photoplethysmography (MWPPG) system development workflow.

Multi-wavelength photoplethysmography (MWPPG) sensors emit and detect multiple wavelengths of light to non-invasively characterize underlying tissue. As summarized in figure 6, the MWPPG technique investigates the wavelength-dependent optical responses from the skin and further captures the depth-resolved blood pulsation for advanced hemodynamic monitoring and the tissue absorption spectrum as a step toward wearable biomedical spectroscopy, thus expanding the functionality of PPG-based healthcare systems.

The skin has a layered structure perfused by capillaries in the papillary dermis, arterioles and venules in the dermal layer, and arteries and veins in the subdermal layer. When MWPPG includes well-separated wavelengths differing significantly in penetration depths, the pulsatile waveforms (AC) of MWPPG, referred to as pulsatile MWPPG here, can be used to characterize vessel type-specific pulses originating from different skin depths. It has been observed that pulsatile MWPPG channels differ in morphologies and trajectories in response to contact pressure, reactive hyperemia, breath holding, etc (Gailite *et al* 2008, Liu *et al* 2016, 2019). The phase shift between MWPPG of well-separated wavelengths has been demonstrated to be physiological and also reasoned to indicate the pulse transit time (PTT) along skin arterioles (Vahdani-Ma and Vahdani-Manaf 2015, Liu *et al* 2019). MWPPG-derived arteriolar PTT has been theoretically and experimentally revealed to correlate with systemic vascular resistance and utilized for cuffless blood pressure prediction (Liu *et al* 2019, 2021a, Lu *et al* 2022). Integrating MWPPG with the oscillometric blood pressure measurement technique also allows for novel non-invasive capillary and arterial blood pressure (Liu *et al* 2021b).

Increasing the number of wavelengths for spectroscopic MWPPG also enables biomarker detection and quantification beyond the classic SpO_2_ measurement, such as methemoglobin and carboxyhemoglobin in portable co-oximeters and bilirubin in jaundice meters. Additional wavelengths can also increase the accuracy of pulse oximetry estimations for a diverse pool of patients where melanin and fat content can widely vary. The endpoint of this line of thought is advancing the MWPPG technique to implement a wearable spectrophotometry solution that can quantify any biomarker with a distinctive absorption spectrum.

In recent years, PPG sensors have gained increasing popularity in commercial wrist-worn health trackers with a wide use of green light for heart rate measurement and red and IR light for SpO_2_ measurement, which can serve as a generic MWPPG sensing solution. There are extensive ongoing research efforts in designing MWPPG sensors with innovative optical layouts and high-performance multi-wavelength/imaging components to enhance signal acquisition (Yokota *et al* 2021). Therefore, it is promising to develop and apply novel MWPPG-based health monitoring concepts to future wearable devices.

### Current and future challenges

Increasing the number of wavelength channels within a MWPPG sensor presents engineering challenges depending on the multiplexing scheme implemented. Two main schemes exist: source multiplexing, where numerous narrow-band emitters are paired with a broad-band detector and synchronized LED-driving/read-out circuitry cycle between wavelengths to create a time-interlaced signal, and detector multiplexing, where numerous narrow-band detectors are paired with a broad-band emitter to read out multiple wavelength channels in parallel (Hossain *et al* 2021, Wang *et al* 2022). Source multiplexing schemes face a fundamental trade-off between the number of wavelength channels and sampling rate, whereas detector multiplexing systems face limitations in size and/or sensitivity as the number of sensing elements is increased. More channels in MWPPG, in principle, enable higher spatial/spectral resolution and a higher sampling rate allows for better temporal resolution for time-domain features such as MWPPG phase delay. However, an additional burden is placed on the limited power, memory, and computational resources of wearable devices. Maintaining acceptable signal quality for all MWPPG channels in real-world measurement is an even greater challenge, particularly when simultaneous recordings are required. This is a practical issue for MWPPG measurement at the wrist, which suffers from joint/tendon movement and lower vascular density.

Despite the reference values of penetration depths as a function of wavelength in the literature, the exact constitutions of MWPPG can vary intra- and inter- individually with the skin tone, measurement site, and perfusion conditions, thus affecting the optimal wavelength combination and adding uncertainties to the MWPPG-derived features. Reconstruction of the original vessel type-specific pulses from pulsatile MWPPG signals is a difficult blind source separation problem since the source pulsations such as the capillary pulse, arteriolar pulse, and arterial pulse (or otherwise defined by the measurement model) bear resemblance in their morphologies and frequency spectrums. Novel optical sensing layouts and schemes and intelligent compensating algorithms will be needed to improve the measurement robustness.

Light sources of proper wavelength resolution and high sensitivity photodetectors are hardware requisites for reliable measurements, particularly in spectroscopic MWPPG sensing for biomarker detection. Epidermal spectroscopic MWPPG measurement of biomarkers faces challenges in measurement sensitivity and specificity due to low concentration of biomarkers, interfering molecules, and spectral overtone, as well as technical challenges such as identification of optimal wavelength combinations and suitable chromophores and model calibrations for uncertain internal factors and environmental factors such as skin tones, temperature, and humidity.

### Advances in science and technology to meet challenges

High-performance optical sensors with tailored sensing schemes can assist the acquisition of high-quality MWPPG signals. Flexible electro-optical sensors provide better conformity with the skin surface, thus facilitating motion-resistant and long-term health monitoring (see section 2) (Xu *et al* 2017, Yokota *et al* 2020). To advance MWPPG to wearable spectrometric measurement, high sensitivity, speed, and efficiency are desired for broad-band photodetectors for source multiplexing and narrow-band photodetector for detector multiplexing.

To achieve a robust and efficient system for physiological monitoring, it is worth either: extending the MWPPG sensor into a sensing array wherein the geometry of light sources and detectors, such as the optimal separation distance, is carefully designed; or, integrating MWPPG with motion, pressure, acoustic and electric sensors to develop a multi-modality sensing solution. Fusing the multi-modality bio-signals prevents misleading outputs from motion-corrupted MWPPG signal segments or allows for adaptive power-saving sensing strategies based on the instantaneous use case. While a single-node MWPPG sensor provides the convenience of easy wearability, it is beneficial to adopt a body sensor network setup with multiple sensing nodes to achieve a future comprehensive assessment of systemic health conditions.

Further theoretical and experimental deep dive into the physiological origins can MWPPG in various use cases facilitate identifying and extracting relevant and indicative features. Classic PPG-based applications such as heart rate, arrhythmia, and respiration detection can take advantage of the rhythmic differences and landmark robustness in pulsatile MWPPG. Often it is challenging to explicitly express the mathematical relationship between the MWPPG-derived features and the target variables due to the inter-/intra- individual tissue variances and the complexity of the light-tissue interaction process. Machine learning and artificial intelligence models leveraging proper medical datasets are promising paths to formulate the relationship from the high-dimensional MWPPG-derived features to the target biometrics.

### Concluding remarks

Given the widespread use of PPG sensors in wearable healthcare devices, MWPPG is a compatible solution to probe enriched and profound hemodynamic information without significantly increasing the sensor dimension. Pulsatile MWPPG enables depth-resolved analysis, thus improving the accuracy of traditional PPG-based applications, especially when vessel type-specific pulse waveforms are desired. Spectroscopic MWPPG is promising for wearable biochemical analysis and biomarker detection leveraging advances in high-performance optical components. A systematic effort from sensing, modeling and algorithms are demanded to achieve practical MWPPG healthcare applications.

## Acknowledgments

The authors acknowledge financial support from General Research Fund (RGC Ref No. 14209620) from the Research Grants Council of Hong Kong. Dr Franklin is the Ted Rogers Chair in Cardiovascular Engineering, University Health Network, University of Toronto.

## SIGNAL PROCESSING

## 5. Pulse rate variability

### Elisa Mejía-Mejía and Panicos A Kyriacou

City, University of London

### Status

Pulse rate variability (PRV) describes the changes in pulse rate through time and is measured from pulsatile signals, especially the photoplethysmogram (PPG). It has been used to assess cardiac autonomic activity and multiple mental and somatic diseases (Mejia-Mejia *et al* 2020a). It has been suggested as an alternative to heart rate variability (HRV), which has been largely explored in the last 40 years for evaluating changes in the cardiac autonomic nervous system (ANS) and related diseases (Mejia-Mejia *et al* 2020b). The rate at which the heart beats is normally determined by the sinus node in the heart, which is controlled by the sympathetic and parasympathetic branches of the ANS. Hence, changes in heart rate indirectly reflect the behaviour of the cardiac control exercised by this system (Task Force of the European Society of Cardiology and The North American Society of Pacing and Electrophysiology 1996). HRV analysis has been used in the understanding, detection and diagnosis of various cardiovascular diseases and other pathophysiological phenomena, such as diabetes, hypertension and atherosclerosis (Mejia-Mejia *et al* 2020a).

**Figure 7.**
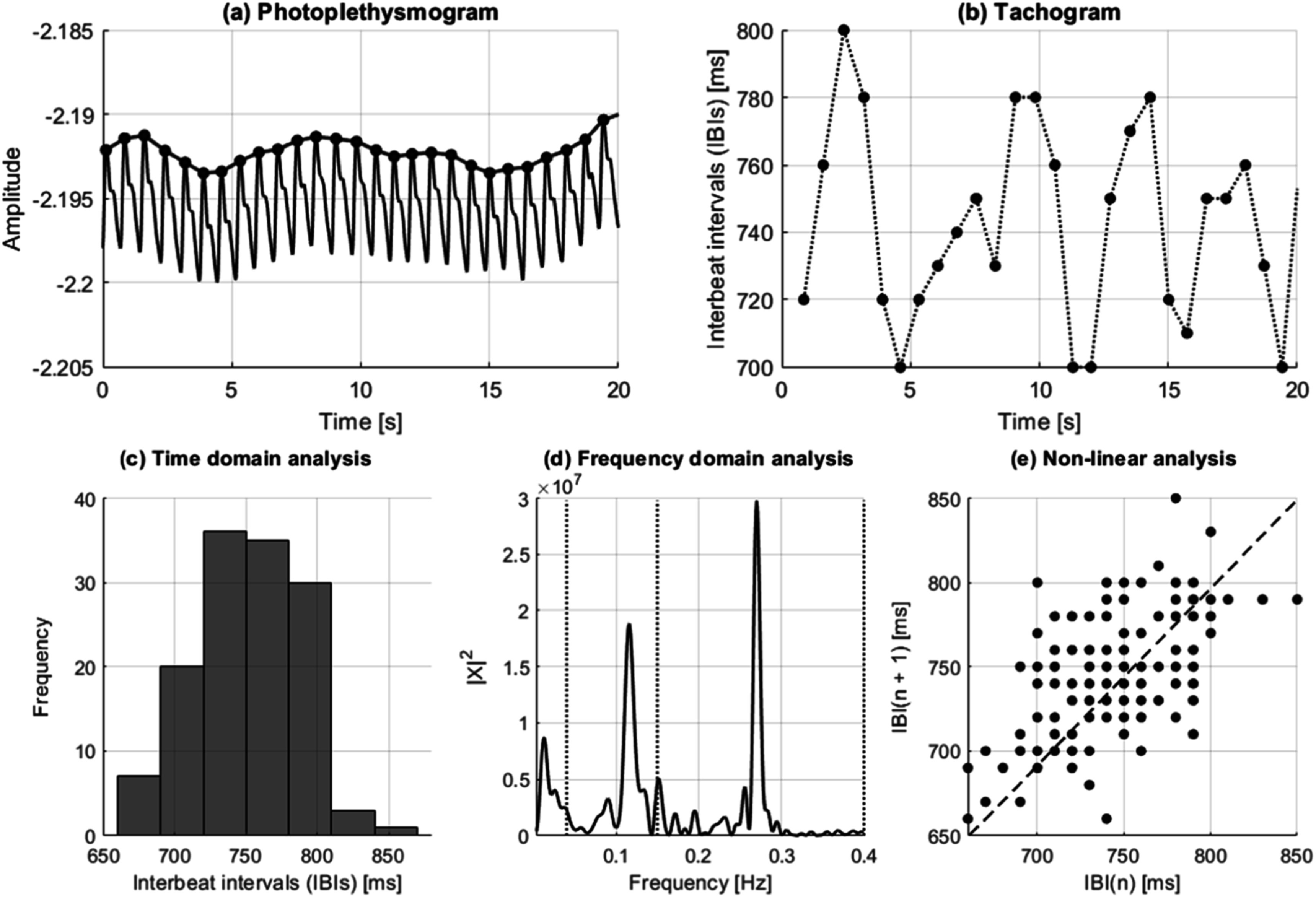
Pulse rate variability analysis from photoplethysmograms. Initially, (a) interbeat intervals (IBIs) are detected from the pulsatile signal, and (b) the duration of the intervals is extracted and plotted against time. These IBIs can be summarised using (c) time-domain features, (d) frequency-domain indices, and (e) nonlinear analyses.

Since HRV analysis depends on acquiring electrocardiographic (ECG) signals continuously, which can be cumbersome and difficult to implement in real-life scenarios, researchers have used the PPG as an alternative source of pulse rate measurements, given that it is a non-invasive, non-intrusive, simple, and low-cost technique (Kyriacou 2021). PRV analysis has been explored for the analysis of autonomic changes under different conditions, such as the presence of mental or somatic diseases, during sleep, or for evaluating the effects of drugs (Mejia-Mejia *et al* 2020a). PRV seems like a logical alternative to HRV since PPG signals carry valuable information regarding cardiovascular parameters and are easier to acquire, compared with ECG, in a long-term manner and in real-life scenarios. Hence, PRV could represent a useful tool for assessing cardiovascular and autonomic health in everyday settings, both in healthy and ill subjects. Also, given the widespread use of PPG sensors in wearable devices, it could become the ideal alternative to ECG in assessing different pathologies, including mental and cardiovascular diseases. Figure 7 illustrates the process of performing PRV analysis with PPG signals.

### Current and future challenges

Although HRV and PRV originate from similar processes, and pulse rate has been found to be a good surrogate of heart rate, the relationship between HRV and PRV is not straightforward, and there is still no consensus regarding the validity of using PRV as a surrogate of HRV (Mejia-Mejia *et al* 2020a). It has been shown that several technical and physiological factors may affect PPG and could alter PRV. Some researchers argue that the differences between HRV and PRV are mainly due to physiological aspects, such as haemodynamic changes (Gil *et al* 2010, Mejía-Mejía *et al* 2021), the different nature of PPG and ECG signals (Schäfer and Vagedes 2013), and the effects of other factors on PRV, such as external forces on the arterial vessels (Gil 2010). Moreover, PRV has been found to be present in the absence of HRV (Constant *et al* 1999, Pellegrino *et al* 2014), and there are reports of differences in PRV between measurement sites (Yuda *et al* 2020). All of these suggest there are different processes affecting PRV that are not necessarily related to HRV. Whilst HRV is the main determinant of PRV, PRV is also influenced by variations in pulse arrival time: the time between R-waves in the ECG signal (from which HRV is assessed), and pulse onsets in the PPG signal (from which PRV is assessed). Indeed, studies have shown that differences between PRV and HRV are due to oscillations in pulse arrival time (Ajtay *et al* 2023) and pulse transit time (pulse arrival time minus the pre-ejection period) (Gil *et al* 2010). Pulse arrival time, pulse transit time, and also respiration, can have a sufficiently large influence so as to disturb some key measurements from PRV, especially under non-ideal scenarios, such as when non-healthy, older, and non-resting subjects are considered. It would be beneficial to pursue research that elucidates the level of impact these factors have on PRV and its relationship with HRV, and how could both PRV and HRV be used to extract information about these other physiological processes taking place.

Other studies have concluded that the agreement between PRV and HRV may also be affected by technical aspects relating to the extraction of PRV from pulse waves (Béres *et al* 2019, Peralta *et al* 2019, Mejía-Mejía *et al* 2022). The manner in which PPG signals are acquired and processed for measuring PRV is also important. Photoplethysmography, being an optical technique, is based on the interaction between tissue and light, and the wavelength at which the tissue is illuminated affect the depth at which light penetrates, with longer wavelengths reaching deeper tissue (Kyriacou 2021). PRV has been extracted from PPG signals acquired using red, infrared, green and orange lights, but it is still not clear if this could result in differences in PRV measurements. Other important aspects that could affect PRV results are the PPG sampling frequency, the manner in which PPG signals are pre-processed, and the choice of frequency domain analysis techniques used for PRV analysis (Schäfer and Vagedes 2013, Mejia-Mejia *et al* 2020a).

The main challenges for PRV analysis are: (1) understanding how the above-mentioned aspects affect PRV analysis and results; and (2) reaching a clearer understanding of how different physiological phenomena affect PRV and its relationship with HRV.

### Advances in science and technology to meet challenges

PPG sensors are the main technology needed to obtain PRV information. However, and as with most techniques based on PPG, there is a lack of standardisation of the technique and the signal processing strategies used to extract PRV from PPG signals. This lack of standardisation suggests that the comparison and validation of PRV-related results is not possible, hence decreasing the reliability of the technique. It is important to establish guidelines for standardising PRV estimation, similar to the standardisation of HRV measurements (Task Force of the European Society of Cardiology and The North American Society of Pacing and Electrophysiology (1996), Shaffer and Ginsberg, 2017). This would help in avoiding equivocal comparisons of results from similar studies without having to consider processing and acquisition differences, which may significantly affect the conclusions reached by various PRV studies.

Also, since it has been observed that several physiological phenomena may affect PRV differently from HRV, it is important to perform basic research to identify the different processes that influence PRV. This could not only make the technique more robust as an alternative to HRV, but could also give it additional value for the extraction of information related to cardiovascular and autonomic health.

### Concluding remarks

PRV has been suggested as an alternative measurement to HRV, but several physiological and technical aspects may affect PRV measurements. Thanks to the widespread use of PPG sensors and their non-invasive and non-intrusive nature, PRV presents opportunity for more ubiquitous analysis of cardiovascular autonomic nervous activity, which could help understand how it relates to everyday activity even in healthy subjects. There is a substantial need for standardisation of the technique in order to better understand how it could effectively be used for the monitoring and diagnosis of cardiovascular and autonomic related diseases.

## 6. Respiratory monitoring

### Stephanie Baker^1^, Peter H Charlton^2^, Jesús Lázaro^3^

^1^College of Science and Engineering, James Cook University, Cairns, Queensland, Australia

^2^Department of Public Health and Primary Care, University of Cambridge, Cambridge, United Kingdom

^3^BSICoS Group, Aragon Institute of Engineering Research (I3A), IIS Aragon, University of Zaragoza, 50018 Zaragoza, Spain

### Status

**Figure 8.**
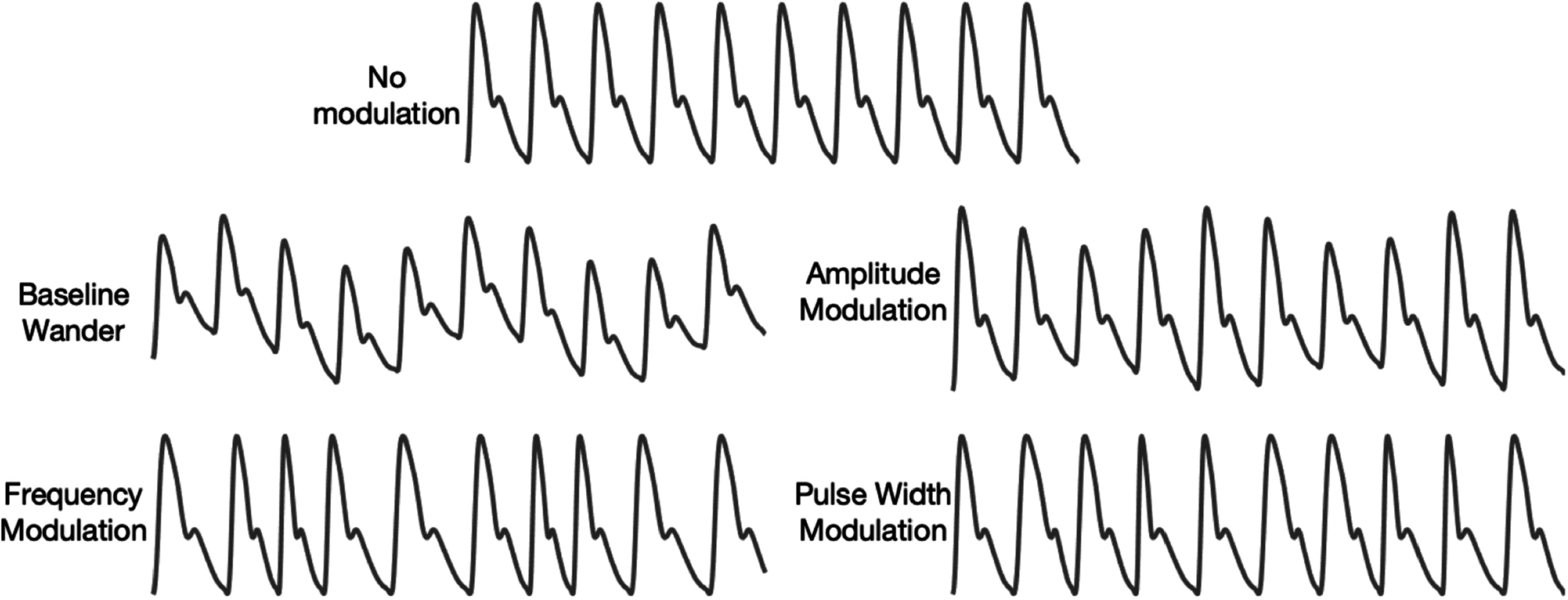
Effects of breathing on PPG signals. *Source: Adapted from* Charlton *et al* (2016) (CC BY 4.0).

Respiration is the exchange of oxygen and carbon dioxide between the body and the environment. It is essential for human life. Breathing subtly influences the PPG signal, providing opportunity to use the PPG for respiratory monitoring. The effects of breathing on the PPG include: frequency modulation induced by respiratory sinus arrhythmia; and baseline wander, amplitude and pulse width modulation (changes in the width of the systolic portion of the pulse wave) (Lázaro *et al* 2013) induced by intrathoracic pressure changes during respiration (see figure 8).

Much research has been conducted into estimating respiratory rate (RR) from PPG signals (Charlton *et al* 2018). The accuracy of these methods depends on the strength of respiratory modulations in the PPG signal, effectiveness in capturing these modulations, and robustness against noise. Several healthcare and consumer wearables now monitor RR from PPG signals (Charlton and Marozas 2022). Commercial fitness trackers have recently been used to measure nocturnal RR, with potential applications in identifying elevated RRs associated with COVID-19 (Natarajan *et al* 2021).

Nonetheless, significant issues with accuracy remain as PPG signals are particularly susceptible to noise. Noise can be non-physiological, such as motion artefact, or physiological, such as cardiovascular oscillations that are not related to respiration but overlap in the respiratory frequency band. Indeed, motion artefacts are to be expected when monitoring the PPG signal, particularly during daily life. Recent studies report that wearable PPG data is only of sufficient quality for pulse rate monitoring for 14%–56% of the time (Bonomi *et al* 2018, Eerikäinen *et al* 2018, Tarniceriu *et al* 2018, Bashar *et al* 2019). This is a significant limitation for respiratory monitoring, as obtaining respiratory information from PPG is more challenging than obtaining pulse rate.

As such, it is important to assess the reliability of respiratory information extracted from PPG signals. Several approaches have been proposed to assess PPG signal quality, including some specifically assessing the quality of respiratory signals derived from PPG modulations (Baker *et al* 2021). This strategy can be used to determine how much weight to give to RR estimates derived from each respiratory signal when using machine learning methods to estimate RR (Baker *et al* 2021).

Signal quality becomes increasingly important when measuring complex respiration parameters such as tidal volume and inspiration/expiration time. Several studies have found that the PPG can be used to monitor respiratory parameters and identify disordered breathing patterns associated with chronic obstructive pulmonary disease (COPD) (Davies *et al* 2022), interstitial lung disease (Yamazaki and Fujimoto 2021), sleep apnea (Kang *et al* 2020) and asthma (Prinable *et al* 2020). However, these studies were limited to controlled, at-rest settings with higher-resolution devices. Incorporation of signal quality assessment could enable the PPG to be used to monitor breathing using low-power wearable devices.

The use of machine learning algorithms also has the potential to improve PPG-based respiratory monitoring. Recent studies show that apnea and hypopnea events can be identified using long short-term memory (LSTM) networks to process segments of PPG signals, with minimal denoising required (Kang *et al* 2020). LSTMs have also been used to measure RR (Baker *et al* 2021). Other models, such as random forest and U-Net, have also been considered (Davies *et al* 2022) (Prinable *et al* 2020).

### Current and future challenges

One clear challenge in utilizing PPG data for respiratory health monitoring is ensuring the quality of said PPG data. Thus, a key challenge is to improve the robustness of PPG measurements against motion. This can be addressed by accurately quantifying the quality of PPG signals in the context of respiration. Whist methods have been developed to quantify PPG-derived respiratory signal quality for RR measurement, it is not yet clear whether these methods are suitable for measuring other respiratory parameters.

Where high-quality respiratory signals can be obtained, artificial intelligence (AI) has shown significant promise for accurately interpreting PPG signals in the context of respiratory health (Baker *et al* 2021). A key limitation of AI is that it is computationally expensive. In many applications, this is addressed by offloading data to cloud computing facilities for AI processing. However, offloading data to the cloud requires internet connectivity, which increases power consumption and latency, and is not reliably available in many rural areas. There are also substantial privacy concerns associated with storing sensitive health data in cloud facilities.

As previously mentioned, RR is not the only useful parameter related to breathing. Tidal volume is also clinically relevant. While there are many methods for deriving RR from the PPG, there are scant methods in the literature for deriving tidal volume from the PPG.

Continuous respiratory monitoring has a wide range of potential applications, particularly when combined with pulse rate and pulse rate variability measurements obtained from the PPG signal. These potential applications include stress level assessment (Momeni *et al* 2022), depression severity assessment (Zitouni *et al* 2022), and epileptic seizure detection (Forooghifar *et al* 2022). More direct applications include monitoring of chronic respiratory patients (e.g. patients diagnosed of COPD and/or asthma) for early detection of exacerbations, which is a life-saving application (see section 3 for further details) (Davies *et al* 2022, Taylor *et al* 2023). Sleep respiratory disorders such as sleep apnea deserve special mention, because there is less movement and thus fewer motion artefacts during sleep than during daytime activity. Further work towards these applications is required, since none are fully developed and used routinely in clinical practice.

Further work should establish how best to assess the performance of PPG-based respiratory monitoring techniques (Charlton *et al* ). Firstly, the growing number of publicly available PPG datasets provides opportunity to assess performance across multiple datasets to investigate generalisability across different settings and devices. It is particularly important to assess performance in daily life, since most previous work has been conducted in healthcare settings or controlled conditions. Secondly, the statistical methods used should be tailored to the intended application. To date, assessments of RR algorithms have focused on performance at a single time-point. In the future, studies could also assess the ability of algorithms to track changes in parameters over time, using similar approaches to those in section 21.

### Advances in science and technology to meet challenges

Ensuring and improving the quality of PPG signals is critical to enable accurate respiratory health monitoring using these sensors. Metrics for measuring respiratory signal quality must continue to be developed and validated in contexts beyond RR measurement. This will enable better evaluation of future techniques for improving the robustness of PPG signals against noise. Denoising of PPG and PPG-derived signals remains an active challenge in this domain, and future research should seek to address this using novel approaches. Potential approaches include: (i) using advanced AI models, such as autoencoders and transformers, to reconstruct clean signals from noisy ones; and (ii) using pulse decomposition analysis to reconstruct denoised PPG signals (based on physiological models, e.g. a sum of Gaussians). Alternatively, it may be possible to enhance signal quality by positioning PPG sensors in regions with higher vein density (Nilsson *et al* 2003), which may be supported through the development of flexible and adhesive PPG sensing circuits. The optimal method remains unknown and may depend on the specific application.

To overcome the challenges associated with cloud-based AI, future research should focus on strategies for implementing AI on edge devices. This primarily involves developing AI architectures with lower computational complexity, without compromising on performance compared to existing methods. This can be achieved by systematically identifying features that are critical to accurate prediction or measurement. An emerging strategy is the use of explainability tools, such as shapley additive explanations (SHAP) or local interpretable model-agnostic explanations (LIME), to identify which features contributed most strongly to a model’s output. The model can then be revised to use only the few most important features. In terms of lightweight edge AI architectures, suitable candidates include random forests, shallow convolutional neural networks (CNNs), and LSTMs.

With respect to tidal volume estimation, the amplitude of the respiratory modulations that serve as a basis for estimating RR from PPG could also serve as a basis for tracking tidal volume changes. Furthermore, tidal volume may be estimated in absolute terms after a calibration process. Estimating tidal volume in addition to RR would provide a more complete view of breathing, and that would be very interesting in several applications. In fact, all the mentioned applications will potentially benefit from new features based on tidal volume information, since their relation with respiration is actually not limited to RR, but to ventilation.

### Concluding remarks

Overall, the use of photoplethysmography for respiratory monitoring is an active field of research. Recent studies have advanced the field through the development of signal quality assessment metrics, use of advanced signal processing techniques, and implementation of machine learning. Nonetheless, significant challenges and opportunities remain for current and future researchers in this exciting field of healthcare. Existing techniques could be improved to enhance the feasibility of using wearables for continuous PPG-derived respiratory monitoring, and further work is required to investigate whether respiratory monitoring can be performed reliably in activities of daily life. Novel techniques are being developed to assess additional respiratory metrics from the PPG such as tidal volume and inhalation/exhalation times, which when combined with RR could provide more holistic views of breathing and ventilation. Additionally, few studies have explored the application of this technology for the diagnosis, monitoring and management of respiratory health conditions; thus, this remains a key direction for future research.

## Acknowledgments

PHC acknowledges funding from the British Heart Foundation (FS/20/20/34626) and an EPSRC Impact Acceleration Award. JL acknowloedges funding from CIBER in Bioengineering, Biomaterials & Nanomedicine through Instituto de Salud Carlos III and by Gobierno de Aragon (Reference Group BSICoS T39-20R) cofunded by the Fondo Europeo de Desarrollo Regional (FEDER) 2014–2020 ‘Building Europe from Aragon’.

## 7. Pulse wave analysis

### Vaidotas Marozas^1,2^ and Eduardo Gil^3,4^

^1^Department of Electronics Engineering, Kaunas University of Technology, 44249 Kaunas, Lithuania

^2^Biomedical Engineering Institute, Kaunas University of Technology, 44249 Kaunas, Lithuania

^3^Biomedical Signal Interpretation and Computational Simulation (BSICoS) group at the Aragon Institute of Engineering Research (I3A), IIS Aragon, University of Zaragoza, Spain

^4^CIBER-BBN, Instituto de Salud Carlos III, Madrid, Spain

### Status

Artery pulse palpation was used by Chinese, Indian, Greek, and Roman physicians. Galen (129–210 AD) described 27 types of pulses and their meaning (Parker 2009), but only Herzman, in 1937, introduced an instrument capable of objectively recording pulse waves and comfortable enough to be ‘brought to the subject, not the subject to the instrument’ Dillon and Hertzman (1941). The instrument recorded pulses of blood volume and was named photoelectric plethysmography (PPG). Gradually, the main differences among blood volume, pressure, and flow pulse waves were understood. Striking differences of pulse waveforms from the different body sites and due to different health conditions were observed quite early Dillon and Hertzman (1941). Peripheral pulse waves (PPWs) were investigated mainly from the fingers, toes, and earlobes but can also be obtained from the forehead, nose, esophagus, chest, arm, and wrist (Park *et al* 2022b). PPWs change due to a person’s age, elevated blood pressure, arteriosclerosis, fever, chronic nephritis, pain, mental stress, emotions, and fitness. These changes sparked imagination about the potential clinical and ambulatory applications of pulse wave analysis (PWA) (Charlton *et al* 2022).

The morphology of PPWs is a significant source of diagnostic information since the PPW is determined by the interaction of the heart and blood vessels, but also modulated by respiration and autonomic nervous system activities. Therefore, pulse wave analysis (PWA) can be considered a non-invasive ‘window’ to the main physiological processes of the body. Although PWA is also performed on blood pressure signals (Mynard *et al* 2020), the appeal of continuous and comfortable health monitoring during daily life provided by personal PPG devices has extended PWA to the PPG signal.

The shape of the PPW is influenced not only by the incident wave transmitted through the arteries from the heart but also by reflected and rereflected waves from a range of arterial sites (Baruch *et al* 2014). Potentially, the amplitude and timing of these waves provide useful diagnostic information about the functional state of physiological systems. The best-known indicators obtained through PWA are arterial stiffness, reflection, augmentation, and aging indexes (Charlton *et al* 2022), but many more are proposed. Long-term monitoring of dynamic variations of PWA indexes combined with anomaly detection and interpretable machine learning algorithms could potentially provide diagnostic value, and also insight into causal mechanisms involved in the development of chronic diseases. Although PWA is a promising tool for health assessment, it is not a well-established methodology, with some challenges to be solved.

### Current and future challenges

**Figure 9.**
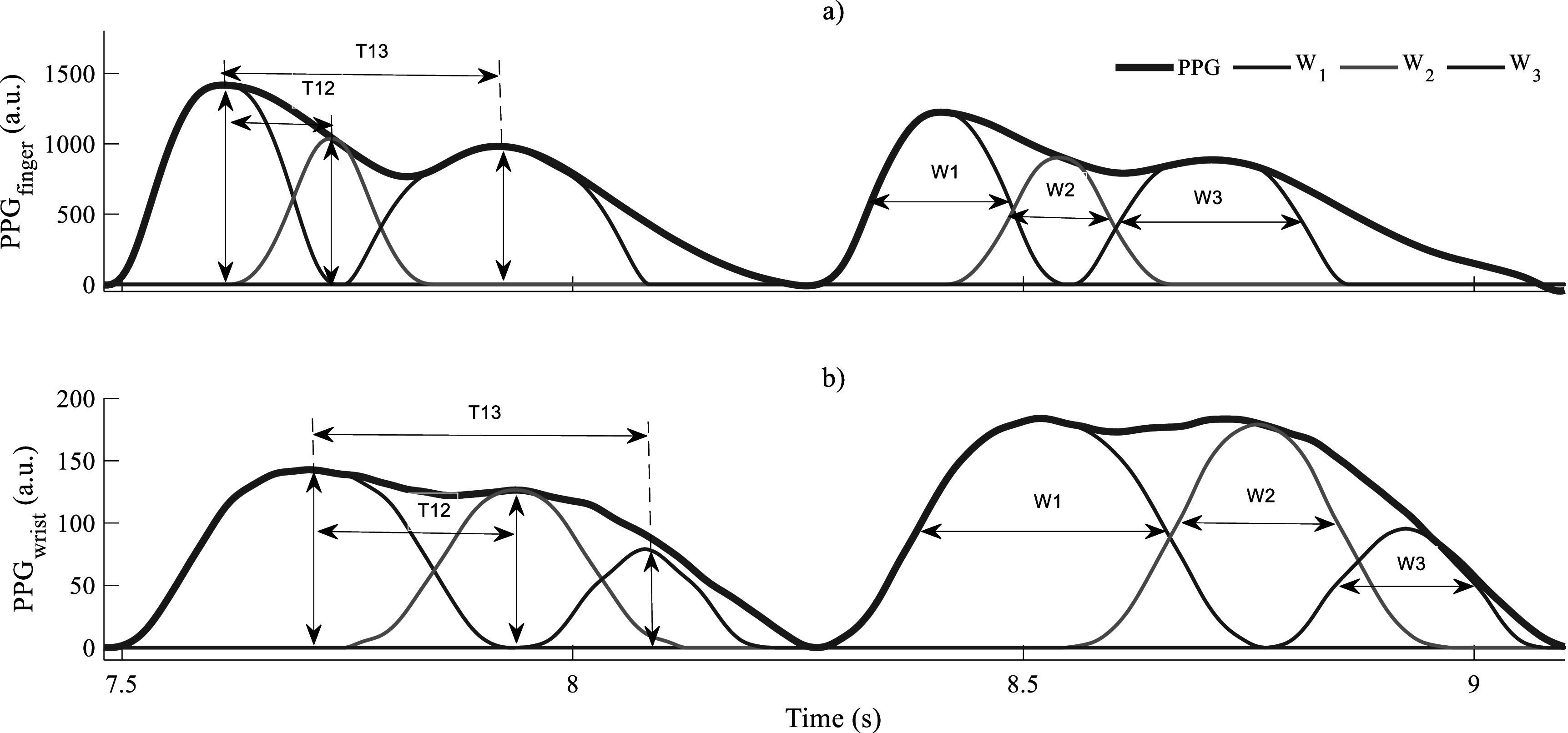
An illustration presenting significant differences in the PWD results of synchronized PPG signals from the finger (a) and wrist (b). Here: W1-W3 and T12, T13 are features extracted from decomposed waves W_1_–W_3_ (see Kontaxis *et al* (2021) for more details about the PWD method).

Progress in PWA research depends partly on developments in digital signal analysis algorithms. Time domain-based algorithms using joint analysis of original, and two or three first derivatives of the PPG waveforms were offered first (Charlton *et al* 2022). All popular PWA indicators are calculated in the time domain. Surprisingly, very few studies used frequency, time-frequency, time-scale domains, blind source separation (such as independent component analysis) or nonlinear analysis tools for PWA of the PPG signal. Model-based (mixture of Gaussian and/or lognormal functions) and data-driven (slope reflection-based) pulse-wave decomposition (PWD) algorithms (Kontaxis *et al* 2021) have received more attention (figure 9). Reliable tracking the time course of the extracted PWA or PWD indicators is an issue related to both the algorithm and the quality of the data.

**Figure 10.**
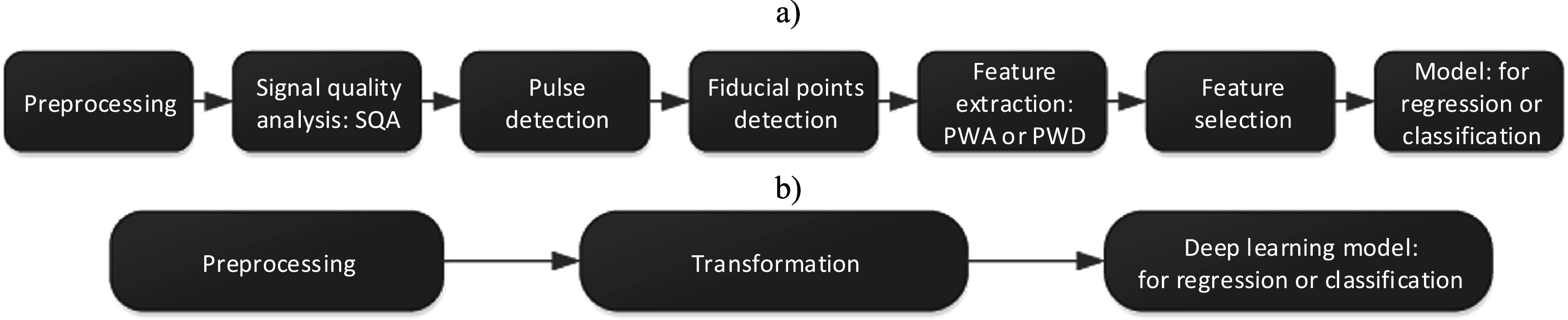
Conventional (a) and modern (b) pipelines of PWA.

The pre-processing of the PPG signal is an essential step in PWA as it is carried out to remove motion artifacts, distortions, and noises while preserving the pulse wave morphology (figure 10(a)). Signal quality assessment (SQA) is an important step in PWA. It saves computational resources by excluding low-quality pulses whilst retaining diagnostic quality pulses for analysis. During the daytime, only a small number of pulses can be expected to be of excellent quality and therefore suitable for PWA analysis (Moscato *et al* 2022).

Many factors influence the morphology of pulse waves. When measuring one factor, all the others should be considered confounders, e.g. the pulse amplitude can change due to changes in blood pressure, but also due to changes in the contact force of the PPG sensor (May *et al* 2021) or hand position relative to heart level (Hickey *et al* 2016). Pulse morphology influencing factors can be classified as static (related to the measurement method and context, e.g. reflection or transmission, body site, skin color, sex, height, arm length, BMI, etc) and dynamic (heart activity, state of blood vessels, subject’s posture, movement artifacts, etc). Therefore, one of the most important challenges in a successful PPG-based PWA is a comprehensive understanding of factors influencing PPG morphology (Proença *et al* (2019)).

A significant challenge in PWD is the biophysical interpretation of extracted indicators. The link among the decomposed waves (shown in figure 9) and their physiological meaning is not obvious. Therefore, the interpretation of PWD analysis should be very careful.

### Advances in science and technology to meet challenges

Advances in sensor technology open new avenues for new PWA and PWD applications. The study (Yokota *et al* 2020) investigated an organic photodiode-based imager for pulse-wave mapping. The conformable, soft contact imager with a resolution of 508 pixels per inch and 41 frames per second allows the best measurement location to be selected electronically, or spatial averaging to be implemented to improve SNR. Non-contact imagers, i.e. video and smartphone cameras, offer the means for PWA unobtrusive monitoring, which could be used for patients with severe burns and in routine clinical or ambulatory monitoring applications, e.g. haemodialysis or sleep. However, improved reliability is needed in uncontrolled environments. The sensor fusion of PPG-based PWA with other modalities such as ultrasound or magnetic resonance imaging could potentially provide a synergistic effect in estimating local artery compliance and characterization of arteriosclerosis even for deep arteries. A prerequisite for sensor fusion is the strict synchronization of different modalities.

Signal processing methods have provided many spatio-temporal features of PPG that have been analysed in great detail. Advances in signal processing should aim to find the physiological significance of these indicators or a combination of them to be useful in routine clinical or ambulatory applications. For example, PWD methods could be supported with personalized constraints and thresholds based on physiology data to guarantee the interpretation of each wave among the different pulse morphologies.

Machine learning methods could be useful for feature extraction and selection to choose the most relevant indicators for a particular application. Taking this philosophy to the extreme, using raw signals as input to the machine learning model avoids the estimation of features (figure 10(b)). Although it could be useful for the final application, no knowledge about the physiological significance of the different characteristics of pulse morphology is obtained. Interpretability methods could be employed to reveal parts of the original or transformed PPG signal that are important for making regression or classification decisions in specific applications. One example of such a method is Gradient-weighted Class Activation Mapping (Grad-CAM). Finally, machine learning models must be trained and tested with heterogeneous representative databases of PPG signals, and the uncertainty of the model must be quantified.

Fast advances in bioelectronics and signal processing, biophysical modeling and interpretation, machine learning, and deep learning methods make PWA attractive for the next generation of consumer wearable devices.

### Concluding remarks

In summary, there are a few different methods for performing PWA in PPG signals, but only a small number of PWA applications are considered in medical and consumer wearable devices. To increase the acceptance of PWA technology, substantial research and development is still needed to improve the efficiency of PWA in the extraction of biophysically relevant parameters that are invariant to confounding factors. Advances in optoelectronics and integration with other types of sensors will increase the quality of PPG signals, and biophysics-guided machine learning will provide interpretable and clinically useful PWA-based health indicators. In the next decade, we expect to see wearable devices capable of unobtrusively monitoring long-term trends of hemodynamic variables and providing timely recommendations before dangerous changes in the health of the users.

## Acknowledgments

This work was supported by the European Regional Development Fund with the ‘Ministerio de Ciencia e Innovación’ of Spain under project PID2021-126734OB-C21 and with the Research Council of Lithuania (LMTLT) under Projects 01.2.2-LMT-K-718-01-0030 and 01.2.2-LMT-K-718-03-0027, the European COST ACTION ‘Network for Research in Vascular Ageing’ CA18216 supported by COST (European Cooperation in Science and Technology): www.cost.eu.

## 8. Development of wearable pulse oximeters

### Toshiyo Tamura

Waseda University

### Status

Oximetry is the measurement of haemoglobin oxygen saturation in blood or tissue and depends on the Lambert–Beer relationship between light transmission and optical density. Pulse oximeters are non-invasive, compact, and affordable devices that have been used for decades to quantify oxygen saturation. They allow early detection of hypoxaemia, enhance patient safety, decrease the anaesthesia mortality rate, and reduce caregiver workload (Tamura 2019). During the coronavirus disease 2019 (COVID-19) pandemic, home use of pulse oximeters increased, helping clinicians to identify cases requiring medical intervention. Pulse oximetry is currently being integrated into wearables. While these enable oxygen-saturation monitoring in daily life with several potential applications, many challenges remain to ensure accurate wearable pulse oximeters.

**Figure 11.**
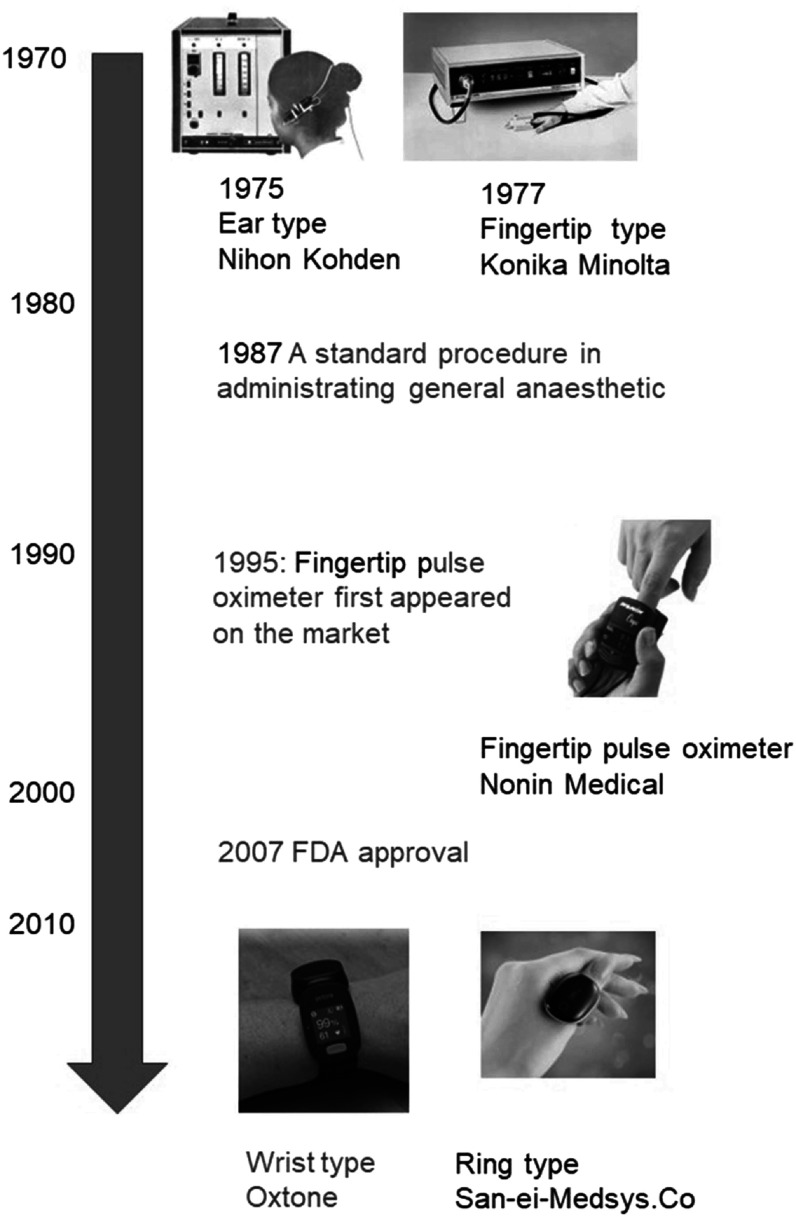
History of the pulse oximeter. The photographs are reproduced with permission from Nihon Koden (Tokyo, Japan), Konika Minolta (Tokyo, Japan), Nonin Medical (Plymouth, USA), Oxitone Medical (Kfar Saba, Israel) and San-ei Medsys. Kyoto, Japan) 2021.

In 1935, Matthes developed the first oxygen-saturation meter. It used a two-wavelength light source with red and green filters, which were later changed to red and infrared (IR) filters. In the 1940s, Millikan, a British scientist, used a dual light source to create the first aviation ear oxygen meter. In 1964, Hewlett Packard created the first ear oximeter using light of eight wavelengths; these were used primarily in sleep laboratories and pulmonary clinics but were expensive, cumbersome, and large. In 1972, Takuo Aoyagi, a Japanese bioengineer at Nihon Kohden, developed a pulse oximeter based on pulsatile variations in the optical density of tissues in the red and IR wavelengths to quantify arterial oxygen saturation based on the ratio of red to IR light absorption in blood, with no need for calibration. Susumu Nakajima (a surgeon) and his associates first tested the device in patients in 1975. Dr Aoyagi obtained a Japanese patent. Minolta obtained a United States patent based on the same concept. Nihon Kohden introduced an ear oximeter in 1975, and Minolta began marketing a fingertip area oximeter in 1977. The history of the pulse oximeter is shown in figure 11.

In the United States, Ohmeda Biox and Nellcor introduced the pulse oximeter. Initially, it was applied for respiratory care, but its use later expanded to operating rooms. Since then, other manufacturers have entered the market and pulse oximeter technology has improved significantly.

Pulse oximetry became part of the general anaesthetic procedure in the United States in 1987, and quickly spread to emergency rooms, recovery rooms, neonatal units, and intensive-care units. The first fingertip pulse oximeter appeared on the market in 1995. The Food and Drug Administration (FDA) published a notice in the Federal Register (Vol. 72, No. 138; July 19, 2007) entitled ‘Draft Guidance for Industry and Food and Drug Administration Staff; Pulse Oximeters Premarket Notification Submissions [510(k)s]’. Shortly thereafter, FDA-approved pulse oximeters became available.

Handheld pulse oximeters with small probes, wrist-worn devices with finger probes or embedded light-emitting diode-photodetector (LED-PD) sensors, fingertip devices, and ring oximeters are all available. Instruments used clinically include wrist-worn devices such as WristOx (Nonin Medical, Plymouth, MN, USA), Pulsox 500i (Konica Minolta, Tokyo Japan), and Checkme O2 (Viatom Technology, Shenzhen, China); wireless telemetry devices with pulse oximetry; and an embedded pulse oximeter (Checkme; San-ei Medsys, Kyoto, Japan). Recently, pulse oximeters have become affordable for both in-hospital and home use. Typical wrist-pulse oximeters include the Oxitone 1000 M (Oxitone Medical, Kfar Saba, Israel) and a wrist monitor (Biobeat Wrist watch; Bio-beat Technologies, Petah Tikva, Israel), which have been approved by the FDA and used in hospitals.

Non-FDA-approved devices include the Apple Watch, Fitbit, and Galaxy Watch, which have personal health applications. Differences between approved and non-approved devices are discussed in the next section. More recently, ring-pulse oximeters have been released, such as the CIRCUL (BodiMetrics, Manhattan Beach, CA, USA), RingO2 (Viatom, Shenzhen, China), and Checkme Ring (San-ei Medsys) (Jung *et al* 2022, Santos *et al* 2022, Zhao *et al* 2022). A patch oximeter is also under development.

### Current and future challenges

Pulse oximeters are non-invasive and simple to use, and enable early diagnosis of pulmonary dysfunction. During the COVID-19 pandemic, pulse oximeters attracted raised attention, but several questions also arose regarding their use. Many factors can affect photoplethysmography (PPG) signals, including the light wavelength, measurement site, contact force, motion artifacts, skin colour, henna pigmentation, nail varnish, and ambient light intensity and temperature.

Pulse oximeters may over- or underestimate oxygen saturation. Their accuracy is affected by skin pigmentation, the fit of the device, peripheral blood flow, nail coatings, tattoos and dyes, and device maintenance and cleaning. Currently, skin pigmentation is a major research topic. Pulse oximeters may overestimate oxygen saturation in individuals with relatively dark skin, potentially resulting in hypoxia not being treated (Sjoding *et al* 2020). Overestimation by pulse oximeters has been reported in cases of low perfusion (Bickler *et al* 2005), and results can be inaccurate depending on the level of skin pigmentation (Cabanas *et al* 2022). These inaccuracies are greater at lower levels of oxygenation. Oxygen saturation measured by pulse oximetry is 3%–4% higher than that determined by the gold standard, i.e. arterial blood gas analysis (Crooks *et al* 2022, Fawzy *et al* 2022, Gottlieb *et al* 2022).

The degree of inaccuracy varies among devices, and the evidence is insufficient for the Therapeutic Goods Administration to make recommendations. Most pulse oximeters are approved by medical regulatory authorities, but some are available over the counter (Lipnick *et al* 2016). Over-the-counter pulse oximeters can be less accurate than devices with regulatory approval (Lipnick *et al* 2016; Therapeutic Goods Administration 2022). Overreliance on pulse oximeters could lead to suboptimal management of patients with abnormal oxygen levels; the risk is higher in people with darker skin (Sjoding *et al* 2020).

Sale of over-the-counter devices is not regulated; they are sold directly to consumers in stores or online as general wellness products or for sports or aviation use, and were not designed for medical use. Medical-use pulse oximeters undergo clinical testing to confirm their accuracy, are reviewed by medical-device authorities, and are available only with a prescription. They are typically used in hospitals and doctors’ offices, although they are sometimes prescribed for home use.

Research has focused on the use of multi-wavelength oximeters and algorithms to prevent motion artifacts. Green light is used to confirm heart rate regularity and adjust the pulse oximetry signal. SpO_2_ values are highly reliable (Ray *et al* 2023).

The contact force between tissue and the sensor affects the accuracy of SpO_2_ values obtained using transmission and reflectance probes. Insufficient contact pressure can result in a weak PPG signal, while excessive pressure blocks the circulation and deforms the PPG. The range of contact force that generates optimal PPG signals with salient pulsatile components has been revealed recently; forces of 5–15 kPa were associated with error rates <2%. Combined probes could improve the reliability of reflectance oximeters and help optimize the fit of wearable devices.

Reflectance pulse oximeters have been used in both hospital and home settings. Mendelson *et al* (1988) examined a reflectance probe in 1988. The light intensity of the IR LED was low and the measurement site was the forehead. Regression analysis revealed a high correlation and relatively small standard error of the estimates. Although light scattering influences accuracy, in most studies the accuracy of SpO_2_ values obtained using reflectance pulse oximeters were similar to those obtained using a transmittance probe worn on the finger. The penetration depth of transmitted light affects signal reception. Before the development of high-intensity LEDs, only IR LEDs could be used, which were suitable only for the forehead. However, since then, high-intensity LEDs have enabled the development of wrist oximeters. For clinical use, transmittance probes are preferrable, although forehead reflectance probes are used in some cases.

### Advances in science and technology to meet challenges

As mentioned above, the accuracy and validity of pulse oximeters have attracted considerable attention, especially in terms of the influence of skin pigmentation in the clinical setting, the optimal wavelength, the development of patch sensors, and regulatory issues.

Pulse oximetry errors are likely amplified by low perfusion and motion, among other factors. Although the greater absorbance of red light by melanin seen in relatively dark skin may result in overestimation of oxygen saturation, further studies are necessary to quantify this effect, determine whether dark skin is correlated with other factors that affect oximeter performance, and evaluate the impact of dark skin in the presence of other factors that affect signal quality and processing (e.g. low perfusion or use of a low-quality oximeter) (Okunlola *et al* 2022). Dark skin is a risk factor for hypoxia going undetected by pulse oximetry. Clinicians should adjust treatments accordingly and consider inspecting the pulse oximeters used in their institutions.

It is difficult to determine weights for different skin colours when using pulse oximetry for diagnosis. The utility of the Fitzpatrick skin-type test, which is used to assess constitutive skin colour, is limited; a large study involving subjects with both light and dark skin is required. There has been little interest in solving the inherent problems of pulse oximeters, despite their clinical relevance. Research to address these problems is important, and clinicians should engage with engineers, regulators, and other stakeholders to this end.

Multi-wavelength oximeters have been developed to measure methaemoglobin and carboxyhaemoglobin. However, red and IR illumination produce less reliable signals during movement. Green might serve as an alternative, as it can provide signals that are more resilient to motion artefacts.

The measurement site should also be considered, and a low signal-to-noise ratio is required for the perfusion index. Objective methods should be used to quantify skin pigment at the site of oximeter measurement (e.g. the ear or finger), and dorsal and ventral measurements should be obtained.

Regarding standards and regulations for oximeters, the FDA 510k accuracy requirement is <3% for transmittance sensors and <3.5% for reflectance sensors; the ISO requirement for the latter is <4%. Accuracy limitations should be considered when using pulse oximetry to aid diagnosis and treatment decision-making. Standardisation of test protocols is required to elucidate pulse-oximetry errors in patients with dark skin. This will enable performance evaluation data to be shared.

A patch oximeter with an adhesive sensor is under development. The advantages of patch sensors include flexibility due to the organic material used in the LED and ultralow power requirements. Disposable probes are important in the COVID-19 era (Taylor-Williams *et al* 2022).

### Concluding remarks

Pulse oximetry enables monitoring of respiratory function during anaesthesia, but the results are affected by skin pigmentation. Objective measurements of skin pigmentation should be performed (e.g. colourimetry) rather than relying on subjective descriptions or race/ethnicity. To improve oximeter accuracy, standardisation is required. Pulse-oximetry errors in patients with dark skin must be quantified in large studies involving subjects with various levels of skin pigmentation. Testing protocols and guidelines are also needed, and standards of accuracy should be established through the collaboration of regulatory authorities, research institutes, medical professionals, and manufacturers.

## Acknowledgments

This work was supported in part by grants-in-aid from the Japanese Ministry of Education, Culture, Sports, Science and Technology, Scientific Research (C) (Kakenhi) (#21K12760) and the Japan Agency for Medical Research and Development (JP22dk0310111).

## 9. PPG signal quality: not a black and white matter

### Cheng Ding^1^, Tania Pereira^2,3^ and Xiao Hu^4,5,6^

^1^Department of Biomedical Engineering, Georgia Institute of Technology & Emory University

^2^INESC TEC—Institute for Systems and Computer Engineering, Technology and Science, Portugal

^3^Faculty of Engineering, University of Porto, Portugal

^4^Nell Hodgson Woodruff School of Nursing

^5^Department of Biomedical Informatics, School of Medicine

^6^Department of Computer Sciences, College of Arts and Sciences, Emory University

### Status

**Figure 12.**
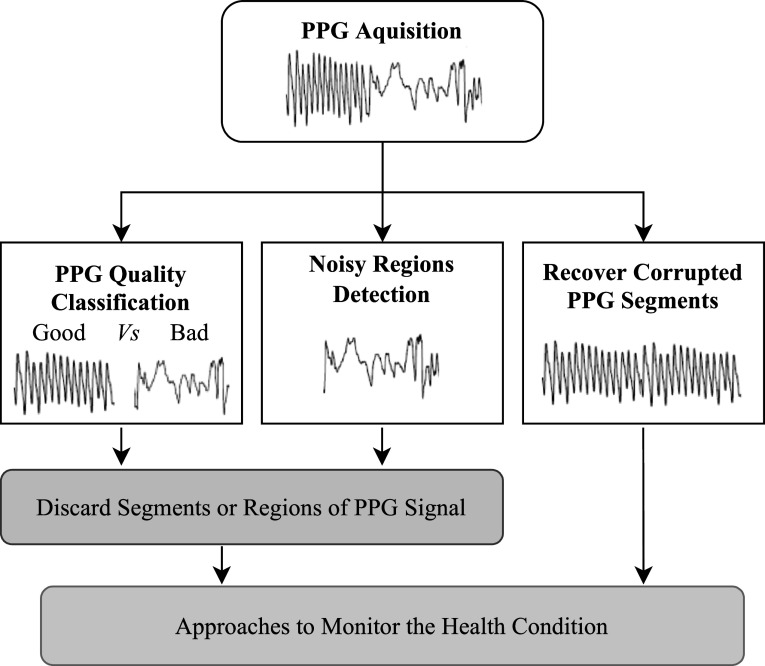
Representation of the three main approaches proposed to handle imperfect PPG signals.

The photoplethysmogram (PPG) is a non-invasive signal which can be used to detect physiological changes in hemodynamic and cardiac functions. However, its recording can be corrupted by subject movement, ambient light, and inappropriate sensor placement resulting in imperfect signals. Indeed, as detailed in section 6, studies have reported that between 44% and 86% of PPG signals collected are not of sufficient quality for pulse rate monitoring. PPG signal quality has been found to be substantially influenced by physical activity and health status (Moscato *et al* 2022). Therefore, optimal handling of imperfect PPG signals is of ultimate importance to realize the full potential of this ubiquitously available technology. The field has undoubtedly recognized the importance of PPG signal quality as evidenced by numerous studies aimed at developing approaches of (1) classifying PPG signal quality (Pereira *et al* 2019, 2020 *,* Mohagheghian *et al* 2022); (2) recognizing artifactual regions in a PPG signal (Guo *et al* 2021); (3) recovering corrupted PPG signals (Mishra and Nirala 2020), these three main approaches are represented in figure 12. Many studies classify PPG signal quality with a goal to exclude periods of poor quality signal from downstream tasks such as arrhythmia detection (Orphanidou *et al* 2015, Elgendi 2016). However, this conventional way of treating PPG signal quality as a black and white matter is not optimal as will be shown in the next section. Delineating artifactual regions in a PPG signal and recovering true signals from corrupted ones are two more challenging tasks and the development of novel approaches to accomplish these tasks has started to appear in the literature.

### Challenges

The challenge to incorporate signal quality as an integral part of the pipeline to accomplish a classification task can be illustrated mathematically. In this pipeline, the classification task is only performed on PPG signals whose quality is greater than a threshold. Let ${N}_{0}^{\mathrm{TP}},{N}_{0}^{\mathrm{FP}},{N}_{0}^{\mathrm{TN}},$ and ${N}_{0}^{\mathrm{FN}}$ denote the number of true positives, false positives, true negatives, and false negatives in the test dataset of only good quality signals and ${N}_{1}^{\mathrm{TP}},{N}_{1}^{\mathrm{FP}},{N}_{1}^{\mathrm{TN}},$ and ${N}_{1}^{\mathrm{FN}},$ the corresponding metrics calculated based on the excluded poor-quality PPG signals. Using these notations, the sensitivity for the classification task is ${N}_{0}^{\mathrm{TP}}/\left({N}_{0}^{\mathrm{TP}}+{N}_{0}^{\mathrm{FN}}+{N}_{1}^{\mathrm{TP}}+{N}_{1}^{\mathrm{FN}}\right)$ and the positive predictive value (PPV) is ${N}_{0}^{\mathrm{TP}}/\left({N}_{0}^{\mathrm{TP}}+{N}_{0}^{\mathrm{FP}}\right).$ However, if we also perform classification task on excluded samples to improve the sensitivity to $\left({N}_{0}^{\mathrm{TP}}+{N}_{1}^{\mathrm{TP}}\right)/\left({N}_{0}^{\mathrm{TP}}+{N}_{0}^{\mathrm{FN}}+{N}_{1}^{\mathrm{TP}}+{N}_{1}^{\mathrm{FN}}\right),$ the corresponding PPV becomes $\left({N}_{0}^{\mathrm{TP}}+{N}_{1}^{\mathrm{TP}}\right)/\left({N}_{0}^{\mathrm{TP}}+{N}_{0}^{\mathrm{FP}}+{N}_{1}^{\mathrm{TP}}+{N}_{1}^{\mathrm{FP}}\right).$ By simple algebra, we show that PPV after including some imperfect PPG signals will be non-inferior to that based on only clean PPG signal when ${N}_{1}^{\mathrm{TP}}/{N}_{1}^{\mathrm{FP}}\geqslant {N}_{0}^{\mathrm{TP}}/{N}_{0}^{\mathrm{FP}}.$ Therefore, one needs to carefully select the signal quality threshold to achieve maximal PPV without compromising sensitivity. Unfortunately, we have yet to identify any published studies that exploit this insight.

Moreover, the morphological characteristic varies when identifying the PPG signal quality under different conditions. First, the shape and amplitude of the PPG signal differ between modes. For example, for transmission-mode photoplethysmography, such as finger or in-ear photoplethysmography, the photodetector detects the light transmitted through the medium, whereas for reflectance-mode photoplethysmography from wrist-worn watches, the reflection time of light from the blood vessels is recorded. Therefore, it impedes the generalizability of PPG signal quality assessment. Second, the interpatient variability also influences the morphology of PPG signals. The time-varying dynamics as well as different temporal and physical conditions of different patients change the shape, amplitude, and intervals of the signals, which also makes proposing a generalizable strategy to identify the PPG quality difficult. Third, because the PPG is often used in tasks other than classification (e.g. estimating heart rate or blood pressure), the strategy designed for classification tasks to determine an optimal signal quality threshold to exclude an imperfect PPG signal is difficult to adopt.

### Advances in science and technology to meet challenges

**Figure 13.**
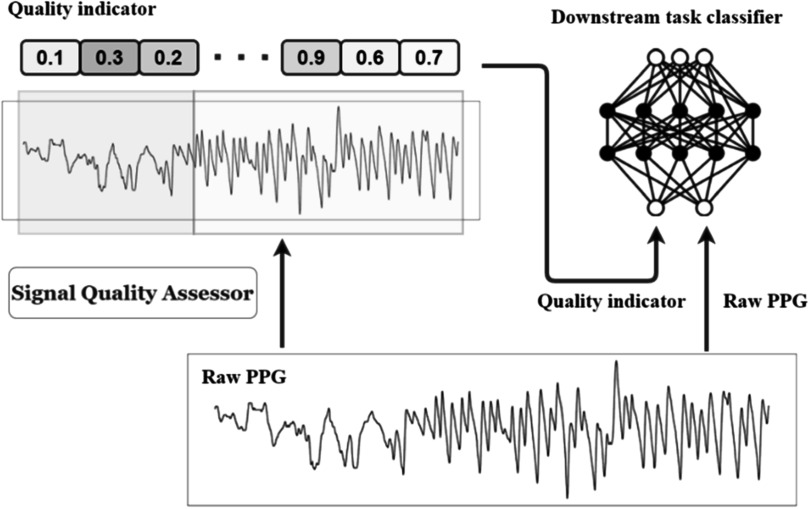
Integrating signal quality assessment with downstream tasks.

As discussed above, a more nuanced treatment of PPG signal quality as an integral part of a PPG signal processing pipeline is needed to ensure optimal performance of the ultimate task. Here, we propose a potential unified approach to achieve this goal. This approach is based on the premise that the ultimate task will benefit from maximizing the use of clean samples in a PPG signal segment while minimizing the impact of artifactual samples. For example, a detection algorithm of atrial fibrillation (AF) should still be able to confidently predict AF even from a few seconds of clean PPG signal samples that are embedded in an otherwise vastly noisy signal. In terms of algorithm design, one would require weighting differently the contribution of signal samples that have different signal quality to the downstream task. This idea requires one to integrate signal quality assessment as an integral part of the downstream task. This contrasts with treating signal quality assessment as a separate pre-processing task to exclude imperfect PPG signals. Figure 13 illustrates one example of this design where a signal quality assessor is used to quantify a signal quality for each consecutive sub-segment of the signal, which is then used in the downstream model in such a way that subsegments with higher signal quality will be more ‘attended’ by the model. To achieve an accurate quality assessment of subsegments, we can treat artifact detection as a 1D segmentation problem (Guo *et al* 2021), where we aim to segment artifacts from non-artifacts. Another method is to use post-hoc explanation techniques, such as class activation map (Zhou *et al* 2016) and layer-wise relevance propagation (Binder *et al* 2016), on a binary quality assessment classifier to generate the importance of each signal data point. Subsegments with more contribution to the good-quality class will be more ‘attended’ by the model.

### Concluding remarks

Optimal handling of PPG signals of imperfect quality is critical to downstream tasks. Mathematical derivation shows that simply excluding signals whose quality is below a threshold will underperform if the threshold is not chosen at an optimal value. We propose a generic algorithm framework to address this challenge through a more nuanced treatment of PPG signal quality as an integral part of a complete PPG signal processing pipeline.

## Acknowledgments

We greatly appreciate domain expertise in cardiology and neurology from our collaborators including Drs Randall Lee, Duc Do, and Karl Meisel.

## 10. Motion artifacts in wearable photoplethysmography

### Chungkeun Lee^1^ and Hangsik Shin^2^

^1^Digital Health Devices Division, Medical Device Evaluation Department, National Institute of Food and Drug Safety Evaluation, Ministry of Food and Drug Safety, Cheongju, Republic of Korea

^2^Department of Digital Medicine, Asan Medical Center, University of Ulsan College of Medicine, Seoul, Republic of Korea

### Status

Photoplethysmography (PPG) is extensively employed in the latest watch and ring-type wearable electronic devices, because it can offer rich cardiovascular information (Kyriacou and Allen 2021). PPG obtained by nonprofessional users in daily life may contain fluctuations due to several external factors; thus, it is difficult to guarantee the results’ reliability. One of the most critical factors that deteriorate the reliability of wearable PPG measurements is motion artifact (MA). In most cases, the pattern of MA is unpredictable, and it occurs very frequently in everyday life, greatly reducing the signal’s reliability. This can lead to errors in interpreting the results, and potential misdiagnosis.

**Figure 14.**
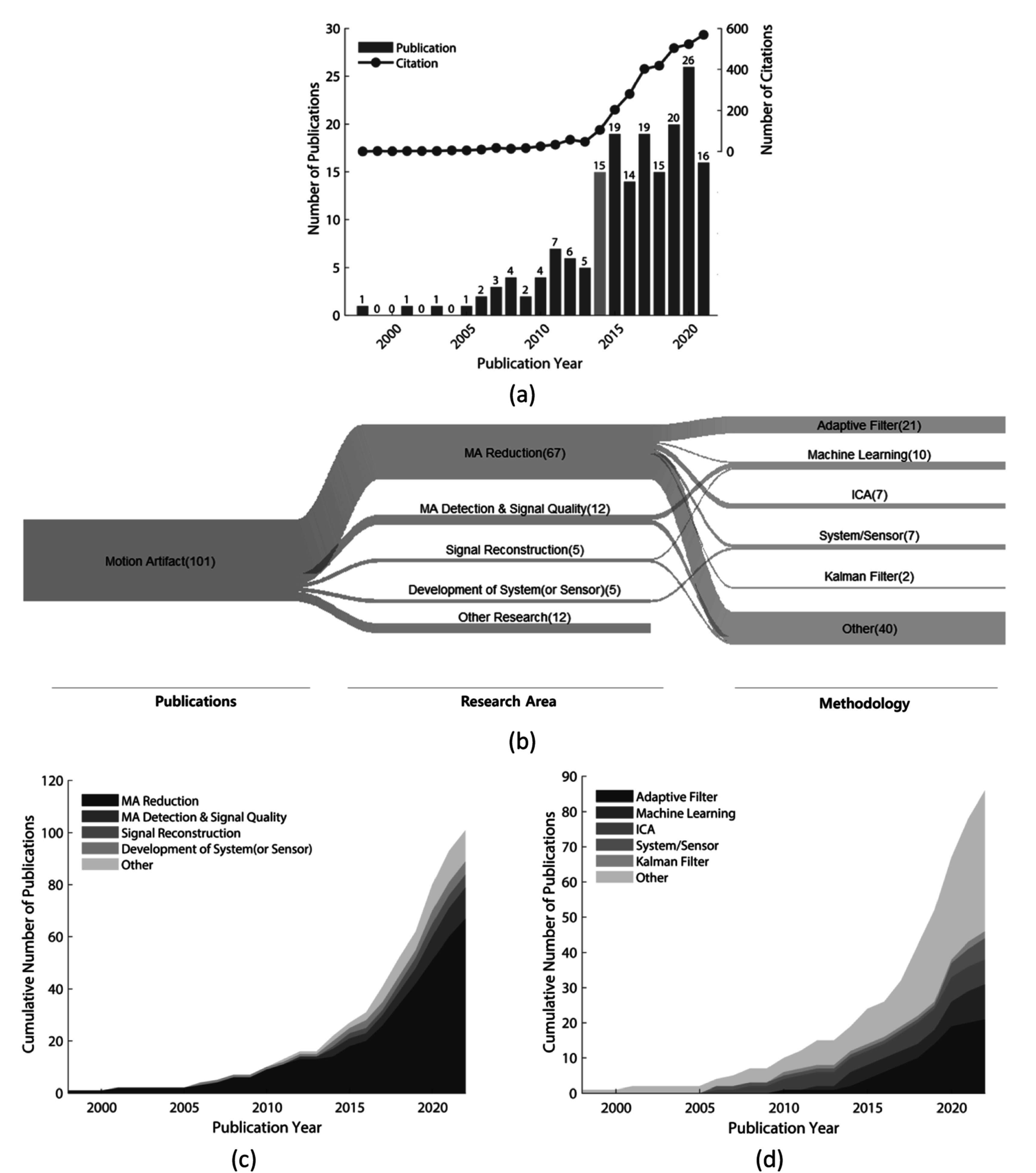
(a) Publication and citation trends of motion artifact related studies contained in the ‘Web of Science’ employing all field search terms, ‘photoplethysmography’ AND ‘motion artifact’, for the period 1998–2021. Citations are to source items indexed within the Web of Science. All article types have been included (accessed on 25/5/2022). The light blue bar indicates the moment of explosive growth. (b) Sankey plot for research area and number of publications of original articles for motion artifacts. (c) Histogram of cumulative publications in terms of research area, from 1998 to 2022. (d) Histogram of cumulative publications in terms of methodology, from 1998 to 2022. MA: motion artifact, ICA: independent component analysis.

More than 100 investigations related to MAs have been performed since 1998. Figure 14(a) presents the publication and citation trends of MA-related research, and it shows that the number of publications and citations on PPG MA has increased drastically since 2015. Figure 14(b) demonstrates that MA reduction and adaptive filtering have been the main research directions. Figures 14(c) and (d) present the cumulative number of publications, by year, according to the research field and methodology. They show that the number of research approaches that are not categorized is increasing. These approaches for handling MAs can be categorized as those improving the signal-to-noise ratio (SNR) and those evaluating the signal availability for the intended application. Some approaches aiming at signal enhancement improve the PPG quality in the signal-acquisition stage by redesigning the sensor and analog front-end. The signal-acquisition hardware is improved by modifying the structure of the sensor to ensure contact with skin even during movement (Lee *et al* 2022a). Other approaches aim to reduce the MA through traditional statistical or mathematical signal-processing approaches or by using auxiliary signals such as motion-reference signals or multiple PPG signals. Signal-processing methods to reduce MA are based on periodic moving average (Lee *et al* 2007), continuous wavelet transform (Zhang *et al* 2019), and/or Fourier decomposition (Pankaj *et al* 2022). A recent work presented the reconstruction of PPG signals with MA, using machine-learning-based approaches (Tarvirdizadeh *et al* 2020). Auxiliary signals measured from accelerometers (Arunkumar and Bhaskar 2020), gyroscopes (Lee *et al* 2018a), and piezoelectric sensors (Wang *et al* 2021) are also applied as motion-reference signals in adaptive filters for MA reduction. Moreover, MA-reduction techniques using multichannel signal processing based on multiple-wavelength light-sensing systems have also been proposed (Lee *et al* 2020).

To evaluate the signal availability, the signal quality is frequently assessed based on PPG features (Park *et al* 2022b). If the signal quality is not good, it is often impossible to extract the features; hence, evaluating the signal quality using the extracted feature may involve a methodological contradiction, i.e. a ‘fallacy of begging the question.’ Thus, recent research has applied machine learning directly to the original signal to evaluate the signal quality, achieving better performance than that of the existing feature-based studies, even without feature detection (Park *et al* 2022b). A problem associated with the current signal-quality assessment techniques is that the evaluation criteria for signal quality are subjective. Only limited research has been conducted on analyzing the PPG signal quality in relation to the pulse wave shape (Kyriacou and Allen 2021). PPG waveform morphologies without MA may also be different at different measurement sites on the body and for different subjects (Kyriacou and Allen 2021).

### Current and future challenges

**Figure 15.**
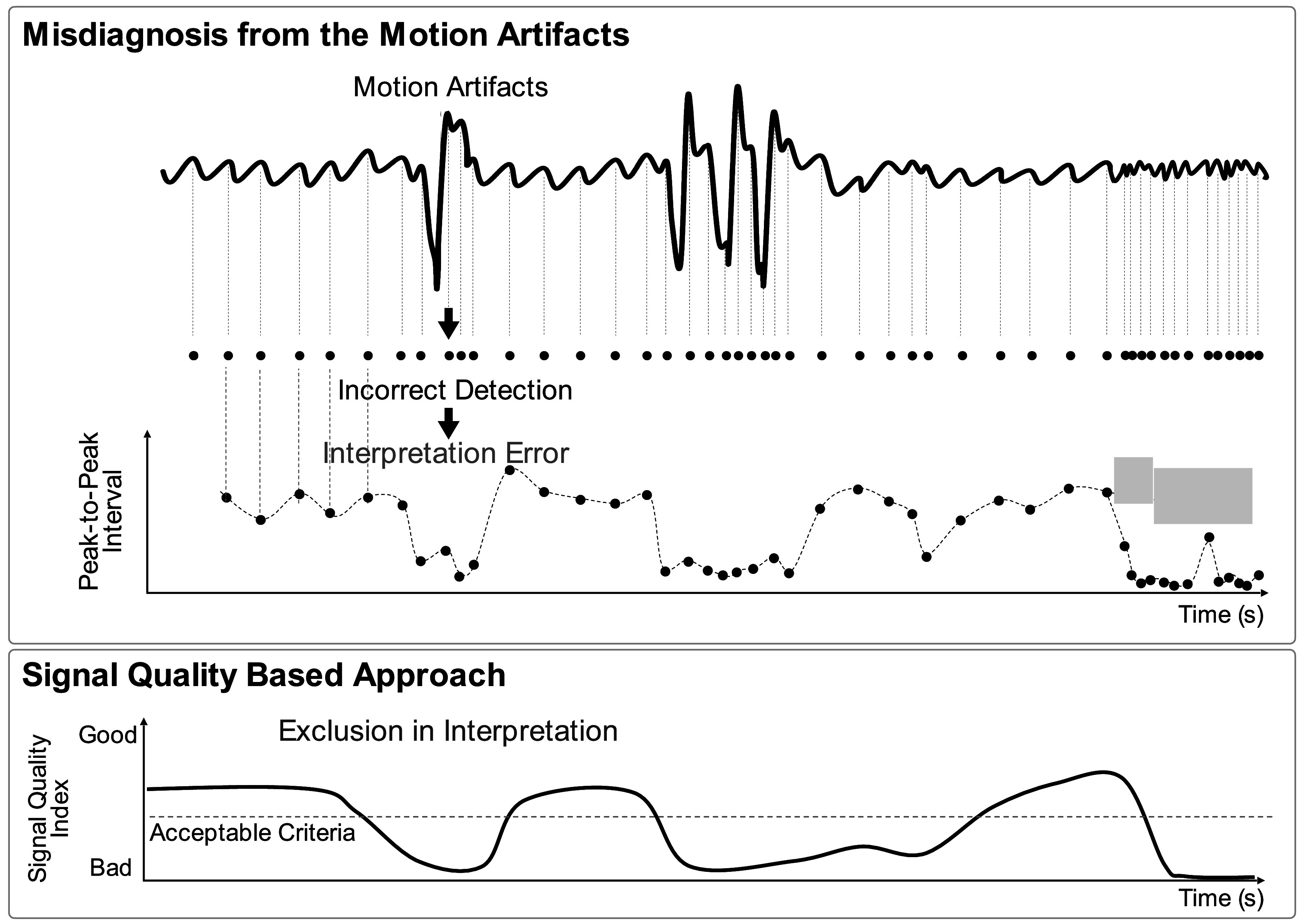
Example of interpretation error occurrence’s process using motion-artifact-induced feature detection error and interpretation error, and signal quality index based approach.

The major challenges of MA are related to the technical validation aspects. Traditional bio-signal processing research has focused on compressing noise and enhancing signal quality, i.e. improving SNR. However, when handling MA in wearable PPGs, a different approach is required because in wearable PPGs, important information can be frequently lost due to MA. In general cases, clean PPG can be extracted from noisy PPG through noise reduction if the noise has independent characteristics from the PPG. Unfortunately, in most cases of PPG MA, the frequency range of the noise overlaps the frequency of interest of PPG; thus, linear filtering is ineffective in removing artifacts (Jiang 2018). Moreover, if the level of noise is too high, it is difficult to extract the signal from the noise, and inaccurate information can lead to errors in result interpretation. Therefore, this needs to be focused on (figure 15). From this perspective, the recent PPG preprocessing research has paid attention to signal quality assessment. The goal of this assessment is to prevent errors in the interpretation of results caused by a ‘moderate level’ of noise removal and to provide only clear information. However, the challenge is that there is no classification for the standard of signal quality of PPG signals. For example, a high-quality signal might be required for analyzing pulse wave shape, whereas a lower quality signal can be acceptable for heart rate monitoring (Charlton *et al* 2022).

The second major challenge in handling PPG MA is validating the performance of MA reduction methods. Although many researchers have used treadmill for inducing motion artifacts, the magnitude of MA can vary depending on the motion characteristics, such as motion types, periodicity, or sensor location. Thus. only a limited evaluation can be performed. Particularly, since more errors occur in resistive training than in the treadmill (Zhang *et al* 2020), the diversity of dynamic noise induction protocols for method evaluation should also be considered.

### Advances in science and technology to meet challenges

In addition to the existing approach of removing the noise included in the signal, MA needs to be addressed in terms of determining whether a signal is available or not. Technically, in the future, MA reduction may be possible using an integrated method based on signal and noise separation, SNR improvement, and signal restoration or generation. The transition of this technical perspective suggests that MA can be addressed by expanding from the conventional signal processing to data processing based on classification and regression. The combined technology of big data and deep learning, which can analyze the quality of a signal without an additional feature extraction process and restore the signal through regeneration, is highly likely to be employed as an effective alternative to handling the MA of wearable PPGs. However, it is also necessary that there exists an agreement between researchers on a common evaluation protocol and approach for the objective performance validation of MA-reduction approaches. Recently, the International Electrotechnical Commission (IEC) TC 124 developed a test protocol and evaluation standard (1-346,353-402, (IEC63203)) for assessing the accuracy of PPG-based heart rate monitoring. The test evaluation protocol of the standard to be established is expected to be employed in the research on PPG MA, in the future. Furthermore, as wearable PPG is being emphasized more in daily living, it is necessary to fully consider the commitment associated with the data-availability evaluation method based on real-world data in terms of data availability.

### Concluding remarks

Despite the efforts to remove MAs from PPG signals, there has been no clear solution yet. The efforts are being expanded to include an approach involving bypassing methods focusing on avoiding after defining rather than eliminating. These trends indirectly suggest that it is difficult to eliminate MA at the current level of technology. Traditionally, MA has been recognized as a noise to be removed, but it can be viewed as evidence that the user is active in wearable PPG for daily use. This means that when dealing with MA in wearable PPG, it will be effective to look at it from the point of view of improving the signal availability, and beyond the conventional noise removal that aims to simply increase SNR. An effective response to MA of wearable PPG may begin with such a shift in perspective.

## Acknowledgments

This research was supported by a grant of the Korea Health Technology R&D Project through the Korea Health Industry Development Institute (KHIDI), funded by the Ministry of Health & Welfare, Republic of Korea (grant number: HR20C0026, HI22C1668).

## Conflict of interests

The authors declare there’s no conflict of interests. The views and opinions expressed are those of the authors and do not reflect those of the Ministry of Food and Drug Safety the in Republic of Korea.

## APPLICATIONS

## 11. Consumer applications

### Ilkka korhonen

Faculty of Medicine and Life Sciences, Tampere University

### Status

Although photoplethysmography (PPG) has been widely used in hospital settings since the 1980s, consumer applications of PPG were only introduced around 2010. The first consumer applications were armbands which used mainly infrared light for PPG to measure heart rate (HR). These devices were very prone to motion artefacts and did not gain significant market penetration. The first optical wrist-worn HR monitors with reasonable accuracy were released in 2013 (Parak and Korhonen 2014). The breakthroughs enabling acceptable accuracy were the use of green light for PPG and advancement in motion cancellation algorithms. PPG based on green light was found to be significantly less prone to motion artefacts than IR (Maeda *et al* 2011). Since then, optical HR monitoring has become a standard way of monitoring HR in consumer applications ranging from exercise HR monitoring to 24/7 tracking of stress, physical activity, and sleep. Today, most smart watches are equipped with an optical HR sensor, and PPG is also used in other form factors such as ear-pods, armbands, and rings, which are available for consumer use. Optical HR monitoring has revolutionized consumer HR monitoring and its market and made HR monitoring an everyday option for hundreds of millions of consumers.

The accuracy of optical HR monitoring based on PPG has been significantly improved since 2013. Motion cancellation based on filtering and machine learning algorithms has been significantly enhanced together with advancements in optomechanical design and electronics performance, and numerous studies show that especially during rhythmic activities the best optical HR monitors reach accuracy comparable to ECG-based methods in HR monitoring (Zhang *et al* 2020). When monitoring beat-to-beat intervals during rest, accuracies around 6 ms mean absolute error (MAE) have been reported (Parak *et al* 2015) while during motion and arrhythmias, significantly higher errors and lower coverage (i.e. amount of time when estimation is available) are common. Large differences however exist between different brands and devices, highlighting a need for critical evaluation when interpreting results, and objective scientific validation of commercial devices prior to their wide use, such as for research purposes. In addition to HR and heart rate variability (HRV) monitoring, metrics based on advanced analytics of these parameters (such as stress, energy expenditure, maximal oxygen consumption, etc), and also other PPG-based consumer applications have been introduced recently. For instance, several smart watches and rings offer estimates of blood oxygen saturation (SpO_2_) for consumer use, e.g. for monitoring training in and adjustment to high altitudes. Some devices also offer blood pressure (BP) monitoring based on pulse wave analysis of the PPG signal directly for consumers for informative monitoring.

### Current and future challenges

Despite the progress during last decade, optical 24/7 HR monitoring in consumer devices during daily life activities has been and still is challenging (Lemay *et al* 2014). The PPG signal is prone to optical noise, such as due to rapid changes in ambient light levels), and also motion artefacts which modify the sensor contact pressure, moving the sensor against the skin, or causing the tissue itself to move during motion either due to gravity or muscle, tendon or ligament motion within the tissue close to the sensor. As a result, the signal-to-noise ratio of the PPG may be very low, and often the heart activity related component in the PPG is completely lost due to artefacts. Inter-individual factors such as skin tone and anatomical differences cause variations in performance, while intra-individual factors such as blood perfusion changes, e.g. due to external temperature changes, challenge the monitoring accuracy. Good performance requires excellent mechanical design of the device (e.g. strap and housing) and proper wearing such as strap tightness optimization and correct sensor location. While these factors are important for any PPG application, they are especially critical for long-term consumer applications where consumers’ adherence to wearing instructions is usually low, and usage comfort dominates the design over accuracy concerns. Hence, the true accuracy of consumer applications of PPG-based HR, SpO_2_ and BP monitoring is likely very compromised compared to what is seen in controlled evaluation studies.

Despite these challenges in data reliability, the wide availability and use of PPG-based consumer devices has generated datasets which have been beyond reach earlier. These data, accumulating mostly for companies selling these devices, are valuable assets in studying longitudinal behavioural health patterns in large numbers of consumers, up to millions (Perez *et al* 2019, Natarajan *et al* 2020, Radin *et al* 2020, Ong *et al* 2021). One of the future opportunities and challenges is to better understand the reliability of such data, and to discriminate when the reported values may be trusted and when not.

### Advances in science and technology to meet challenges

Currently, researchers and wearable tech companies are focusing on pushing the boundaries of sensor wearability, comfort, battery lifetime, and also accuracy and coverage even further. Advances in miniaturization of the electronics and reduction in their power consumption not only help to improve wearability but to reduce the sensor weight and therefore motion artefacts; an extreme of this development are tattoo-types of sensors (Laurila *et al* 2022). Also, form factors other than the wrist-watch are entering wide consumer use. Today, hearables have passed wrist-worn devices as the most common consumer wearable, and integration of PPG sensors into them is becoming popular. The ear is an excellent location for the PPG sensor due to the reduction in motion artefacts as compared to the wrist, and potentially better PPG signal-to-noise ratio due to better perfusion in the ear area in most conditions. However, mechanical design to meet variations in consumer anatomy comfortably and economically continues to be a challenge. Smart rings provide a very high signal quality especially during rest thanks to strong blood perfusion in fingers, and finger anatomy also enables transmissive PPG instead of reflective, which in particular improves SpO_2_ accuracy. Novel ring sensors have been miniaturized successfully, allowing excellent wearability and ease-of-use, and it may be expected that ring sensors will be increasingly popular among consumers in the coming years. Integration into other form factors, such as smart glasses, will also be a future trend—it is safe to assume that PPG sensors will be added to any wearable device which is in contact with the skin to monitor user’s health, wellbeing, and activity. As a result, availability of the wearable wellness data based on PPG will become even wider and cover still longer periods of time and usage situations. This will offer unforeseen opportunities for both epidemiological research and design of data-driven health interventions and services.

Another key area for advancing consumer applications of PPG is related to advancements in the algorithms. While wearable sensor platforms become ever more capable in terms of memory and processing power, more advanced algorithms to process PPG data in real-time in embedded sensors are made possible. Today, most of the algorithms to process PPG to cancel motion artefacts and detect HR are based on relatively classic signal processing algorithms such as adaptive filtering, spectral analysis and knowledge-based decision trees, which are adapted to personal history little or not at all. Research today focuses on learning algorithms which adapt and learn from the personal history, and apply machine learning techniques for improved detection of HR, SpO_2_, or BP e.g El-Hajj and Kyriacou (2020). Furthermore, multichannel monitoring of PPG has great potential to improve signal-to-noise ratio and coverage of the PPG, and to provide new information not only for HR and SpO_2_ monitoring but also for applications such as BP monitoring (Liu *et al* 2016a).

### Concluding remarks

Consumer applications of PPG have been focused on monitoring of HR, HRV and parameters derived from them, such as physical activity, sleep, and stress. Advances in technology are now introducing new parameters such as SpO_2_ and BP for consumer applications and devices available over the counter. The main differentiators between consumer and medical wearables are consumer-favourable design, wearability, availability and cost. In addition, consumer wearables are often integrated with other, non-health related features such as smart watch features, which make consumer adherence to and coverage of data from these applications in long term monitoring superior as compared to their medical device counterparts. The accuracy of consumer applications and medical devices is also expected to narrow. The wide adoption of PPG-based devices by consumers offers increasing opportunities for real-life data collection to support research on health, health outcomes and the impact of interventions. However, objective scientific research is needed to provide an evidence base for their use in research, such as evaluation of the accuracy of consumer devices.

## 12. Detecting cardiac arrhythmias

### Antti Vehkaoja^1,2^, Harri Saarinen^3^ and Jussi Hernesniemi^1,3^

^1^Finnish Cardiovascular Research Center Tampere, Faculty of Medicine and Health Technology, Tampere University

^2^PulseOn Ltd

^3^Tampere Heart Hospital, Wellbeing Services County of Pirkanmaa

### Status

Detecting cardiac arrhythmias, especially atrial fibrillation (AF), with wearable photoplethysmography devices has been a popular research topic for almost a decade. AF is linked with an over five-fold increase in the risk of stroke and there are also many other serious health conditions that are thought to be preceded by an increase in asymptomatic arrhythmic activity of the heart. These potentially preventable conditions include cognitive impairment by microemboli due to asymptomatic atrial fibrillation, sudden arrhythmic cardiac death and worsening of heart failure leading to preventable hospitalizations. Especially in the early stage, AF usually appears intermittently and is therefore detected effectively only by wearable devices enabling long-term monitoring. Today, several commercial smart watches or fitness trackers from manufacturers such as Withings, Apple, and Fitbit feature AF detection as a part of their photoplethysmographic (PPG) heart rate sensors. Currently, there are not many devices that are intended exclusively for clinical use. One such solution is the PulseOn Arrhythmia Monitor System launched in early 2022. Several others are however expected to appear soon.

According to the guidelines of the European Society of Cardiology (ESC), the diagnosis of AF requires rhythm documentation with an electrocardiogram (ECG) tracing showing AF. For AF, a single-lead ECG of at least 30 seconds recorded with any device and any type of electrodes is sufficient (Hindricks *et al* 2021). To support diagnostics, many wearable PPG devices that feature detection of irregular pulse now also have the ability to record an ECG. There are also wearables and handheld devices that only facilitate intermittent ECG measurement, but a major benefit of combining PPG-based AF detection and notification together with ECG is to be able to detect also asymptomatic episodes and episodes occurring during night. Further, European Heart Rhythm Association’s Practical Guide (Svennberg *et al* 2022) proposes that PPG data without ECG can be used for heart rate and rhythm monitoring after AF diagnosis. Consensus statements provided in the Practical Guide also state that: ‘PPG-based or ECG-based devices are preferred to pulse palpation for AF screening’, ‘In systematic screening for AF, PPG-based or ECG-based devices can be used’, and ‘If PPG screening is indicative of AF, an ECG-based method should be used to confirm the diagnosis of AF’. Although these consensus statements are not yet recognized in the guidelines of the ESC or the American Heart Association, they have a chance to be included in those as the evidence on the practical benefits of PPG-based screening builds up. From the economical point of view, population-based screening for AF using wearable devices has also been found feasible in a simulation study by Chen *et al* (2022) for individuals of ≥65 years of age and having a CHA_2_DS_2_-VASC score warranting anticoagulation medication. From the methods evaluated in the study, wrist-worn wearable PPG followed by conditional wrist-worn wearable ECG and confirmatory patch monitor was found the most cost-effective strategy.

Current guidelines for the pharmacological treatment of AF are from the era when there was no feasible means of studying e.g. the effect of AF burden on the risk of stroke. PPG-based methods can provide the means for doing this and thus helping in optimizing the guidelines. It must however be emphasized that an AF diagnosis often leads to life-long oral anticoagulant medication, which in turn predisposes the patient to the increased risk of haemorrhage. The clinician therefore has to be certain of the type of arrhythmia when making the diagnosis.

**Figure 16.**
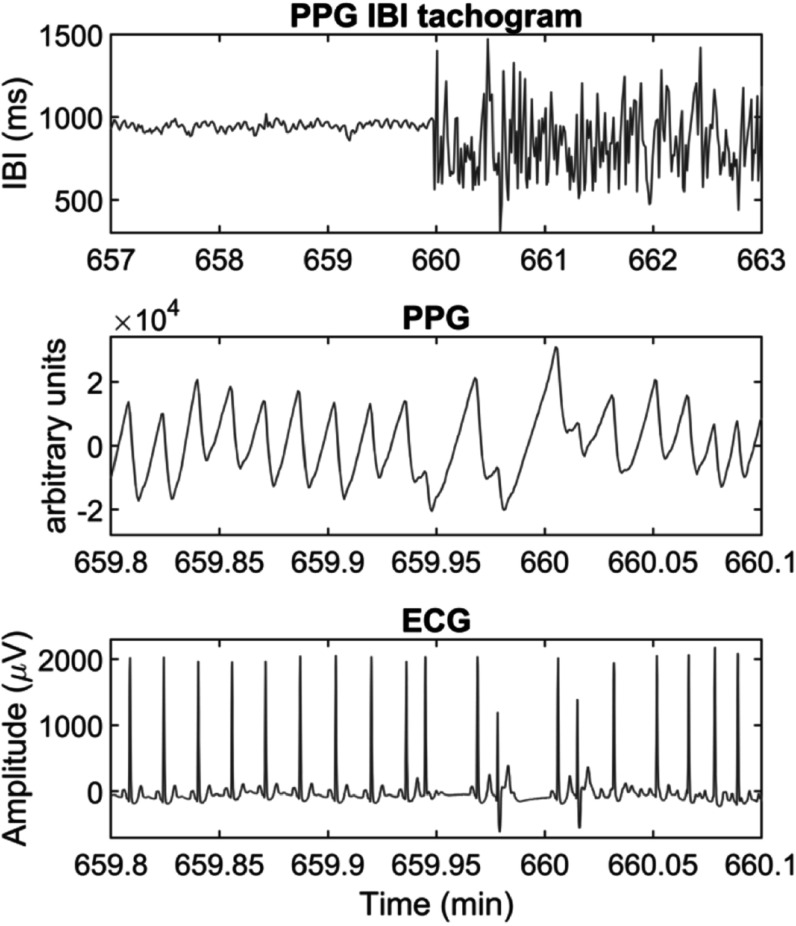
Example of the onset of atrial fibrillation demonstrating the variation in the PPG pulse wave amplitudes due to inefficient filling of the ventricles. Single ectopic beats produce similar variations in the PPG signal waveform.

Most algorithmic approaches for detecting cardiac arrhythmias from PPG records rely on interpretation of heartbeat interval data. In addition, some studies e.g. by Eerikäinen *et al* (2019) have combined heartbeat interval information with features extracted from the PPG signal waveform. These approaches are illustrated in figure 16. The focus has been mainly in the detection of AF, which is the most prevalent sustained arrhythmia. The data collection for algorithm development and performance assessment has often been done in the inpatient setting but the most representative results from the practical application point of view are obtained in free-living conditions. Review articles by Eerikäinen *et al* (2020), Lopez Perales *et al* (2021) and Pereira *et al* (2020) list a number of articles on PPG-based AF detection. Both sensitivity and specificity vary usually between 95% and 99%, medians being 96.2% and 97.7% (Eerikäinen *et al* 2020) but direct comparison of the results should be done with caution because the exact results are affected greatly by the patient sample, e.g. the amount of other arrhythmias present in the data and recording conditions as well as data analysis strategy, e.g. length of the analysed data segments and requirement for minimum AF episode duration. We recently obtained 95.6% sensitivity and 99.2% specificity for AF detection in outpatient setting for 5 min data segments. In outpatient setting usually roughly 50% of the data segments need to be discarded due to excessive interference in the PPG signal.

Preliminary research data also exists on the detection of other arrhythmias such as atrial flutter (AFL), quantification of the number of ectopic beats (Nazarian *et al* 2021) as well as estimating the burden of AF (Zhu *et al* 2021), but these features are not widely in use in certified medical devices. Detection of the aforementioned arrhythmias would be beneficial in clinical work, which makes PPG-based arrhythmia detection still a relevant topic of research.

### Current and future challenges

In PPG-based arrhythmia detection, the challenges regarding the quality and reliability of the data are mostly the same as in many other PPG applications. These include high sensitivity to movement (i.e. physical activity), the effect of skin colour and the effect of low superficial blood perfusion. In the case of arrhythmia detection, there is however one additional specific feature in the data affecting the accuracy. Due to the imperfect filling of the ventricles, the strength of the pulse waves varies during arrhythmia, which makes it often more difficult to accurately detect individual heartbeats. This is demonstrated for example in article by Harju *et al* as well as recently by Charlton *et al* (Harju *et al* 2018, Charlton *et al* 2022), which both compared the heartbeat interval estimation accuracy during sinus rhythm and atrial fibrillation. The effect is also illustrated in figure 23. In addition, because heartbeat intervals are highly irregular during AF, the use of frequency domain analysis methods, often used for the estimation of average heart rate, are not effective.

As said, so far PPG-based arrhythmia detection research has mainly focused on AF. The challenge with AFL, which is another relatively common arrhythmia, is that it is often manifested as a very stable rhythm caused by a supraventricular re-entry activation combined with every second, third or fourth flutter activation triggering a ventricular contraction. Due to the resulting stable rhythm, methods based on the detection of increased heartbeat interval variability are not effective. The diminished variation, which is clearly seen in ECG-based Poincaré (or Lorenz) plot might be used for detecting AFL but the inevitable uncertainty of PPG-based heartbeat interval estimation caused by variance in pulse arrival time and PPG waveform disturbances induce a challenge for reliability. Features extracted from the pulse waveforms could provide additional information for identifying AFL. Pulse signal waveforms vary between individuals, but data recorded during sinus rhythm could be used to provide individual baseline information on the waveform.

Reliable detection of single ectopic beats has been a challenge with PPG-based arrhythmia algorithms. As such, ectopic beats can be detected from patterns in heartbeat interval tachogram (Haddad *et al* 2019) but the actual problem has been to be sure whether the pattern is a result of a true ectopic heartbeat (i.e. ventricular or supraventricular extra systole—VES or SVES, also called as PAC/PVC for premature atrial and ventricular contraction, respectively), or an error in the beat detection due to simultaneous movement or other artifact. Distinguishing VESs and SVESs from each other would be important as large number of VES can be a sign of severe heart diseases. Monitoring the effect of catheter ablation performed for treating excessive ventricular extra systoles would be an additional use case. VES often results in more attenuated PPG pulse waves compared with SVES, or a complete lack of the pulse wave. Solosenko *et al* have proposed a method for the detection of VES Sološenko *et al* (2015). However, their method was validated with transmittance mode PPG in which the signal is usually stronger than in reflective mode PPG commonly used in wearable devices. A recent doctoral dissertation done in the same research group proposed and evaluated methods for detecting life-threatening extreme bradycardia and ventricular tachycardia episodes as well as and assessing the burden of ectopic beats using wearable PPG device Paliakaitė (2023). They also published a PPG simulator tool for assessing detection algorithms. Yet another challenge in PPG-based arrhythmia detection is that due to its high sensitivity to movements, reliable beat-to-beat heartbeat interval detection cannot be performed during high-intensity activities. Therefore, short arrhythmia episodes may be left unnoticed especially if occurring during exercise or other activities.

### Advances in science and technology to meet challenges

The fundamental challenge of PPG measurement, sensitivity to movement artefacts, is very difficult to overcome completely but several technological advances can help to mitigate its effect and enable more versatile applications. The PPG signal quality can currently be relatively well optimized by actively controlling the essential parameters of the PPG measurement: LED driving current and the gain of the measurement amplifier as well as compensating the DC offset to maximize the PPG signal amplitude. Multi-wavelength PPG measurement provides an opportunity to further improve the performance of PPG measurement. As reflection of different wavelengths is mainly received from different depths in the tissue, combining their information enables both compensation against movement artefacts up to some extent as well as combatting against the challenges brought by variations in skin pigmentation and blood perfusion. The use of multi-wavelength PPG technology could also enable algorithms for differentiating true heart-originated ectopic beats. In addition to section 4, which is dedicated to wearable multi-wavelength PPG, Ray *et al* has recently gathered a comprehensive review on research around the topic (Ray *et al* 2023).

Because periods of low levels of movement may be short, it will be important in the future to develop arrhythmia detection algorithms that are able to make reliable rhythm assessment from short segments of good quality PPG data. In addition, because there may be short, few second periods in the PPG which have been corrupted by movement artefacts, the algorithms should be able to recover from artefacts quickly and allow a certain amount of artefact whilst still being able to make a rhythm assessment.

**Figure 17.**
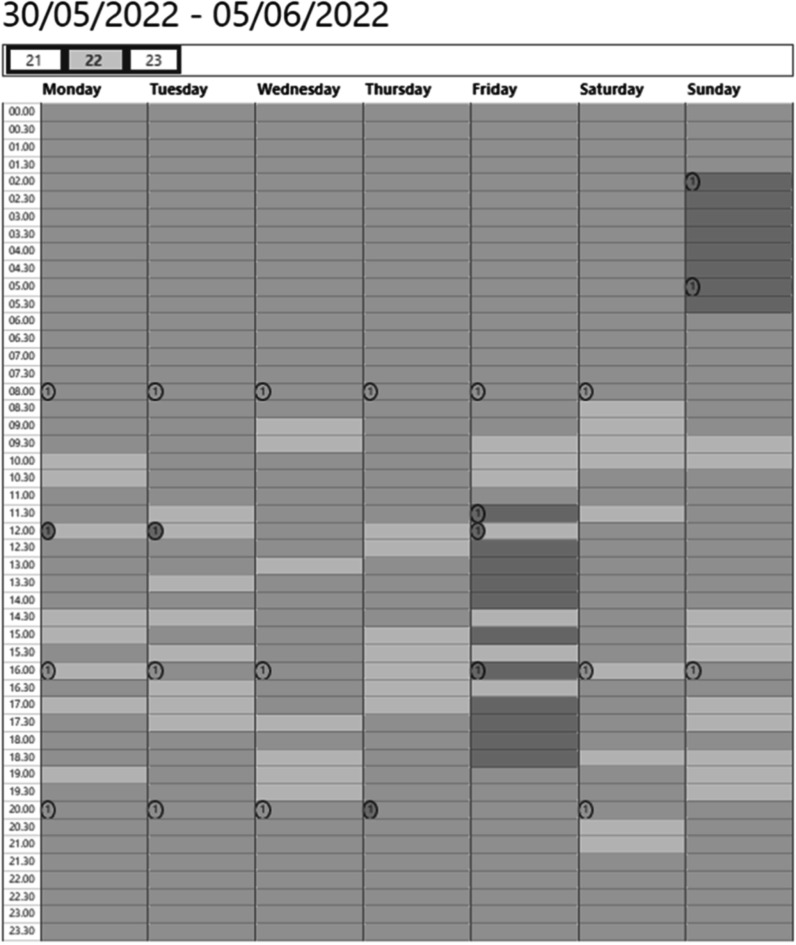
An illustrative example of how the occurrence and duration of AF episodes estimated with PPG data can be visualized to clinicians. Subsequential AF burden can be estimated from the data. Blue sections: regular rhythm, red sections: irregular rhythm, grey sections: undetermined rhythm due to inadequate amount of good quality PPG data. Circles represent ECG measurements with results of automatic analysis (also arrhythmias other than AF). Image modified from the week view of the PulseOn Arrhythmia Monitor System.

Although current care guidelines by ESC or American Heart Association do not consider AF burden in the indication for permanent anticoagulation, the evidence is building that AF burden is independently associated with the risk of thromboembolism and ischemic stroke (Healey *et al* 2012, Go *et al* 2018). It is still unclear what duration of AF episode warrants anticoagulation to protect from thromboembolic events (Svendsen *et al* 2021). Reliable estimation of the AF burden based on continuous PPG data could thus become a useful tool in clinical decision-making in the future. Figure 17 shows an illustrative example of PPG based visualization of AF episodes.

In the future, the use of pulse waveform information together with heartbeat interval data could enable ectopic beat detection with improved confidence and for example the detection of supraventricular tachycardia episodes that are known to be associated with an increased risk of developing AF or bradycardias and cardiac pauses that may lead to syncope and sudden cardiac death. Long-term statistical analysis of the aforementioned information combined with behavioural data could thus further be used to assess individual’s risk for developing pathological arrhythmias.

### Concluding remarks

PPG-based arrhythmia detection has been an extensive research interest for several years, already producing several commercial solutions for the detection of AF episodes. However, the full potential of PPG for detecting cardiac arrhythmia is yet to be exploited. Future research should focus on algorithms for reliable detection of other arrhythmias besides AF, such as AFL and the number of ectopic beats, as well as the estimation of AF burden, which is likely to become more important in clinical decision making in the future. In the development of new solutions, it is important to verify the performance with varying populations and conditions. The unobtrusive PPG-based technology has potential to enable convenient, reliable, and cost-effective solutions for large-scale screening and assist in the diagnosis and monitoring of AF and other arrhythmias as well as potentially in the evaluation of the risk of sudden cardiac death.

## Acknowledgments

The authors would like to acknowledge Tuomas Halkola from PulseOn company for assisting in preparing figure 17.

## 13. Sleep assessment from the PPG

### Gari D Clifford^1,2^

^1^Emory University

^2^Georgia Institute of Technology

### Status

**Figure 18.**
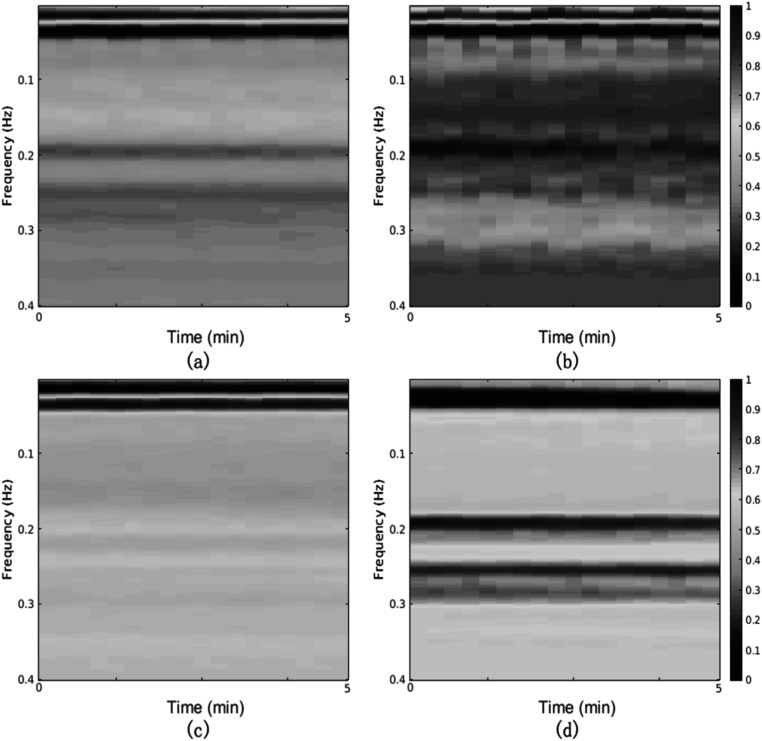
Example of average respiration-heart rate cross spectrum in the time-frequency domain during different sleep states: (a) wake; (b) REM sleep; (c) NREM light sleep; (d) NREM deep sleep. Hotter colors indicate higher cross spectral coherence (inherently normalized between 0 and 1). Adapted from Li *et al* (2018).

While published research in the use of cardiovascular signals for assessing sleep stretches back to the 1980s (Moody *et al* 1986, Thomas *et al* 2005), over the last decade there has been an explosion of consumer-level wearables containing photoplethysmographic (PPG) sensors which has been associated with a commensurate increase in publications on using the PPG for assessing the depth of sleep and sleep ‘architecture’ (Dehkordi *et al* 2014). Most approaches have focused on heart rate and respiratory variability-related features (Beattie *et al* 2017, Fonseca *et al* 2017), or more rarely, measures of cardiorespiratory coupling, such as in figure 18 (Li *et al* 2021). Therefore, these approaches are highly similar to the electrocardiographic-based forerunners (Li *et al* 2018).

Recent efforts to apply deep learning to PPG have ranged from learning from beat-to-beat intervals (Fonseca *et al* 2017) to raw data (Korkalainen *et al* 2020) to physiology-driven features such as time-frequency cardiorespiratory coupling (Li *et al* 2021). The metrics reported in the literature vary, but commonly include Cohen’s Kappa (*k*) and Accuracy (Acc). Some of the more transparent papers have also identified errors in derived metrics such as total sleep time, REM efficiency and sleep latency, to provide a deeper insight into how any performance translates into downstream diagnosis. Of course, all these metrics are a function of the distribution of sleep classes within the testing and training databases, and they are rarely reported. Moreover, there are no articles at this time which have evaluated how the quoted performance maps into a diagnostic or screening performance. The published results have varied widely, with the state-of-the-art being reported to be *k* ≅ 0.7, which is close to inter-human expert concordance level. However, without public code and data, it is impossible to verify these surprising claims, and may reflect imbalances in sleep state prevalence in the data (see challenge three in the next section). It is therefore recommended that in future studies, at least one pathological class is included in the population and any sleep staging algorithm is also assessed in terms of a diagnostic outcome on the population being studied (e.g. for identifying insomniacs from normal). While this doesn’t fulfil the entire diagnostic criteria, it does provide an insight into how the errors in a given sleep staging algorithm affect a down-stream diagnosis.

### Current and future challenges

There are multiple challenges that confront efficient and effective sleep monitoring from the PPG. First, to generate a high-quality signal for the PPG requires good contact with the skin. Even when wearing on the wrist (which is not the optimal place for transducing blood movement), the sensor can create localized heating, sweating, and cause discomfort. This leads users to wear the device loosely. This can be partially solved by using adhesive materials (see section 2), but such modalities are for short-term monitoring only (hours to days) because of the skin irritation that results. The quality of a PPG signal is also a function of where on the body a signal is captured, and of skin pigmentation, which can lead to significant biases and even structural discrimination through dismissed symptoms, under-diagnosis and under-treatment (Feiner *et al* 2007). In addition, the quality is also a function of wavelength, yet little work has been performed on the effect of choice of wavelength (or multispectral wavelengths) on sleep staging from the PPG. Of course, signal quality at night is often better than during the day, because of lack of movement. However, since low quality is associated with sleep disruption, it can be a confounder for the end goal of diagnosis from sleep stages. Therefore, it is important to account for this bias when classifying the sleep stages. One approach to dealing with the above issues is to use off-body video-based PPG approaches. However, these lead to other issues such as the need to have uncovered skin in view, accurately identifying the region of interest, movement tracking issues, video resolution and compression issues, and concerns over privacy. In particular, the fact that most people rotate their face and body in bed means that any system that wanted to use off-body PPG (rather than movement) to stage sleep would need multiple cameras, and the ability to see though hair, bed linen, and any other visual obstacles. Considering these issues, it seems much more practical to use radar or seismocardographic measures of physiology and movement for off-body sleep staging. Moreover, the enormous variation in camera hardware, pre-processing and lighting conditions make any comparisons between studies very difficult.

Second, many approaches in the literature do not attempt to differentiate artifact from a real signal, and so can confuse activity with a real signal. Without the use of related signals, such as accelerometer data to identify movement artifact, limb movement, and other useful metrics not readily derived from the PPG, it is more likely that a system will learn artifact biases in the training and test data, and reduce generalizability (Li and Clifford 2012). Moreover, the only clinically accepted indicator of sleep stages is the electroencephalogram (and related signals such as the electrooculogram), since sleep stages are believed to be a brain state, not a physiological cardiovascular state, and therefore cannot be definitely identified by a cardiovascular signal alone, particularly in pathological patients. A key example would be with an insomniac who is likely to be lying still and may exhibit sleep-like physiology because of the phase of their circadian rhythm.

Third, a key problem, inherent in all sleep staging (and most, if not all medical fields that rely on human labels) is the high inter-rater disagreement levels. For sleep staging, values of *k* tend to be in the range of 0.6–0.7 on average, but can vary highly by sleep stage with *k* = 0.70, 0.24, 0.57, 0.57 and 0.69 for the Wake, N1, N2, N3 and REM stages, respectively (Lee *et al* 2022b). None of the literature on the topic of wearable sleep staging (particularly PPG) has directly addressed this issue. Moreover, there is an inherent problem in many approaches to PPG-based or physiology-based sleep staging, in that 30 s (the standard sleep staged epoch length) of physiological data may not be long enough to assess a sleep stage, even when using raw (30 Hz+) data, because physiology changes on a timescale well beyond 30 s.

A fourth problem, rarely addressed, is the effect of abnormal physiology on sleep. A simple example is the effect of cardiovascular rhythm and abnormal beat types on physiology-based sleep staging. Arrhythmias, or even lack of variability or respiratory sinus arrhythmia (as is seen in the elderly or those with neuropathy, for example), are likely to confound any approach based on such variability changes within sleep states. Since sleep is being used as an intermediate metric to diagnose a medical problem, having a medical problem that affects the physiology of sleep and cardiovascular rhythm/variability creates a serious confounder. Either an algorithm must be trained on all possible pathologies, or pathology-specific algorithms must be used, which are selected through a separate screening process. In the latter case, the algorithm can be used for tracking changes over time in the appropriate population, but cannot be used as a screening or diagnostic agent. This appears to be a promising future direction, which would include a screening interview, followed by a baseline setting simultaneous EEG and PPG recording, then a personalized PPG-based sleep algorithm that could be developed through transfer learning, for example.

Fifth, there is little coherence in the literature on the metrics and databases used or the rationale (and description of) how different patients within databases were excluded or distributed during training, particularly with respect to demographics and health conditions (e.g. see table 2.) Finally, there is the issue of bias and interpretability. Although explicit biases (such as response to skin pigmentation) are clearly addressable, contemporary end-to-end machine learning (which dominates current research by volume, but not necessarily quality) introduces the tricky issue of false discovery—i.e. the possibility of learning features associated with the target that are not associated with sleep stages beyond the limited cohort chosen for training and testing. Explicit features that have been chosen range from pulse timing variability (Radha *et al* 2021) to morphological changes (Korkalainen *et al* 2020), to the interactions between morphological and timing (Li *et al* 2021). While these may seem less prone to false discovery, variability, morphology and the strength of coupling is known to change with age and medical condition, and therefore may introduce biases. However, at least these biases are known and are therefore more easily accounted for.

**Table 2.** Overview of PPG-based classification of sleep stages. OSA: obstructive sleep apnea, SHHS: sleep heart health study, ETSF: emory twin study follow-up dataset, PTSD: post-traumatic stress disorder, SOMNIA: sleep and obstructive sleep apnea measuring with non-invasive applications, N2N: night to night dataset, HHS: heart health study dataset, MESA: multi-ethnic study of atherosclerosis, CFS: cleveland family study. *Private indicates data is not available in the public domain*.

Work	Inputs used	Database name	Database demographics	No. stages	Acc (%)	*k*
Korkalainen *et al* (2020)	raw PPG	Private	894 suspected OSA patients	3	80.1	0.65
				4	68.5	0.54
				5	64.1	0.51
Li *et al* (2021)	HRV metrics from PPG + activity features from accelerometer	SHHS visit1 ETSF	5793 subjects for pre-training (SHHS), 105 subjects (ETSF, PTSD twin study)	2	81.5	0.58
				3	77.1	0.50
				4	68.6	0.44
Radha *et al* (Radha 2021)	HRV metrics from PPG	Siesta Eindhoven	292 subjects (195 healthy + 97 sleep disorder patients), or 584 recordings (Siesta) 60 subjects (healthy), or 101 recordings (Eindhoven)	4	76.36	0.65
Wulterkens *et al* (2021)	HRV metrics from PPG + body movement features from accelerometer	SOMNIA + N2N + HHS	422 sleep disordered patients (from SOMNIA) + 121 healthy (from N2N + HHS) for training, 292 patients (from SOMNIA) for validation	4	76.4%	0.62
Huttunen *et al* (2021)	Raw PPG	Private	2149 suspected OSA patients for pre-training, 877 suspected OSA recordings	3	83.3	0.72
				4	74.1	0.64
				5	68.7	0.60
Kotzen *et al* (2022)	HRV metrics from PPG and raw PPG	SHHS Visit 1 MESA CFS Visit-5 v1	5758 subjects for pre-training (SHHS), 2054 patients (MESA) + 320 patients (CFS)	4	84	0.75

### Advances in science and technology to meet challenges

To address these challenges, it is clear that far more research is required, particularly in multispectral PPG, and its performance should be assessed based on skin pigmentation, skin age, hair cover, and body location. In addition, the inclusion of multiple sensors (from on-body accelerometry, to off-body full-body movement such as radar-based monitoring) and the use of multimodal machine learning may provide an optimal method for combining these data to classify sleep stages. Moreover, there is a need to integrate downstream metrics (such as diagnoses) into the optimization process (e.g. see Cakmak *et al* 2021). To improve the issue of inter-rater variability, studies need to focus on using at least three independent experts, and develop machine learning approaches that factor in the uncertainties from disagreements, identifying transition points in 30 s epochs to address the problem of within-epoch class confusion. To address the issue of arrhythmias, a multiclass optimization approach (sleep, artifact, rhythm) could be applied. Addressing the issue of scientific repeatability is common to all studies and is generally addressed by well documented open-source code and open access data. Finally, to address the bias, and patient diagnoses issues, a transfer learning approach can help, providing boosting on a relatively small sub-population (Li *et al* 2021).

### Concluding remarks

In summary, there is significant potential in the use of PPG (and related sensor streams) for sleep staging, but significant barriers remain. The current push to keep raw data inaccessible in commercial devices continues to hold the field back and is likely to exacerbate existing biases. There is a clear need for standardized databases, open access code, open-source software, and reference hardware to help accelerate the utility of this key sensor modality for sleep monitoring.

## Acknowledgments

This work was partially funded by the National Center for Advancing Translational Sciences of the National Institutes of Health under Award Number UL1TR002378 and the NIMH under Award Number U01MH110925. We also thank to Qiao Li for his assistance in creating table 2.

## 14. Diagnosing obstructive sleep apnea from pulse oximetry

### Jeremy Levy^1,2^ and Joachim A Behar^1^

^1^Faculty of Biomedical Engineering, Technion Institute of Technology, Haifa, Israel

^2^Faculty of Electrical and Computer Engineering, Technion Institute of Technology, Haifa, Israel

### Status

Obstructive sleep apnea (OSA) is a respiratory disease, caused by pharyngeal collapse during sleep and characterized by frequent awakenings. It is a highly prevalent condition with an estimated 425 million (95% CI 399–450) adults worldwide aged 30–69 years (men and women) having moderate to severe OSA, in a review of 17 studies (Benjafield *et al* 2019). Full-night polysomnography (PSG), although time-consuming and expensive, is considered the gold standard for diagnosing OSA. The goal of PSG is to confirm the clinical suspicion of OSA, assess its severity, and guide therapeutic choices. Because of the high prevalence of OSA, the high proportion of undiagnosed individuals and the limited number of sleep labs offering PSG, it has become critical to develop alternative pathways for the diagnosis of OSA from physiological time series recorded by wearable sensors and interpreted by data-driven algorithms. These algorithms have to demonstrate high performance to enable the diagnosis of OSA and be robust to population shifts. The oximetry time series have been used to support PSG interpretation with the typical statistics reported in PSG reports being the 3% oxygen desaturation index, the mean oxygen saturation, lowest value and proportion of time under 90%. Researchers have later elaborated other oximetry features, which are often less intelligible but are designed to better capture the dynamics of the time series or specific events. These include the delta index (Pépin *et al* 1991), power spectral density-based features (Zamarron *et al* 1999) or sample entropy (Richman and Moorman 2000). Recently, research has investigated the combination of such features in a machine learning (ML) model. In addition to using pulse oximetry, some researchers have developed models based on the photoplethysmography signal (Chen *et al* 2021) or pulse rate variability (Blanchard *et al* 2021, Sabil *et al* 2021).

### Current and future challenges

Efforts focused on the analysis of respiratory pathologies based on the oximetry time series have received considerable attention in the last few years. Behar *et al* (2019) developed OxyDOSA, a linear regression model trained on oximetry biomarkers and three clinical features. They trained the model on a PSG clinical database of 887 individuals from a representative population sample of São Paulo (Brazil). They performed a binary classification of non-OSA versus OSA and obtained an AUROC of 0.94 ± 0.02 and a sensitivity of 0.87 ± 0.04 on the test set. Vaquerizo-Villar *et al* (2019) proposed a Convolutional Neural Network (CNN) working on 60 s segments of oximetry. They obtained 93.6% accuracy on detecting apnea or hypopnea events, on a dataset of 453 paediatric patients. However, the majority of studies used a single dataset. One important challenge with translating medial ML algorithms into clinical practice is their lack of generalizability. Indeed, the model must be robust to distribution shifts associated with population sample (e.g. effect of skin colour) or changes in the recording device (i.e. hardware used to acquire the data). As shown by Celi *et al* (2022), generalization is currently one of the main bottlenecks for developing effective clinical ML models. Different sensors exist that can record the oximetry time series. For example, during a PSG test, the photoplethysmogram (PPG) signal (from which SpO_2_ is derived) is recorded via transmission of the light through the finger whereas smartwatches can record the PPG via reflectance of the light through the skin surface. Although the reflectance photoplethysmography signal will often be of a lower quality than the transmission one, smartwatches (or similar wearables) bring the opportunity to ease remote diagnosis of OSA. However, the quality of the physiological measurements obtained using these new wearables is still questionable and there is currently no proof that these may be used for diagnostic purposes (Zhang and Khatami 2022).

### Advances in science and technology to meet challenges

**Figure 19.**
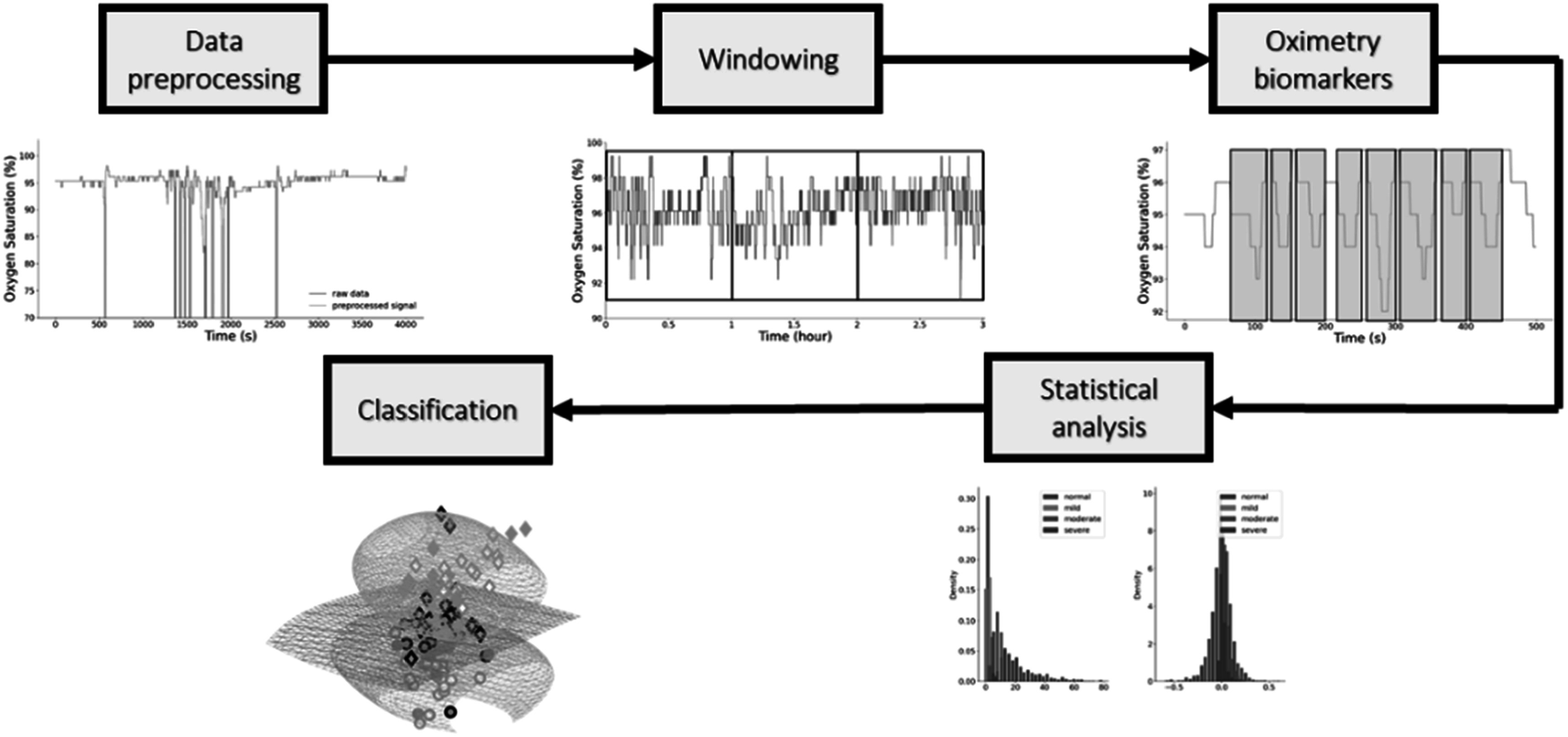
Flowchart for continuous oximetry time series analysis. Source: Adapted from: Behar *et al* ‘Digital oximetry biomarkers for assessing respiratory function: standards of measurement, physiological interpretation, and clinical use’, npj Digital Medicine, 2021, https://doi.org/10.1038/s41746-020-00373-5 (CC BY 4.0).

The oximetry signal can be collected continuously using a pulse oximeter. Levy *et al* (2021b) developed a Python Oximetry BioMarkers (POBM) toolbox to ease the engineering of relevant oximetry features. The oximetry biomarkers (OBM) were divided into five categories: General statistics, Complexity, Periodicity, Desaturation and Hypoxic Burden. Along with the toolbox, a flowchart for continuous oximetry time series analysis, using a feature-engineering data-driven approach has been proposed (figure 19). This can support rigorous research into the diagnosis of respiratory pathologies from single channel oximetry including OSA. This framework, along with a Deep Learning model, has been used in the context of OSA diagnosis by Levy *et al* (2023), for a regression of the AHI. The model developed, called OxiNet, achieved an F1 score of 0.87 on the Sleep Heart Health Study (SHHS) dataset. After a step of transfer learning, generalization of OxiNet has been demonstrated on external datasets from different population samples thus reflecting generalization performance when distribution shifts exist. In another work, Deviaene *et al* (2018) trained a random forest model combining 139 SpO_2_ features and 4 clinical features. The goal was to classify 1 min segments according to whether or not they contained an apnea or hypopnea event. They obtained an average sensitivity of 64.6% on the test set of SHHS for the binary classification where mild, moderate, and severe are confounded, and an averaged accuracy of 87.6%. In the SHHS test set, 73% of the events are hypopneas, which are harder to detect than apnea. That could be one reason for the low sensitivity obtained, compared to the accuracy. Overall, these results motivate the use of a single sensor, the pulse oximeter, to enable low cost and widely available diagnosis of OSA.

### Concluding remarks

There are still challenges in the development of intelligent algorithms for the diagnosis of OSA from the oximetry signal. However, there is strong evidence on the feasibility of OSA diagnosis based on single channel oximetry. There is limited evidence of whether the quality of the oximetry data recorded via wearable sensors is of sufficient quality to enable these data-driven algorithms to perform well enough i.e. enable diagnosis versus screening. The location of the sensor has an impact on the quality of the signal acquisition, and consequently on the performance measures of different algorithms for OSA diagnosis. A study (Hassan *et al* 2021) has shown that the quality of the signal varied at different sensing locations (finger, toe, ear), with several confounders such as ejection fraction or heart failure. Beyond OSA, computational analysis of the continuous oximetry time series has the potential to be used for the purpose of diagnosis and monitoring of several respiratory conditions as demonstrated in the recent work of Levy *et al* (2021a) and Sobel *et al* (2023) on COPD and COVID-19.

## 15. Mental health assessment by autonomic nervous system monitoring

### Raquel Bailón, Spyridon Kontaxis, Pablo Laguna

University of Zaragoza, Centro de Investigación Biomédica en Red

### Status

Mental disorders are recognized among the main causes of the global health-related burden, with an age-standardized prevalence of 12.262 per 100.0000 people in 2019, increasing after the COVID-19 pandemic, and with depression and anxiety being the most common ones (GBD 2019 Mental Disorders Collaborators 2022). Impaired mental health is associated with negative outcomes, such as disability, reduced quality of life, and premature mortality (Matcham *et al* 2019). The diagnosis and follow-up of mental disorders are often reliant on self-reported questionnaires or interviews at specific clinical visits. Consequently, monitoring mental health using wearable technology could play a key role in the early identification of vulnerability, relapses and response to treatment, reducing morbidity, improving patients’ quality of life, and reducing the economic burden on health care systems. In this line, there have been some efforts in the development of digital tools for the assessment of mental health, with most passive solutions being based on actigraphy, speech, sleep and GPS data, and few approaches based on cardiovascular parameters such as arterial stiffness and heart rate (Osipov *et al* 2015, Matcham *et al* 2019, Hickey *et al* 2021).

Prolonged stress is a crucial factor underlying depression and anxiety, with lower stress resilience being associated with higher vulnerability to psychiatric disorders. The physiological response to stress is mediated by the autonomic nervous system (ANS). Repetitive and maladaptive responses to stress might be behind the autonomic imbalance and reduced autonomic reactivity observed in mental health disorders (Kontaxis *et al* 2021). Since ANS regulation of the cardiovascular system impinges characteristic patterns in some physiological signals, such as the electrocardiogram (ECG), and the photoplethysmogram (PPG), wearable devices that can measure these signals non-invasively have great potential for monitoring mental health.

Heart rate variability (HRV), derived from the ECG, is the most widely used and commonly accepted measure of ANS regulation of the heart, mainly in resting conditions. In a recent systematic review HRV was identified as the most useful physiological metric for stress and anxiety detection (Hickey *et al* 2021). Reduced HRV has also been reported in patients with depression (Kemp *et al* 2010). Pulse rate variability (PRV), derived from the PPG, can be used as a surrogate of HRV in many practical situations (see section 5). In fact, PRV has been proven useful to classify mental distress versus calm stages (Zangróniz *et al* 2018), and PRV in response to a mental task has been shown to discriminate major depressive disorder (MDD) patients from controls (Dagdanpurev *et al* 2018), mainly using mean pulse rate, the power in the high frequency (HF) band and the ratio between the power in the low frequency (LF) and HF band (LF/HF).

### Current and future challenges

Despite the potential of HRV for mental health monitoring, there are some unmet challenges. On one hand, since heart rate (HR) dynamics are influenced by changing factors such as respiration, activity, and time of the day, the contextualization of HRV measures is important for their correct interpretation. For example, in Varon *et al* (2019) HRV measures were only capable of distinguishing stress and relax stages when respiratory information was taken into account. On the other hand, in order to be of practical/clinical use, HRV should be obtained during real-life contexts and in a manner which is accepted by the user (considering intrusiveness and discomfort). This is particularly challenging in chronic disorder patients and the reason why wearable devices based on PPG have a great potential in the field of mental health monitoring.

Not only pulse rate but also PPG waveform morphology is altered under stress and depression. In Charlton *et al* (2018) PPG features indicative of stress were investigated in a simulation study in which haemodynamic changes were included in a numerical model to simulate the effect of stress on PPG signals at different sites. The most significant and consistent features were the time from pulse onset to peak, the time from dicrotic notch to pulse end, and the pulse rate. In Khandoker *et al* (2017) multiscale tone-entropy applied to the series of systolic, diastolic and pulse wave amplitudes turned out useful to discriminate MDD patients and controls and to identify those MDD patients with suicidal ideation. Pulse decomposition analysis was used to observe the decreased autonomic reactivity to a stressful stimulus in MDD patients with respect to matched controls, with the percentage of amplitude loss in wave reflections being one of the most discriminating parameters (Kontaxis *et al* 2021).

However, most of these studies used PPG signals recorded at the fingertip by medical or research devices in laboratory settings. The use of wearable PPG to monitor mental health during daily life faces additional challenges that need to be addressed. One of them is the low quality of the PPG signal (Charlton *et al* 2020), especially when recorded at wrist due to its smoother characteristics and higher sensitivity to movement artefacts, which can be aggravated by uncontrolled and improper use of the wearable device (loose or poor contact). Despite the challenges, relax and stress stages were discriminated using PRV metrics derived from a PPG-based wrist-worn using custom-developed wearable (Zangróniz *et al* 2018) or commercially available devices (Beh *et al* 2021).

### Advances in science and technology to meet challenges

One of the most important steps for wearable PPG monitoring during daily-life is the assessment of signal quality. Different PPG features require different signal quality metrics and thresholds. For instance, one PPG signal can have a signal quality sufficiently high for a robust estimation of pulse rate, but not for PRV estimation or pulse wave decomposition. It is also important to discard PPG segments with too low signal quality that may result in outlier features’ estimates. For example, in Beh *et al* (2021) a preprocessing step to remove outlier pulses based on pulse waveform and duration signal quality indices, and a postprocessing step to reject assessments obtained from segments with excessive removed pulses, was proposed to identify mental workload, obtaining a similar performance to one based on ECG with a rate of outcome rejection of around 30%.

The high intersubject variability as well as the sensitivity of HR dynamics to external and internal stimuli require the contextualization of HR-related metrics. Synchronous accelerometer data can be used to identify resting and activity periods (Can *et al* 2019, Cakmak *et al* 2021). Information of different signals (accelerometer, electrodermal activity, respiration) can be fused to increase the capability of PPG metrics to monitor mental health. For example, in Can *et al* (2019) the use of electrodermal activity in combination with PRV metrics, as well as additional context derived from the accelerometer signal, improved stress assessment during real life. In Cakmak *et al* (2021) features from the PPG and accelerometer signals recorded at wrist during eight weeks were used to classify post-trauma symptoms in post-traumatic stress patients. The combination of these passive features with clinical surveys improved the classification accuracy achieved by any of the sources separately. Information from different sources can be combined using machine learning algorithms (Can *et al* 2019, Cakmak *et al* 2021) but other approaches should be investigated which exploit the interactions and relations between the different signals (Osipov *et al* 2015, Varon *et al* 2019).

Different studies have demonstrated the advantages of assessing ANS in response to a stressful stimulus, either mental/cognitive or physical (Osipov *et al* 2015, Kontaxis *et al* 2021), suggesting the convenience of including or identifying stressful stages when monitoring mental health.

In any case, more longitudinal studies are needed where the wearable PPG data are continuously recorded and mental health clinically followed-up for long periods of time (years) in order to allow for the development of personalized models.

### Concluding remarks

In summary, the main challenges that should be addressed when using wearable PPG technology for mental health assessment include: (i) low signal quality with significant data losses during daily life which requires the development of dedicated signal quality algorithms; (ii) the importance of assessing ANS in response to stressful stimuli, rather than just in basal conditions, and the use of subject-specific measures; (iv) the inclusion of contextualization and combination with other sources.

Despite the challenges and limitations of wearable PPG technology for obtaining robust ANS markers during daily-life activities, there is evidence for the potential for this modality to significantly contribute to mental health monitoring, especially when combined with other information such as physical activity, sleep quality, home stay, social interactions, cognitive function and self-reported status (Osipov *et al* 2015, Matcham *et al* 2019).

## Acknowledgments

The work was supported by CIBER in Bioengineering, Biomaterials & Nanomedicne (CIBERBBN) through Instituto de Salud Carlos III, FEDER and Gobierno de Aragon (BSICoS T39-20R).

## 16. Unobtrusive and continuous blood pressure monitoring

### Xiaorong Ding^1^, Nan Ji^2^, Yuan-Ting Zhang^2,3^

^1^School of Life Science and Technology, University of Electronic Science and Technology of China, Chengdu, People’s Republic of China

^2^Hong Kong Center for Cerebrocardiovascular Health Engineering (COCHE) at Hong Kong Science and Technology Park, Hong Kong 999077, People’s Republic of China

^3^Department of Biomedical Engineering, City University of Hong Kong, Hong Kong 999077, People’s Republic of China

### Status

Cardiovascular diseases (CVDs) remain the No. 1 killer over the world, and high blood pressure (BP), aka hypertension, is one of the most important modifiable risk factors of CVDs. Studies have shown that 24 h ambulatory BP, particularly night-time BP, is superior to office BP in predicting total and cardiovascular mortality (Yang *et al* 2019). Tonoarteriography (TAG) (Ding *et al* 2015, Ji *et al* 2021) that can acquire arterial BP waveform continuously, ubiquitously, and unobtrusively is, therefore, very important for pervasive monitoring, early screening, detection, prevention and management of hypertension and its related CVDs.

**Figure 20.**
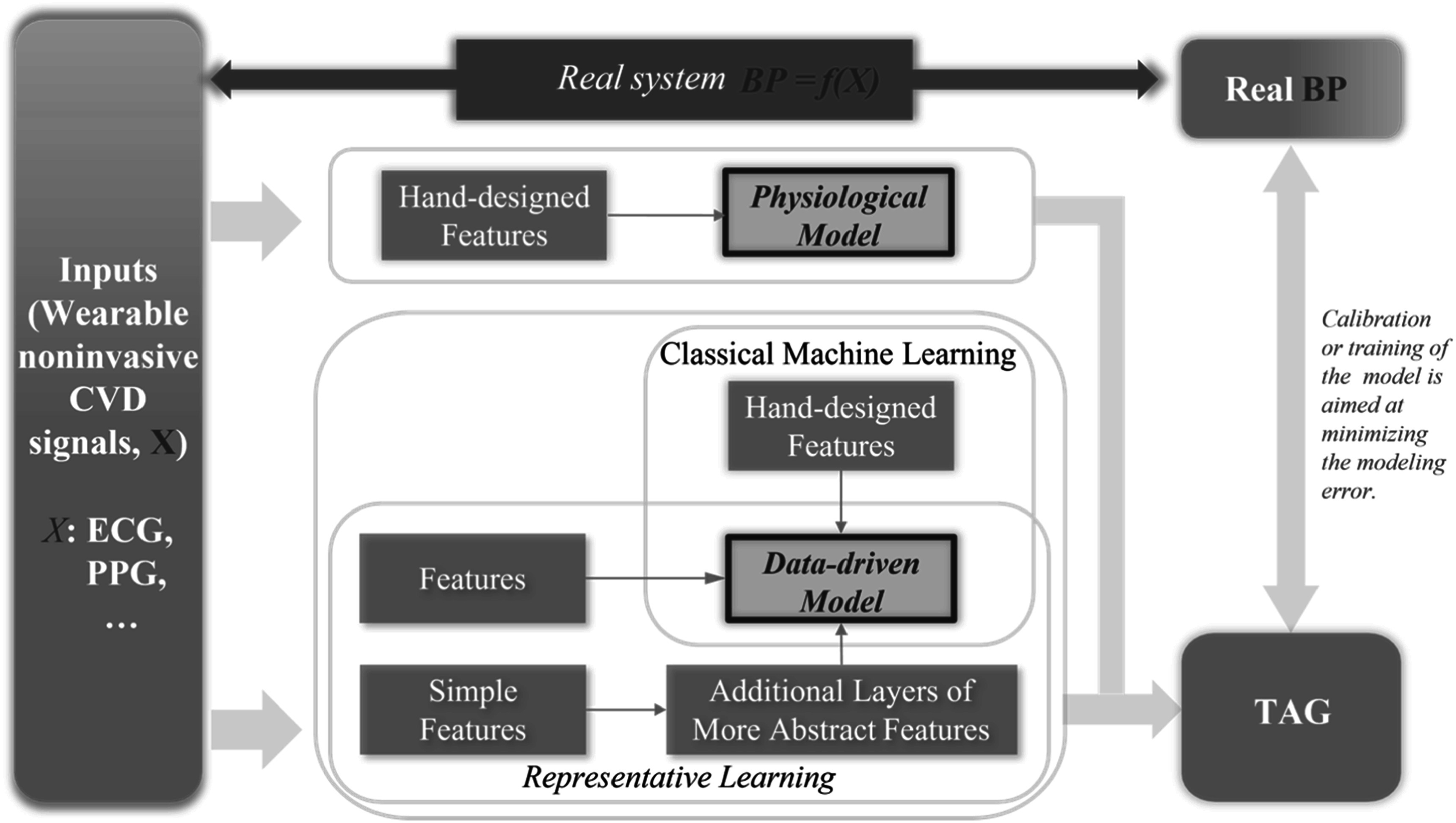
The framework of wearable cuffless blood pressure (BP) or TAG monitoring.

The oscillometric BP monitor is commonly used to measure BP in the clinical setting and at home, but it requires an inflatable cuff that can cause discomfort and disruption to the user and thus is not suitable for monitoring at night. Further, it can only provide an intermittent value of BP. With advances in emerging sensing, computation, algorithm and artificial intelligence (AI), continuous BP waveform via TAG and beat-by-beat BP measurement in a continuous and unobtrusive manner but without a cuff has become possible, with the translation of cardiovascular signal features to BP by either mechanism model or data-driven model (figure 20).

Photoplethysmography (PPG) has been extensively used for cuffless BP measurement, since PPG reflects blood volume change that is related with BP variation. With multiple PPG signals or a PPG signal and other sensing modalities, such as electrocardiogram (ECG), pulse transit time (PTT) can be obtained to indicate BP changes. There have been various studies on PTT-based BP estimation, due to the advantages of the PTT technique being low-cost, ease of use, continuous and noninvasive (Ding and Zhang 2019). The PTT-based BP estimation method relies on the principle of pulse wave velocity (PWV), which relates PWV/PTT with BP. Beyond PTT, there are also other PPG features, e.g. heart rate, PPG intensity ratio (PIR), being used for BP estimation. The BP features are then translated to BP values via a calibration model, which can be mathematical formulas based on the physiological knowledge communicating the BP features with BP, or a data-driven model that maps the BP features or signals to BP (Ding *et al* 2016). Yet, the performance of current studies can hardly meet the international standard for cuffless BP measurement (either TAG or beat-by-beat BP) especially for mid- and long-term monitoring, and advance is desirable to overcome the limitations including the accuracy and stability associated with calibration in the physiological mechanism-based models and the data size in AI-based models for wide application of the technology.

### Current and future challenges

One of the biggest stumbling blocks to cuffless BP monitoring is its limited mid- and long-term accuracy, potentially attributable to four main issues and challenges. First, the precise mechanism underlying cuffless BP measurement is not fully understood. It is clear that specific PPG-derived BP indicators—e.g. PTT/PWV—are associated with BP via the relationship between arterial compliance and pressure. Yet, it remains unclear if the possible mechanism acts in the right way with unrealistic assumptions. The association between unobtrusively obtained BP indicators (e.g. via PPG signal) and BP also seem to vary across a whole range of dimensions, such as age, gender, level of physical activity, mental status, environment, and so on. This emphasizes the importance of research into the mechanism.

Second, the sensing technology that is robust to interruptions and noise—in particular, motion artefacts—determines the practicability of the TAG or cuffless beat-by-beat BP monitoring technique. Signal processing methods to remove the interferences would further enhance the acquired signal quality. The immature sensing technology would result in poor studies in wearable and unobtrusive monitoring system in practice for TAG waveform and beat-by-beat BP estimation.

Another main challenge is the insufficiency of current estimation models. A physiological mechanism-based model is usually based upon a few PPG-derived BP indicators, e.g. PTT/PWV and PIR, and the model is rather simple with limited factors being considered, leading to inadequate estimation accuracy. For a data-driven model, sufficient data that covers various possibility of BP changes, interpretability, as well as personalisation problems are the key issues.

Last but not the least, as the bridge between the research in the lab and the application in practice, clinical validation of the technique requires standard protocol, in which the reference method to measure continuous BP or TAG signal and the duration of measurement are the important factors among others, such as sample size and dynamic range of BP. An accurate reference BP measurement method is the key to evaluate the performance of new methods or devices. However, there are two main issues of the commonly used ground truth measurement methods, e.g. auscultatory or oscillometric method, intra-arterial BP measurement. One is the synchronization problem between the reference BP and estimated BP. Because of the different nature of the reference method and the cuffless BP measurement method and each of them requires one sensing location but BP may vary at different sites of the body, it is very challenging to synchronize them. The other issue is that the reference BP measurement method can be inaccurate. For example, the auscultatory method may be affected by the experience of the observer. Even intra-arterial BP—the gold standard of BP measurement in clinical—can be distorted due to improper placement of the BP sensor and other noises. With regard to the duration of measurement, although there are standards for cuffless BP measurement addressing these issues, e.g. the IEEE Standard for Wearable Cuffless Blood Pressure Measuring Device (IEEE 2019), many validation studies have ignored the important aspects, in particular, the validation of mid to long term accuracy evaluation. Studies that use machine learning models to predict the BP commonly validated BP over a short duration of only up to 6 months (Su *et al* 2018). These showed good performance, but a clear advantage over traditional cuff-based BP measuring method remains to be seen.

### Advances in science and technology to meet challenges

As mentioned above, the biggest dilemma facing cuffless BP measurement is its restricted performance due to the imperfect mechanism, motion artifacts in signal acquisition system, modelling with limited data support, as well as clinical validation standard. Efforts should therefore be undertaken to address these challenges such as to advance the science and technology.

First of all, we need to clarify the mechanism of using unobtrusive CVD signals (e.g. PPG) for TAG signal and beat-by-beat BP estimations. This requires us to understand the relationship between the CVD signals and BP in more depth. More specifically, questions should be elucidated including whether the signals contain the information that represents BP changes, what kind of features can be extracted from the signals to indicate BP changes, and what is the effective model that maps the indicators to systolic and diastolic BPs and TAG waveform.

Next, robust multi-modal sensing systems and signal processing algorithms are required to achieve reliable, unobtrusive and continuous BP measurement. A robust yet neat sensing system is critical to acquire signals for real time monitoring of BP in an unobtrusive manner. Multi-wavelength PPG is such an example for TAG monitoring (see section 4) (Liu *et al* 2018). Further, novel sensor designs and signal processing techniques should be developed to mitigate against noise and interference such as motion artefact. This will promote the development of the technology, commonly confined to the lab, to study in daily use.

Third, we should take advantage of AI techniques to build models to estimate beat-by-beat BP and its continuous waveform—the TAG signal—but with the integration of knowledge and data. For AI-driven methods, it is necessary to share the code for the sake of reproducibility to advance the field similarly to research in computer science. This is further aided by the use of public datasets, since some studies have used the same data but achieved very different results and performance.

Finally, standardisation of the validation protocol is desirable to promote the progress of the technology. The protocol should include population, sample size, validation procedure, dynamic range of BP, and calibration interval. Further, we encourage studies to validate their proposed method with consideration of calibration interval or accuracy duration, since this has been neglected in most current studies. Most important of all, the evaluation standard must be accepted by the clinical community and other stakeholders so as to promote the technology and translate it into practice (Mukkamala *et al* 2021) (see section 21 for further details).

**Figure 21.**
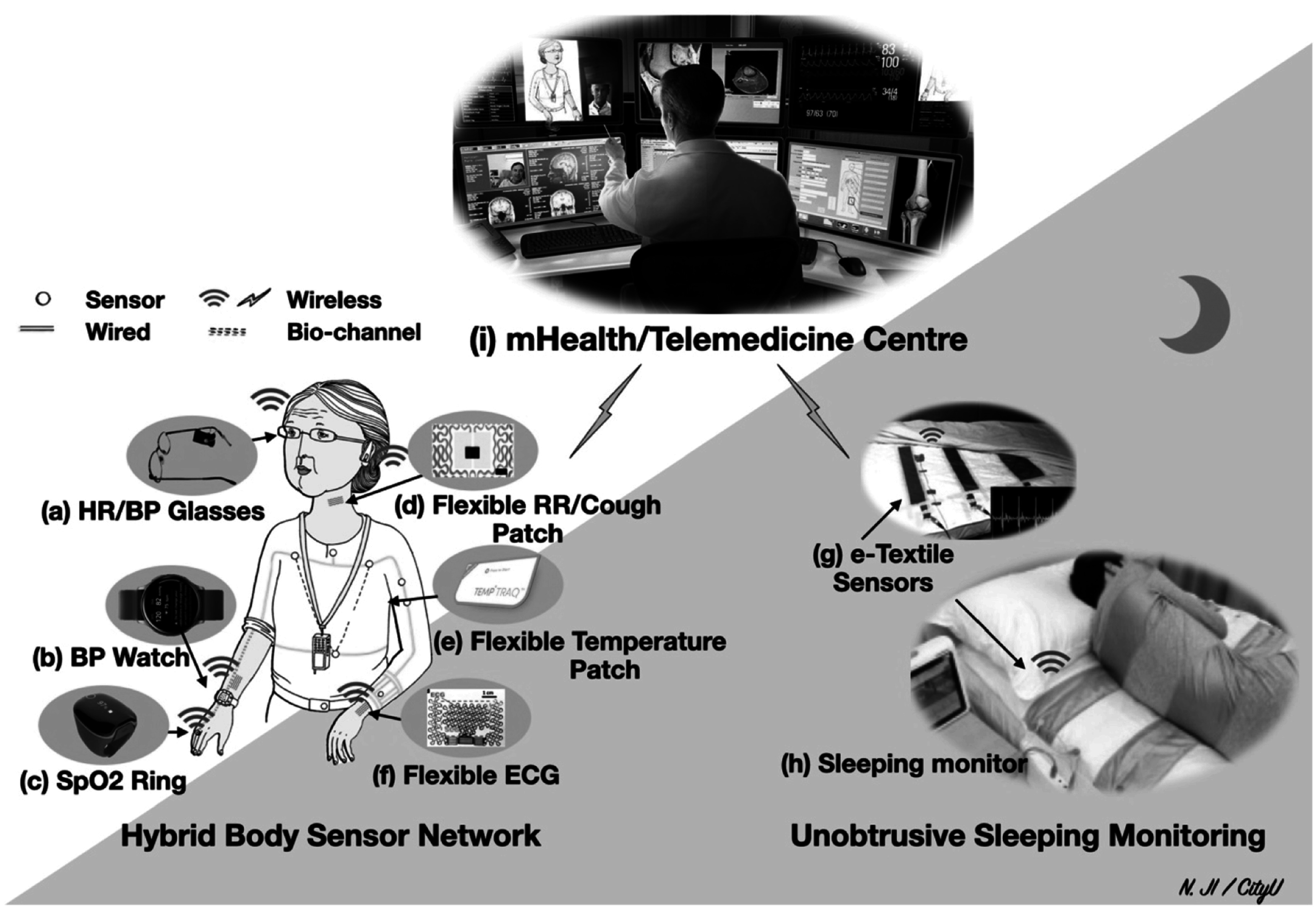
A system for unobtrusive 24 h continuous blood pressure (BP) or TAG monitoring, in addition to multiple other physiological parameters (Ding *et al* 2020).

With the effort to address these challenges, it is possible to advance the TAG technology and make breakthrough against the traditional techniques that have been used for centuries (figure 21).

### Concluding remarks

Wearable PPG technology is a promising way for measuring beat-by-beat BP and continuous BP signals (i.e. TAG) without the inflatable cuff that is commonly required by current BP monitors. However, the imperfect mechanism underlying cuffless BP measurement leads to unsound estimation models. Sensing technology and clinical validation issues further contribute to the limited accuracy of this technology that confines its wide application. As such, it would be very valuable to clarify the mechanism of cuffless BP by investigating the fundamental relationship between wearable PPG and other modal signals with BP, to make the sensing system robust to interference, to build models that can accurately estimate BP, and to standardize the clinical validation protocol, for promoting the TAG technology into practice.

## Acknowledgments

This work is supported by Sichuan Science and Technology Program (2021YFH0179) and InnoHK scheme by ITC.

## 17. Hospital monitoring

### Tingting Zhu, Lei Lu, and David A Clifton

Department of Engineering Science, University of Oxford

### Status

**Figure 22.**
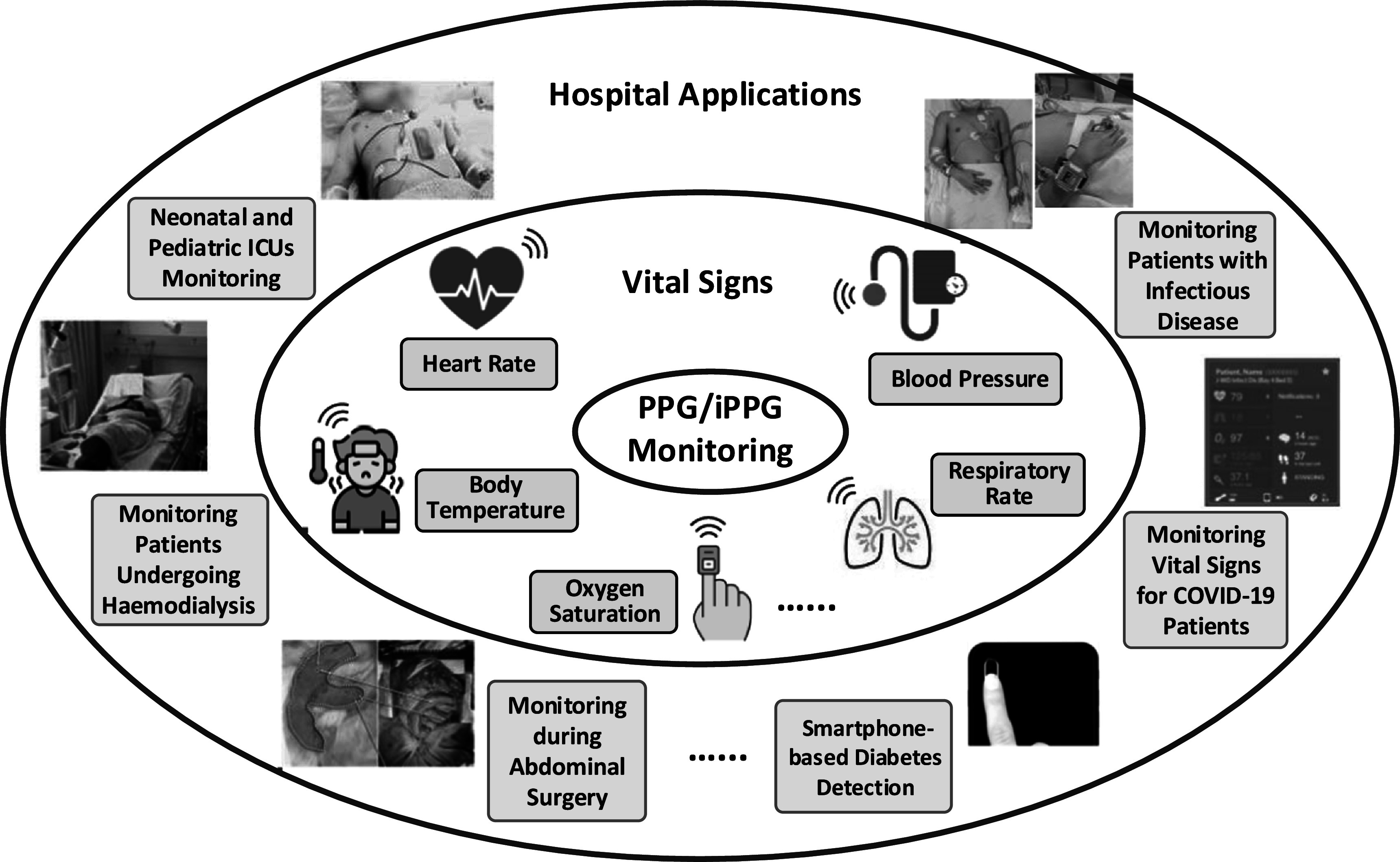
PPG for hospital monitoring. Vital signs include—but are not limited to—heart rate, body temperature, blood pressure, respiratory rate, and oxygen saturation. Applications of PPG-based physiological monitoring include health monitoring in the neonatal and paediatric intensive-care units (Chung *et al* 2020), non-contact monitoring of patients undergoing haemodialysis (Tarassenko *et al* 2014), non-contact monitoring of changes in tissue blood perfusion during abdominal surgery (Kamshilin *et al* 2022), detecting diabetes from smartphone-based vascular signals (Avram *et al* 2020), real-time monitoring vital signs of COVID-19 patients (Santos *et al* 2021), deterioration detection of autonomic nervous system dysfunction in infectious patients (Tadesse *et al* 2020), and others.

A pulse oximeter has long been considered a routine measurement device for patient monitoring in clinical settings (Tarassenko *et al* 2014, Chung *et al* 2020, Kamshilin *et al* 2022). It monitors the oxygen saturation in the blood (via the fingertip or earlobe, for example) and changes in blood volume in the skin using the photoplethysmogram, which measures changes in light absorption in the tissue (Avram *et al* 2020). The photoplethysmography (PPG) waveform is non-invasive and can be collected passively by consumer devices; it is typically available for prolonged periods, and therefore well-suited for in-hospital monitoring to identify transient abnormal events (Santos *et al* 2021). With advances in digital signal processing and machine learning, the PPG waveform has been used for extracting physiological parameters for different clinical applications (Tadesse *et al* 2020, Santos *et al* 2021). Besides the standard vital-sign values (such as heart rate, respiratory rate, body temperature, arterial oxygen saturation, and arterial blood pressure), cardiac parameters (such as electrical heart activity, cardiac output, and heart valve mechanics) can also be derived from PPG (Birrenkott *et al* 2017, Avram *et al* 2020). Some research also focuses on the level of consciousness, mental stress, and pain, based on PPG for patient monitoring (Li *et al* Li 2018). For neurological and metabolic applications, the PPG waveform is used for measuring brain activity, cerebral tissue oxygenation, intracranial pressure, blood gas and blood sugar, and microcirculation monitoring (Lyubashina *et al* 2019, Islam *et al* 2021). Furthermore, recent developments in imaging PPG (iPPG), allow further opportunities for hospital monitoring as iPPG offers non-contact sensing using cameras (Kamshilin *et al* 2022). Current ongoing research for clinical monitoring covers a wide range of applications, including neonatal and paediatrics monitoring (Chung *et al* 2020), diabetes screening (Avram *et al* 2020), hospitalised coronavirus (COVID-19) patient health monitoring (Santos *et al* 2021), predicting deteriorations in patients with infectious disease (Tadesse *et al* 2020), assessment of hypertension and vascular ageing (Shin *et al* 2022), prediction of mortality and hospitalisation of patients (de Souza Kock and Marques 2018), detection of atrial fibrillation and prediction of cardiovascular risks (Aschbacher *et al* 2020), and sleep monitoring and detection of obstructive sleep apnoea (Radha *et al* 2021). Some notable examples are highlighted in figure 22.

### Current and future challenges

Despite the flexibility and low-cost advantages of PPG, its applications in patient monitoring remain limited due to multiple challenges from data collection to disease detection. Most commercially available wearable devices capture PPG in the background, where the signal is unprocessed and noisy depending on the connectivity of the device and the patient’s movement. Vital signs such as oxygen saturation (SpO_2_) can then be calculated using proprietary software. The black-box approach using proprietary software to extract SpO_2_ or other physiological signals makes PPG-based wearable devices unattractive as they are usually not generalisable to all patients with different age groups and diseases. Furthermore, there are currently only a handful of devices on the market that provide access to the PPG waveforms, with the cost of those devices limiting the use of PPG in low-resource settings. Signal quality issues such as motion artefacts and data connectivity, and device battery life, are further barriers to the use of PPG in clinical monitoring. In addition, despite active research into techniques for measuring the signal quality of PPG waveforms, such techniques are mostly tested in healthy subjects (particularly on commercial devices) and on only a small number of participants, which makes it difficult to generalise findings to clinical settings. For long-term monitoring of bed-bound patients, existing PPG-based devices are limited by battery life and Bluetooth connectivity issues.

There is a further question as to whether the collected PPG waveforms contain sufficient physiological information from which vital signs may be estimated. This is a critical criterion as most wearable devices are validated on healthy subjects, and it is required to translate them into clinical practice in which subjects are often co-morbid, elderly, and ill, which confounds existing methods. In spite of recent breakthroughs in using PPG for detecting cardiac arrhythmia, it is limited to atrial fibrillation only. Therefore, PPG research is still at a preliminary stage for describing cardiac morphologies. The new advances in iPPG allow it to serve as a second modality for estimating vital signs information, and make it highly relevant for clinical monitoring due to its convenience in assessment. However, the quality of the signal collected, and the information content provided, limit the use of iPPG. For a clinical environment where highly accurate vital measurements are required for critically ill patients, traditional monitoring systems are preferred over iPPG. Furthermore, iPPG captures videos which can be computationally challenging to analyse, as it requires intensive processing powers and extensive resources for storage.

### Advances in science and technology to meet challenges

The challenges described signpost opportunities for future development. Existing algorithms for assessing PPG signal quality vary according to the device configuration, application, and subjects being monitored (Charlton *et al* 2022). Thus, to extract physiological information from PPG, vital signs-specific quality assessment, such as respiratory signal quality indices (RQIs) (Birrenkott *et al* 2017), in addition to the traditional signal quality indices (SQIs), should be considered in clinical usage. As there is no coherent ‘one model fits all’ solution, Bayesian methods may be used for learning how to optimally fuse multiple independent SQIs and RQIs (Zhu *et al* 2021). Despite the popularity of expert-crafted features from PPG for clinical monitoring, more recent advances in deep learning using neural networks (NNs) have shown promising results. NNs can offer modelling of the PPG waveforms directly, without dedicated algorithms for pre-processing and/or feature extraction. Light-weighted NNs (such as MnasNet (Tadesse *et al* 2020)) are also designed for Internet-of-Things or mobile devices which allow real-time on-sensor processing. However, NNs still lack physiological interpretation of the underlying phenomenon. Detection of different cardiac abnormalities is straightforward in electrocardiogram (ECG) modelling, but is particularly challenging for PPG due to a lack of patient examples. Generative Adversarial Networks can be used as a synthesis tool for generating samples to train algorithms for improved performance (Kiyasseh *et al* 2020). While PPG-based work has previously been used for classifying cardiac abnormality and is validated against those derived from ECG, no work has been undertaken to date on using PPG to perform active sampling to monitor patients’ health status where there is ECG abnormality. Such application would also be extremely beneficial for using wearable devices in clinical settings where existing monitoring can otherwise be either infrequent or entirely absent. This is also beneficial for preserving battery life on wearables and reducing artefacts and transmitting data only when it is necessary. As discussed previously, any algorithms for estimating physiological parameters need to perform sufficiently well and satisfy any intended applications if they are to be deployed effectively in clinical settings. Overall, the clinical utility of PPG needs to be assessed via validated studies in patients to identify suitable algorithms for data extraction and parameter estimation. Further regulatory approvals of PPG-based devices are required for the certification of medical devices to be used for integration into clinical pathways.

### Concluding remarks

This section highlights the status of PPG-based patient monitoring for applications ranging from physiological parameter extraction to health abnormality and disease detection. Although there are ongoing, fundamental challenges in PPG-based wearable devices, advances in science and technology are allowing rich information to be extracted from the PPG waveform, thereby providing exciting potential in patient monitoring, and assisting clinical decision support in hospitals.

## Acknowledgments

The research was supported by the National Institute for Health Research (NIHR) Oxford Biomedical Research Centre (BRC) and by the Hong Kong Centre for Cerebro-cardiovascular Health Engineering (COCHE) funded by the Hong Kong ITC. The views expressed are those of the authors and not necessarily those of InnoHK-ITC. TZ was supported by the Royal Academy of Engineering under the Research Fellowship scheme. DAC acknowledges support from the Oxford Suzhou Centre for Advanced Research (OSCAR). DAC was supported by a Royal Academy of Engineering Research Chair; an NIHR Research Professorship; the NIHR Oxford Biomedical Research Centre; and the InnoHK Hong Kong Centre for Centre for Cerebro-cardiovascular Engineering (COCHE). The views expressed are those of the authors and not necessarily those of the NHS, the NIHR, the Department of Health or ITC.

## 18. PPG low frequency variability and autonomic function

### John Allen^1,2^, Meir Nitzan^3^ and nFei Chen^4^

^1^Research Centre for Intelligent Healthcare, Coventry University, Coventry, United Kingdom

^2^Faculty of Medical Sciences, Newcastle University, Newcastle upon Tyne, United Kingdom

^3^Department of Physics/Electro-Optic Engineering, Lev Academic Center, Jerusalem, Israel

^4^Southern University of Science and Technology, Shenzhen, People’s Republic of China

### Status

The PPG signal is composite in nature with a heartbeat synchronous pulse superimposed on a range of lower frequency components attributed to respiration, vasomotion, autonomic nervous system activity and thermoregulation (Allen 2007, Kyriacou and Allen 2021). Valuable information can be extracted from the PPG in the study impairment of the autonomic nervous system. This is an important area of study as autonomic dysfunction is associated with significant morbidity and can be present in a range of medical conditions, for example in patients with diabetes and types of dementia.

**Figure 23.**
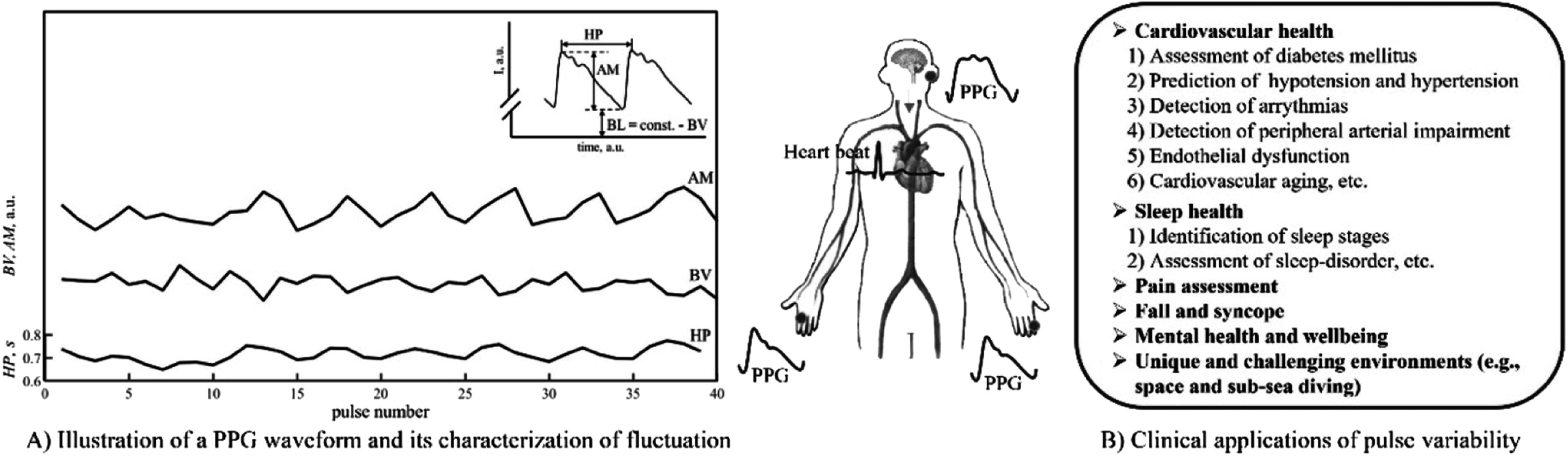
(A) PPG signal showing the variability of the PPG amplitude (AM) and blood volume (BV, or baseline, which relates to the PPG DC measure) and heart period (HP) (see Nitzan *et al* (1999)) for further details), and (B) potential clinical application areas that can utilise the low frequency variability in PPG pulse features—there are many opportunities but more research is needed.

Assessment of PPG variability has included studies of the low frequency pulse amplitude, timing changes and/or morphology changes which are also likely to be linked with autonomic function in humans (Allen 2007, Kyriacou and Allen 2021). Particularly, the baseline and the amplitude of the PPG signal are related to the blood volume and arterial blood volume change, respectively, which are affected by the arterial wall tonus that is controlled by the autonomic nervous system (ANS), specifically the sympathetic nervous system (SNS). The amplitude and the baseline of the PPG signal exhibit spontaneous fluctuations with the same high, low and very low frequencies (HF, LF and VLF, respectively), as those of heart rate (Nitzan *et al* 1994) (figure 23(A)).

In several studies the VLF fluctuations of the PPG amplitude and baseline were studied in the time domain. The VLF variability of the baseline and amplitude showed high correlation between right and left limbs and between fingers and toes of healthy adults (Nitzan *et al* 1998, Khanokh *et al* 2004), which should be attributed to the SNS control. Hence, the correlation between limbs of the VLF PPG fluctuations can be utilised for evaluating SNS function. The correlation was found to be lower in limbs of a number of diabetic patients (Buchs *et al* 2005), probably because of peripheral sympathetic neuropathy. High correlation was also found between the two feet of neonates, including preterm ones, though in some of them the correlation coefficient was smaller, probably due to immaturity of the brainstem or the peripheral nerves (Babchenko *et al* 1999). The between-limbs correlation coefficients and the standard deviations of the amplitude and baseline decreased in patients after sympathectomy and after sympathetic blockade (Babchenko *et al* 2001, Nitzan *et al* 2001), indicating that SNS function can be evaluated by the VLF fluctuations of PPG. Patients with the connective tissue disease, systemic sclerosis (SSc) have also been studied using similar measurement and analysis approaches and the right to left side correlation was reduced with the disease (McKay *et al* 2014).

The wider published literature for this important field in PPG research also includes publications on a range of potential autonomic function applications including the assessment of cardiovascular regulation, diabetic neuropathy, endothelial dysfunction, peripheral vascular impairment, sleep science, pain, falls and syncope (orthostatic hypotension, OH), mental health and wellbeing, and for unique and challenging environments such as micro-gravity/space and military research (figure 23(B)) (Allen and Chen 2021, Allen *et al* 2021). There are also PPG-based autonomic function testing devices on the market including semi-portable systems for measuring beat-to-beat BP and its variability for use in hospital autonomic function testing units and clinical research facilities (e.g. the Portapres/Finometer CNAP type systems for research assessments in heart failure, diabetic neuropathy, and orthostatic hypotension).

Traditionally, PPG studies have been largely limited to non-ambulatory assessments and measurement settings. There is clear scope to make ANS assessments more real world, truly ambulatory, and accessible, by utilising wearable sensing technology (including watches, bracelets, fitness trackers), and validated for use in specific applications. There are clear challenges however to overcome. For example, in order to reliably assess the lower PPG variability frequencies, accurate low-artefact waveforms are needed for feature analysis and quantification of signal dynamics. The lack of standardisation in PPG measurements and analysis is also a concern as it can limit generalisability of a test beyond the centre that it was developed in. Three pressing challenge areas for future research are (i) sensing, (ii) measurements, data and analysis, and (iii) translation to use in clinical applications.

### Current and future challenges

#### PPG sensing

• The optimal body sites for autonomic function assessments are not yet known. Skin PPG measurements are often made at the extremities, but other sites are sometimes needed—e.g. at the forehead which is regulated by both sympathetic (‘SNS’) and parasympathetic (‘PSNS’) branches, and for when scalp access allows, to assess PPG variability there that could be linked to cerebral tissue.
• PPG sensor attachment needs to be reliable. Designs should minimise ‘probe-tissue’ movement artefact, which is very important to help reduce noise, especially if ambulatory measurements are to be performed.
• Technology miniaturisation for portability could include integrating all system components on a wearable sensor itself, but this would not be trivial to achieve.
• Calibration methods are a challenge, e.g. pin-pointing specific SNS function from the measured parameters of the VLF PPG variability.
• The measurements of VLF PPG variability in preterm newborn babies are of utmost importance because early detection of central or peripheral SNS dysfunction enables effective treatment, but their spontaneous limbs movements cannot be controlled. Efficient signal analysis techniques are required to reliably remove patient movement related noise.

#### Measurements, data and analysis

• There are not yet internationally standardised measurement protocols for specific PPG autonomic assessments. Challenges include introducing and getting technologies clinically accepted.
• Developing analysis algorithms is challenging. There will be variability and uncertainty in measurements linking to mental state, i.e. psychophysiological changes in PPG.
• As the heart-beat period and consequently heart rate can be measured by PPG (Nitzan *et al* 1994, Mejía-Mejía *et al* 2020a), heart rate variability can be obtained by PPG instead of using the less convenient ECG.
• Ethical considerations, including global patient monitoring and data use/security.
• There are challenges in differentiating between LF and VLF (as well as ultra low frequency, ULF) PPG fluctuations, as well as extracting the ANS component of HF PPG fluctuations (Bernardi *et al* 1996, Nitzan *et al* 1999). PPG signals are complex and body site dependent and influenced by age and disease. Consequently, one algorithm might not be valid for all sites.
• It is not clear whether analysis should be conducted in frequency or time domains, since each approach has its limitations. Also, short time series analysis to estimate the LF characteristics has known limitations.
• Interpretation of signals and features (e.g. multi-order derivative and multivariate).
• Challenges in developing representative physiological models describing the generation and modulation of the PPG, and its interaction/regulation with other measurements.
• Training in PPG technology and particularly for the study of VLF/ULF is limited.

#### Applications

• Reliably measuring and understanding correlations between VLF PPG fluctuations in two limbs, for the assessment of ANS dysfunction. This approach is important for diabetic patients (peripheral neuropathy), for preterm neonates (central/brainstem immaturity), and for elderly persons (central/brainstem dysfunction). An important question is what the ground truth could be for such assessments.
• Challenges with reliably monitoring ANS during sleep.
• ANS assessment in patients with orthostatic hypotension.
• There are many challenges to extreme environment monitoring, such as space exploration and sub-sea diving.

### Advances in science and technology to meet challenges

#### PPG sensing

• Different body skin sites are differently regulated by the SNS and PSNS. More scientific studies are needed to understand the impact of measurement factors (e.g. body site, wavelength). This could include computational studies of light interaction with tissue.
• Sensor materials, i.e. wearable/flexible technologies must be considered, including skin-inspired organic electronics and skin-interfaced sensors.
• Technology miniaturisation can be made and at low cost.
• Calibration in BP low frequency variability assessments—the next generation ‘cuffless’ BP technologies to be developed.

#### Measurements, data and analysis

• Robust standardised measurement protocols: repeatability and reproducibility data are needed for measurements from wearable sensing solutions.
• To facilitate sharing of PPG datasets collected under various conditions and open-sourced standardised PPG processing code for multi-centre comparative studies.
• To better understand PPG noise, artefact rejection and resilience. This could include AI techniques to facilitate this.
• Differentiation between LF and VLF (and ULF) fluctuations. Explainable AI could have a role in developing and validating different autonomic function tests based on PPG, and to help explain the signals.
• Short time series analysis techniques exist from other science and engineering fields, hence there is scope for multi-disciplinary research and development to improve algorithms enabling reliable biomarker extraction and tracking over time.
• Advances in machine learning techniques including deep learning lends itself to the utilisation of ‘in the cloud’ analysis of the PPG signal.
• Utilise computer simulation software, e.g. Simulink (*Mathworks Inc.*), to develop physiological models describing the generation and modulation of the PPG, and its interaction/regulation with other measurements.
• Advanced analysis and modelling could also be applied to determine the optimal site for autonomic function assessment and thus minimising measurements to just one site for wearable implementation.

#### Applications

**Figure 24.**
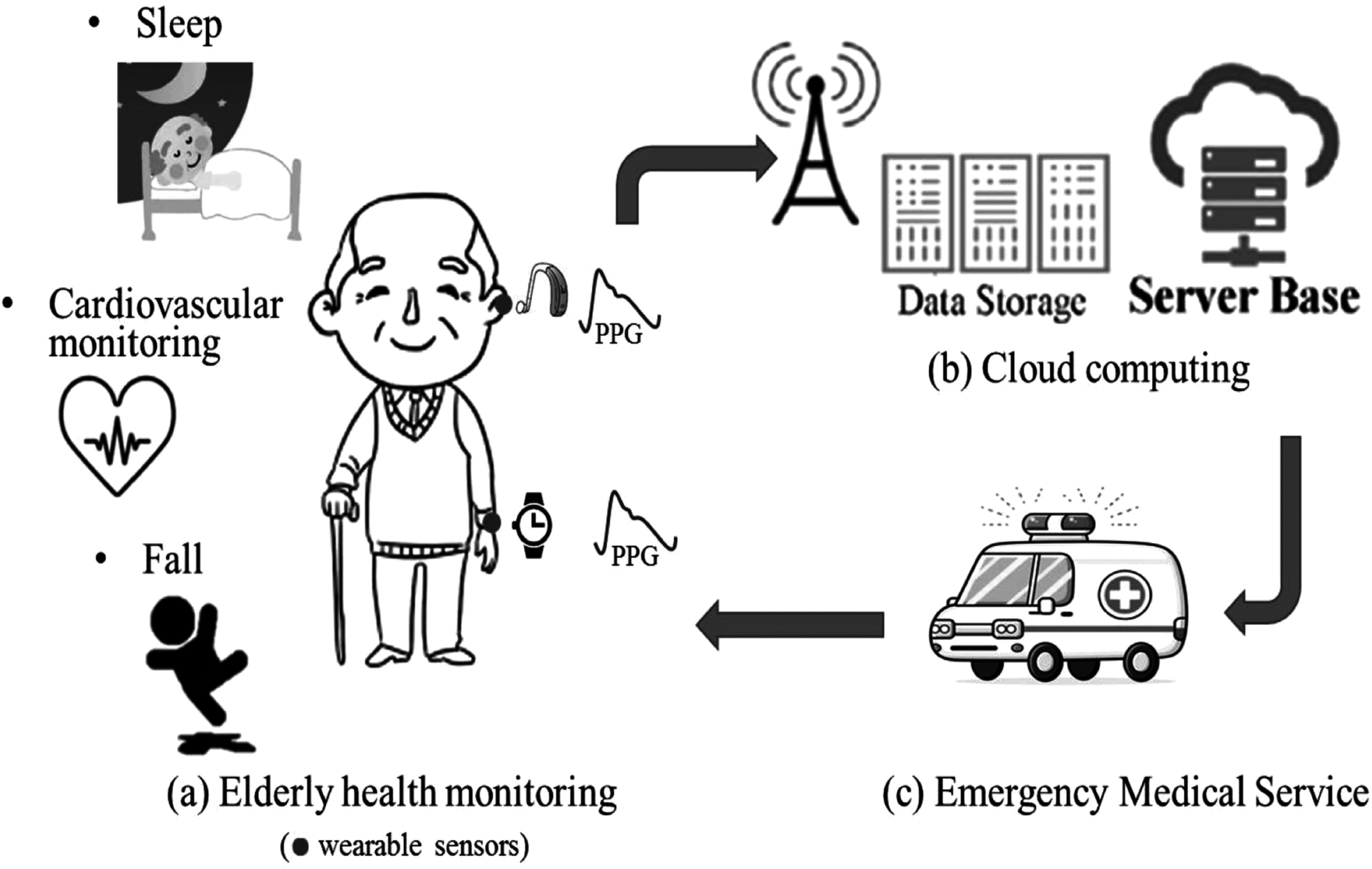
Wearable PPG VLF sensing concept, looking for changes in the signal variability that could be linked to autonomic dysfunction. From PPG sensing in a wearable for a particular application to signal analysis in the cloud of the signals, and with appropriate intervention—in this case an ambulance is called to attend to a patient who has fallen in their home. Ideally, the sensing and signal analysis should have capability to give an early warning of a fall event.

• When measuring the correlation between VLF PPG fluctuations in two limbs, it is essential to determine normal ranges and to quantify the effects of age and gender.
• Developing a reliable technique for a non-invasive measurement of cortical PPG signal variability for the assessment of cortical blood perfusion and its regulation.
• There is much to learn about sleep. Autonomic measurements can be made using a range of techniques including PPG. Clarity on the wide-ranging literature is needed to help determine what information is currently available, what is still needed, and where are the gaps that potential wearable PPG sensing can provide.
• Orthostatic hypotension is usually assessed in Falls and Syncope units in hospitals. Wearable PPG sensors offer the potential for ambulatory real-world monitoring (figure 24), for example utilising cuffless PPG sensing of BP as well as PPG and heart rate variabilities to predict in advance a fall event.
• Population level disease monitoring should be considered, i.e. following on from successful small-scale studies proving the efficacy of PPG lower frequency variability for specific clinical application areas, to then gather big data for global digital health and disease monitoring, e.g. autonomic nervous system by HRV linked features derived from PPG monitoring during a pandemic such as with Covid-19 (Aliani *et al* 2023).

### Concluding remarks

The field of autonomic nervous system LF/VLF/ULF assessments using PPG has been briefly overviewed. The clinical need for further research and development in this area has been highlighted, with much work to be done in PPG measurements made at rest and perhaps before moving to the more challenging case with ambulatory studies using wearable sensing. There is massive potential though for a wide range of clinical application areas in autonomic function and the assessment of low frequency variability, especially with research and development that can be multidisciplinary, involving clinical, science and engineering, technological, and data science teams. There are opportunities emerging for advancements in resilience sensing that covers ambulatory use in the measurement of PPG in real-world settings, including opportunities to utilise the technologies as part of fitness and wellbeing monitoring.

## 19. Assessment of vascular age and arterial compliance

### Chengyu Liu^1^, Yumin Li^1^ and Dingchang Zheng^2^

^1^State Key Laboratory of Bioelectronics, School of Instrument Science and Engineering, Southeast University, Nanjing 210096, People’s Republic of China

^2^Research Centre of Intelligent Healthcare, Coventry University, United Kingdom

### Status

Cardiovascular disease (CVD) is a series of diseases caused by heart and blood vessel lesions and is the leading cause of death worldwide. These deaths are mainly attributed to the long-term effects of atherosclerosis (Visseren *et al* 2021). It is known that the structure and function of blaood vessels continuously degenerate with aging, eventually leading to damage to the brain, heart, and kidneys (Savji *et al* 2013). Vascular function also can decline with poor diet, obesity, smoking, and diabetes. Therefore, conveniently assessing vascular function is essential for the early screening of CVD.

Non-invasive methods to assess vascular function include: ankle-brachial index (ABI), brachial-ankle pulse wave velocity (ba-PWV), and carotid-femoral pulse wave velocity (cf-PWV). In clinical practice, cf-PWV is the gold standard for non-invasive assessing arterial stiffness (Laurent *et al* 2006). When arterial stiffness increases, the adhesion between the fibrous tissues of the arterial wall becomes greater, and the compliance of arteries decreases, which leads to faster PWV. Alternatively, the presence of atherosclerosis can be identified from PWV differences measured between the left and right sides of the body, which has been attempted to assess the vascular age and arterial compliance of a particular individual or even a population group.

However, the above methods are not suitable for wearable monitoring due to their need for bulky measurement devices. In recent years, with the development of wearable healthcare technologies, photoplethysmography (PPG) has provided a convenient method for assessing vascular function. Some researchers have developed technologies to simultaneously collect PPG signals from different body sites to obtain PWV. In addition, PPG waveform features could also be analysed to assess arterial compliance and vascular age. Manufacturers have implemented PPG technologies into different formats of portable devices: smart watches, smart headphones, smartphones, etc (Kusche *et al* 2015, Chan *et al* 2016, Koshy *et al* 2018). Therefore, there is an excellent opportunity to incorporate vascular function assessment into wearable PPG-based devices if sufficiently reliable approaches can be developed.

### Current and future challenges

**Figure 25.**
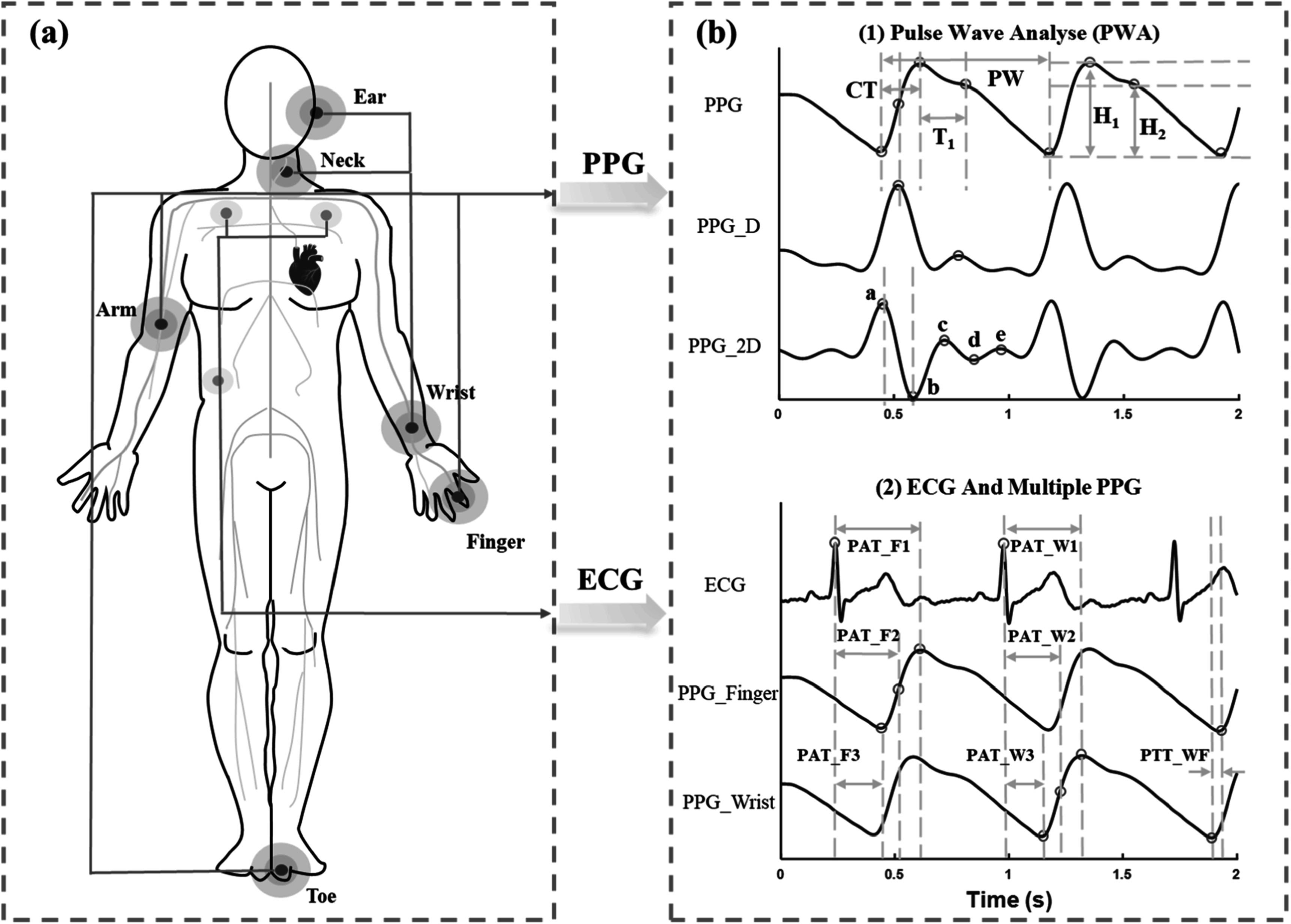
(a) PPG and ECG collection locations. (b) PPG and ECG features for assessing arterial compliance and vascular age. PPG_D is the first derivative of PPG, and PPG_2D is the second derivative of PPG. (1) shows some PWA features. Crest time (CT) is the time delay between PPG valley and peak for calculating the Stiffness index. T1 is the time delay between the PPG peak and the diastolic peak. PW is the pulse width. H1 and H2 are the amplitude for the pulse and diastolic peak. The points a, b, c, d, and e are PPG second derivative features. (2) shows some features (PAT and PTT) between different positions (wrist and finger) of PPG and ECG. PAT_F1 is the time delay between ECG R point and finger PPG Peak. PAT_F2 is the time delay between ECG R point and finger PPG_D Peak. PAT_F3 is the time delay between ECG R point and finger PPG valley. PAT_W1 is the time delay between ECG R point and wrist PPG Peak. PAT_W2 is the time delay between ECG R point and wrist PPG_D Peak. PAT_W3 is the time delay between ECG R point and wrist PPG valley. PTT_WF is the time delay between finger PPG valley and wrist PPG valley.

Although, in recent years, the measurement of PWV has been widely used to evaluate arterial stiffness for clinical research, this approach has not been widely adopted for clinical use. There are currently two main methods for assessing vascular age and arterial compliance by PPG (figure 25):

• PPG pulse waveform analysis (PWA).
• Calculation of pulse transit time (PTT) from the simultaneous acquisition of multiple PPG signals, or pulse arrival time (PAT) from an ECG signal and a PPG signal.

When performing vascular age and arterial compliance analysis by PWA, most studies used time-domain techniques based on PPG signal features (Stiffness index, Reflection index) (Tuktarov *et al* 2020) or PPG second derivative features (von Wowern *et al* 2018). For a comprehensive overview of such features, see Charlton *et al* (2022). Feature parameters obtained from PPG waveforms decomposed using a Gaussian decomposition model have also been studied (Gu *et al* 2014). In order to obtain reliable PPG features, high-quality PPG signals are essential. Many factors affect PPG signal quality, such as sensor wavelength, contact pressure, motion between sensor and tissue, and subject skin pigmentation. However, there is no universal method for assessing PPG signal quality for the assessment of vascular age and arterial compliance. Furthermore, it is important to develop effective algorithms to identify features that reliably and accurately reflect vascular aging and arterial compliance. Finally, after obtaining a series of PPG-based feature parameters, vascular age, and arterial compliance could be assessed by population-based linear regression or deep learning models. To date, the generalisability and robustness of such statistical and deep learning models have not been properly evaluated.

When assessing vascular age and atherosclerosis by PTT, there is no reference standard for PTT calculation. The calculated PTT varies depending on the measurement site where the PPG signal is acquired (wrist, ear, finger, and toe) and measurement posture. Calculating PAT from the ECG and PPG has been widely investigated. However, PAT is the sum of the PTT and pre-ejection period (PEP), making it challenging to obtain a PTT from PAT measurements that truly reflect vascular function changes. Finally, the vast majority of studies have been mainly conducted on healthy individuals, lacking clinical validation against atherosclerosis (Charlton *et al* 2022).

### Advances in science and technology to meet challenges

In response to the above challenges, continuous improvement in science and technology is needed to solve the challenges. First of all, the effect of measurement conditions on PPG-based vascular function assessment should be investigated to standardise the measurement protocol, including the measurement posture, measurement site, measurement duration, etc. Second, more advanced algorithms should be studied to suppress PPG noise and ultimately improve the repeatability of PPG-derived feature extraction. Similarly, the acquisition of other signals (e.g. acceleration signals) could also be used to eliminate the noise. Third, PPG signal quality evaluation standards should be established to determine the classification criteria of different quality PPG signal, and to select the appropriate features for specific applications in assessing vascular age and arterial compliance. Fourth, in order to avoid the effect of PEP on PAT, the ECG could be replaced by other physiological signals (such as the Seismocardiogram (SCG), Ballistocardiogram (BCG), or impedance cardiography (ICG)). Fifth, when assessing vascular age and arterial compliance by regression models or deep learning, separate assessment models can be specifically developed for different sites, postures, and people with different skin pigmentation. Sixth, in order to improve the generalisability, robustness, and evaluation accuracy of the models, a variety of datasets from different populations should be developed to provide enough data for validation. Seventh, the extracted PPG features need to be validated in clinical practice, including in patients with different diseases such as atherosclerosis and diabetes. Finally, for PPG sensors, the relative position of the LED to the photodiode should also be investigated to improve the quality of the PPG signal, which can be used to develop medical devices for assessing vascular age and arterial compliance.

### Concluding remarks

Wearable PPGs are emerging as a potential tool for assessing vascular age and arterial compliance, and more attention has been paid to the use of wearable devices for telemonitoring since the start of the COVID-19 pandemic (Behar *et al* 2020). Much work remains to be done to enable the assessment of vascular age and atherosclerosis by PPG-based wearable devices and their clinical use. Not only do researchers need to overcome challenges with PPG sensor studies, signal processing, and noise suppression, but valid clinical evaluation data and evaluation models are equally indispensable. In addition, there is a need to guide the end user through a simple and standardized process to take measurements. Currently, developing a wearable medical-grade vascular age and arterial compliance assessment device remains a challenge for international organizations and researchers in every country.

## 20. Assessment of peripheral arterial disease

### John Allen^1,2^, Gerard Stansby^2,3^ and Mohamed Elgendi^4^

^1^Research Centre for Intelligent Healthcare, Coventry University, Coventry CV1 5RW, United Kingdom

^2^Faculty of Medical Sciences, Newcastle University, Newcastle upon Tyne NE2 4HH, United Kingdom

^3^Northern Vascular Centre, Freeman Hospital, Newcastle upon Tyne, NE7 7DN, United Kingdom

^4^Biomedical and Mobile Health Technology Laboratory, Department of Health Sciences and Technology, ETH Zurich, 8008, Zurich, Switzerland

### Status

Photoplethysmography (PPG) is a vascular optics technique that can provide composite information about the micro- and macro-circulation (Allen 2007). PPG has many clinical applications, but one important area is the detection of peripheral arterial disease (PAD) due to atherosclerosis (Allen 2021). PAD of increasing severity progressively leads to exercise-induced leg pain (intermittent claudication), and if more severe, potentially to rest pain, gangrene and amputation. The prevalence of PAD increases with age and is also associated with an increased risk of coronary disease and stroke. It is critical to establish a PAD diagnosis in middle aged and older subjects, since other conditions, such as musculoskeletal disease, can mimic the symptoms of PAD (Allen 2021). Screening for PAD with PPG could help ensure the early diagnosis of PAD as well as for follow up and evaluating the efficiency of therapy.

**Figure 26.**
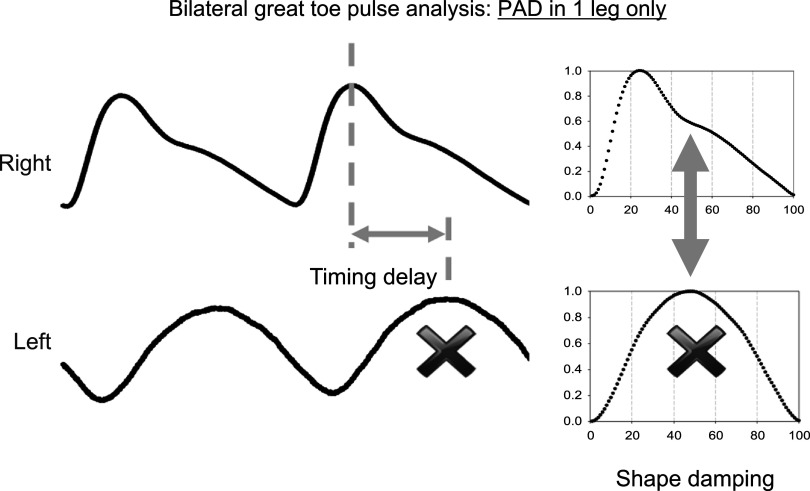
In a healthy subject without PAD, it is expected that there will be bilateral similarity in shape and timing for the great toe pulses, with the typical pulsatile characteristic—as shown for the right foot. In a limb with significant PAD there is usually relative damping and timing delay of the pulse—as shown for the left foot. It is noted that measurements do not necessarily have to be conducted on both legs simultaneously, they could be made on 1 leg at a time—for example for the leg reported to be the most symptomatic. Although PPG is not a perfect test for PAD it has the advantages of speed, low-cost, ease of use, and the potential to improve the accessibility of PAD testing for many people.

PAD usually affects the lower limb arteries (Allen *et al* 2008, Allen 2021). Toe PPG pulses usually become damped and delayed with PAD progression (figure 26), although waveforms can also be similarly affected in the case of microvascular disease/autonomic changes (Bryce *et al* 2022). The reason for the damping and delay of the PPG pulse in patients with occlusive PAD is not fully understood. Although bilateral pulses are shown in the figure to illustrate pulse distortion with PAD it is important to note that measurements do not need to be carried out on both legs simultaneously. It is feasible that sequential measurements could be performed or even measurements only made on the most symptomatic limb.

Standard methods of PAD testing include the Ankle Brachial Pressure Index (ABPI) and/or vascular ultrasound (VU) or imaging with contrast CT or MRI angiography. PPG is not currently routinely used, despite its potential advantages of speed, low cost, and minimal training requirements. PPG PAD technology can be miniaturized, with portable devices being developed for clinical settings such as primary care diagnostics. PPG diagnostics have tended to be restricted to non-ambulatory assessments, which limit test applicability and may potentially miss valuable diagnostic/predictive information about the peripheral circulation.

To our knowledge wearable sensing systems do not currently provide PAD diagnostics but we believe there is a potential role for them. The main current clinical need is for rapid single test in older patients with leg pain, utilising portable equipment suitable for use in primary care and non-hospital environments and for community screening. Wearable sensors, in a shoe or sock for example, could be also be relevant in patients who have undergone interventions, such as bypass grafting or stenting for PAD, to provide monitoring for graft failure or complications and in those with borderline perfusion in the distal limb/foot due to PAD, often in combination with microvascular disease, such as in diabetics, where there may be variation during the day with posture and blood pressure changes and a high risk of foot ulceration or necrosis. Such patients may develop ischaemic foot complications without warning and if high risk could be monitored by a wearable sensor system.

There are three pressing areas for future research in PAD diagnostics with PPG: (i) sensors (e.g. wearable devices), (ii) measurements, data and analysis (e.g. elimination of signal movement artifacts, and the modelling and novel communication of disease to the patient and clinician), and (iii) clinical application of the PPG technologies in PAD. Algorithms need to work reliably when there are co-existent conditions such as diabetes and/or cardiac arrhythmias, such as atrial fibrillation (AF). Test cost acceptability and additional knowledge of the barriers and facilitators of technology adoption could aid the drive toward improving standardization in PPG measurements. Understanding what makes the PPG pulse damped in vascular disease is still not fully understood, but further analytics and modelling including using ambulatory measurements should help boost our knowledge in this area.

### Current and future challenges

#### PPG sensing

• Optimal body sites for PAD detection are not yet known. Measurements are usually made on the skin at the (great) toe pads, but other sites are needed for ambulatory measurements (e.g. at the inguinal and/or popliteal level for pulse transit information and modelling between sites along a limb including between toes and on other toes). There should also be consideration of angiosomes, where different parts of the leg/foot are supplied from different blood vessels, so testing just one toe or one position may not always be sufficient. With all this in mind one would expect consumer devices not to be sufficient and that tailored clinical wearables may be required for PAD assessments.
• The PPG sensor–tissue attachment needs to be reliable, repeatable, and safe, but there is no standardization currently in probe design. There are challenges to miniaturization onto a wearable sensor for portability. A range of potential wearable sensor form factors would need evaluating to home in on the ideal sensor probe design for PAD assessments.
• Cuffless blood pressure (BP) measurement using PPG is a vogue area, with validations so far done for arm (finger and wrist) measurement sites (Hosanee *et al* 2020). It remains difficult to measure the leg BP value from toe PPG or to give an estimate of ABPI using multi-site PPG using cuffless methods.

#### Measurements, data and analysis

• Standardized measurement protocols for PPG PAD assessments are lacking. There are also challenges in developing new protocols that are clinically accepted.
• Training in PPG technology is limited in measurements and analytics.
• Pulse characteristics change with age; this needs to be carefully considered in the choice of normal comparison ranges. There also have been disparities reported with ethnicity (Sinaki *et al* 2022).
• The PPG lower-frequency components at the toe can also provide valuable information for PAD detection (Bentham *et al* 2018, Allen *et al* 2021) but there are issues in interpreting the PPG waveforms and features (e.g. including multivariate) and explaining them.
• There is a need to optimize PAD detection algorithms for milder disease cases which may be clinically asymptomatic.

#### Clinical application of PPG in PAD

• PPG provides composite information on macro- and microcirculations, however, the gold standard test(s) for PAD detection algorithms that also involve a microvascular disease component remain unknown.
• Reliable PAD detection is needed in cases with co-existent diabetes mellitus and/or cardiac arrhythmia.
• Test result communication to the patient and operator also requires further refinement including borderline positive results that may need further investigation.
• A barrier to advancing the technology could come from the lack of understanding of the nature of the PPG pulse and why it becomes damped in vascular disease.
• Algorithm optimization is needed for sensitivity to early disease. Disease severity is likely to be different for different measurement settings, e.g. hospitals, primary care, and home-based assessments, and a ‘real-world’ device would need to account for this (Stansby *et al* 2022).

### Advances in science and technology to meet challenges

#### PPG sensing

• Body site: more studies are needed to understand the impact of measurement factors (e.g. body site, wavelength, and probe-tissue loading).
• Sensor attachment: wearable/flexible technologies for the sensor must be considered, to allow measurement during ambulation which may improve accuracy.
• Technology miniaturization: miniaturization must be possible at a low cost and with ‘lab on a chip’ sensing capability.
• Lower limb ABPI from (great) toe PPG is conceptually possible; this would include using bespoke sensor design and machine learning techniques for cuffless BP’s.

#### Measurements, data and analysis

**Figure 27.**
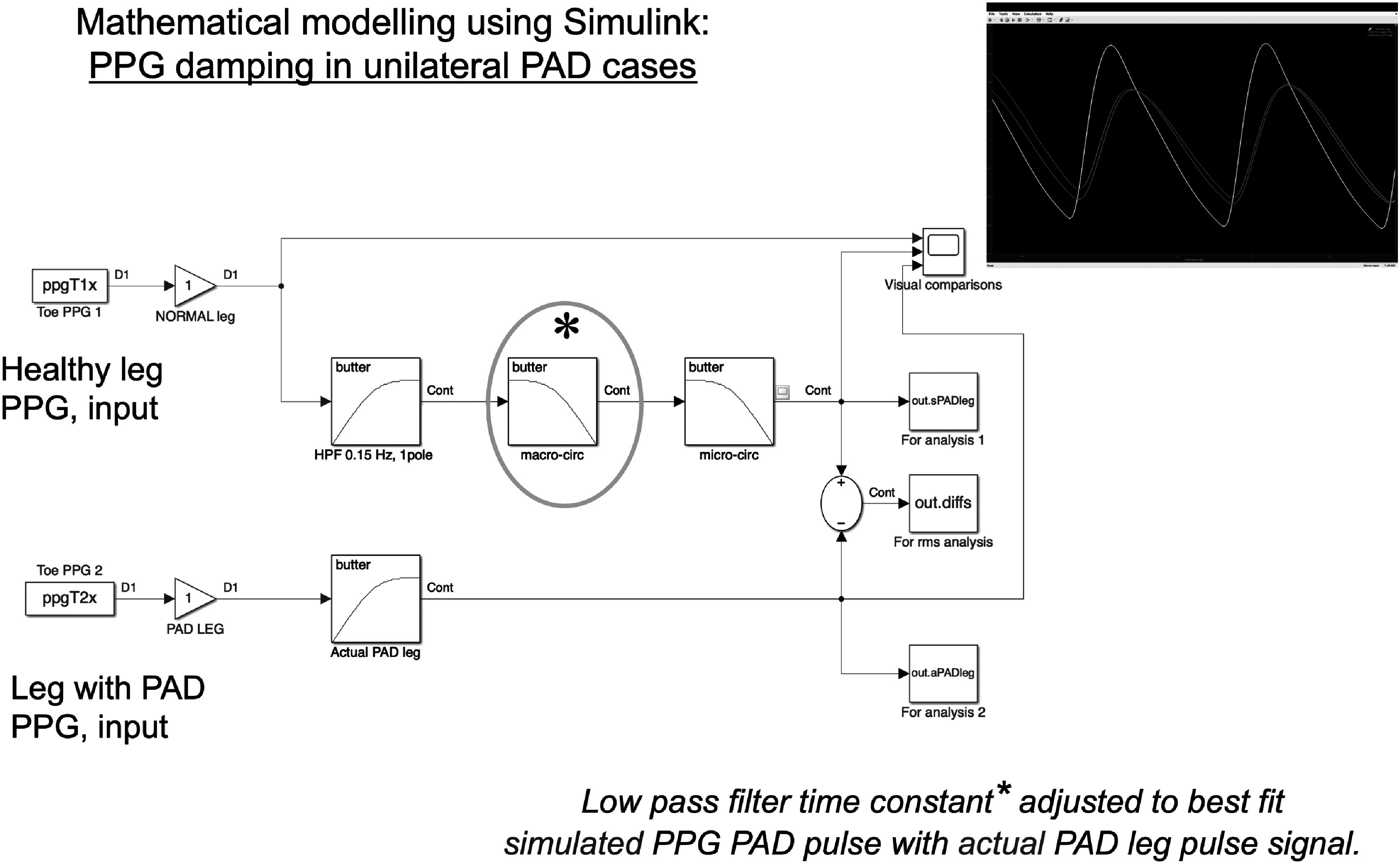
Use of MATLAB’s (*Mathworks Inc*) Simulink software to model simple filter approximations in the study of PPG waveform damping at the toe in lower limb PAD. The main Simulink building blocks and their interconnections are shown just as an overview to illustrate the potential of the approach. Here, a pilot of unilateral PAD cases each had a single pole low pass filter time constant (*) varied to give the best fit (by RMS error) of the simulated PPG PAD pulse with the actual PAD leg PPG signal, and the filter time constants compared with a reference standard for PAD such as the ABPI. Such pilot experiments can form a starting point for better understanding the damping and timing of PPG with disease. More detailed studies could include the analysis of ambulatory/wearable PPG signals for PAD diagnostics.

• Standardized measurement protocols: interlinking sensor design, data analytics, algorithm noise resilience, patient acceptability, and training will be complex. Protocols also need adequate validation and should be co-created with public, patient, technologist, and clinician inputs. Novel disease-detection communication methods should be explored.
• Training in the use of PPG technology is limited. Ideally, measurements should be automated and devices designed to be as resilient and operator-independent as possible.
• Adequate normative data across age, gender, and ethnicity should be collected, and a reliable transferable measurement protocol should be used.
• Optimized noise resilience techniques should be developed and applied to ambulatory measurements.
• Physiological models describing the generation and modulation of PPG and its components, as well as the interaction/regulation between PPG and other measurements, should also be considered (figure 27). It is very important to consider low frequency variability, and measures of this, in such modelling.
• Advances in machine learning techniques, including deep learning and explainable artificial intelligence, are likely to have a significant role in the future in helping to classify and understand PPG signals.
• It is vital that the algorithms are developed and tested for a range of clinical settings (e.g. hospital vascular setting versus primary care settings and in asymptomatic early PAD). It is possible that different PPG features will be best suited to specific clinical settings and disease prevalence.

#### Clinical applications

• Algorithms should be developed with macro- and microvascular diseases in mind, and PPG data sets should be collected using protocols addressing both strands.
• Resilience algorithms need to be developed that can cope with the wide range of patients to be seen.
• Standardization in test protocols is needed for reliable testing, reporting, and interpretation.
• Modelling and simulation could help in our understanding of what actually is being measured and why the pulse often becomes damped and delayed with vascular disease (Tang *et al* 2020).
• Carefully designed research and validation studies should be conducted to gather data from different settings and for different disease prevalence.

### Concluding remarks

The clinical need for further research and development in this area has been highlighted. There are some clear limitations with the current technologies, as well as a lack of understanding of the fundamentals of PPG changes with arterial disease and other clinical reasons. The field of PAD detection is challenging but with the potential capabilities of wearable systems and ambulatory sensing could offer massive potential for a wide range of clinical applications in vascular assessment. With research and development that is multidisciplinary, involving clinical, vascular, technological, and data science teams there are clear opportunities emerging for advancements in resilient sensing that covers ambulatory use in the measurement of PPG and the detection of PAD/vascular disease in real-world settings. There are also opportunities in PAD diagnostics to utilize technologies as part of fitness and wellbeing monitoring.

## RESEARCH DIRECTIONS

## 21. Investigating waveform analysis for blood pressure monitoring

### Ramakrishna Mukkamala

Department of Bioengineering and Department of Anesthesiology and Perioperative Medicine, University of Pittsburgh

### Status

**Figure 28.**
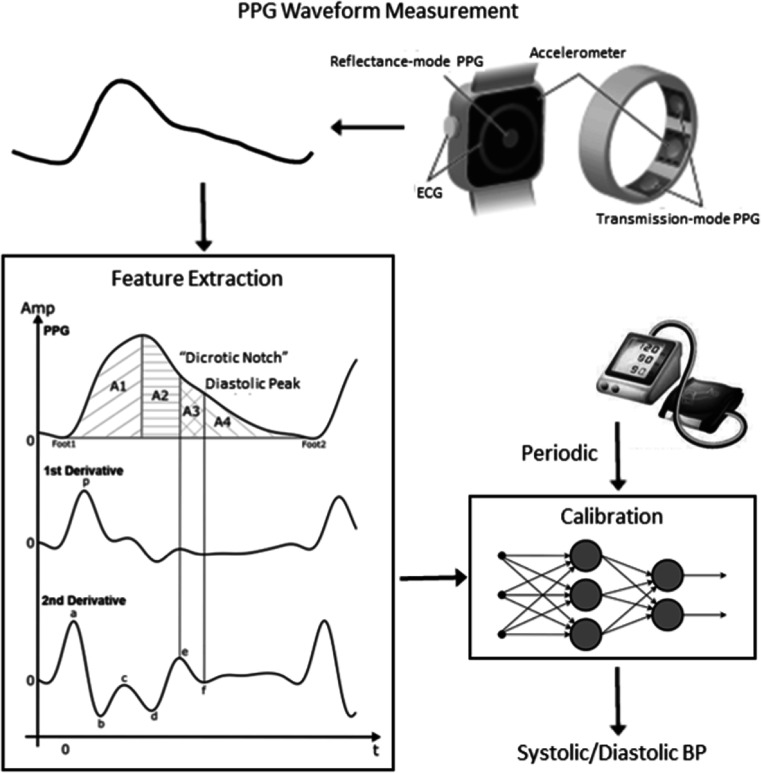
Photoplethysmography (PPG) waveform analysis (PWA) for potentially expanding blood pressure (BP) monitoring. Source: Adapted from Mukkamala *et al* (2022b) (CC BY 4.0).

PWA—photoplethysmography (PPG) waveform analysis—is being pursued by many for blood pressure (BP) monitoring (Mukkamala *et al* 2022a, 2022b) (see section 16). The popularity of this approach stems from recent advances in wearable sensing and machine learning and its potential clinical applications. Figure 28 illustrates the approach. A PPG waveform indicative of blood volume oscillations is measured with a wearable; features are extracted from the waveform; and a calibration model is applied to map the features to BP values. For more accurate calibration, cuff BP measurements are usually required at certain intervals (e.g. monthly). In this way, PWA can seamlessly produce numerous cuffless BP measurements in between the cuff calibrations. The expanded monitoring could reveal the true underlying BP of individuals during daily life including sleep for accurate assessment and effective management of hypertension. It could also enable surveillance and timely treatment of hypotension following major surgery or in intensive care, which is common and likewise a precursor of mortality (Sessler and Saugel 2019).

Studies on PWA for BP monitoring are increasingly appearing in the literature, and cuff-calibrated, cuffless PWA devices are emerging on the market (Mukkamala *et al* 2022a, 2022b). However, there is neither a widely accepted theory nor a convincing body of published, independent data to substantiate the accuracy of this approach (Mukkamala *et al* 2022a, 2022b). As a result, the approach has faced scepticism from longstanding BP researchers and is currently not recommended for clinical use (Stergiou *et al* 2022). Further investigations are needed for practical PWA to have a chance at gaining traction in BP monitoring.

### Current and future challenges

Despite progress, major challenges remain at every stage of the approach.

**Figure 29.**
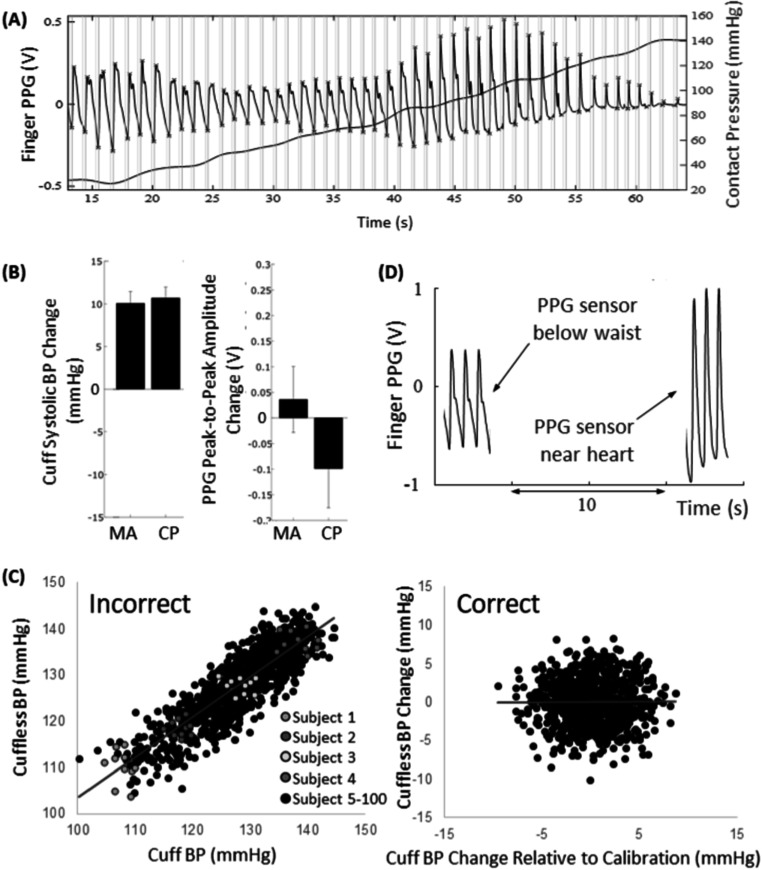
(A) PPG waveform during slowly increasing sensor contact pressure on the finger. (B) Changes in cuff BP and PPG amplitude during MA (mental arithmetic) and CP (cold pressor) tests. (C) Incorrect and correct ways of showing cuffless BP measurement accuracy of PWA with cuff calibration. Because of the cuff calibration, a plot of cuffless BP versus cuff BP pooled over all study participants will largely and trivially reflect the inter-participant differences in the cuff BP levels. A plot of the change in cuffless BP relative to the calibration versus the change in cuff BP relative to the calibration should instead be displayed for a meaningful indication of the BP measurement accuracy. While PWA in this example does not provide any value beyond the cuff BP measurement for calibration, it could in practice. (D) Finger PPG waveform when a tightly applied sensor is at two different vertical heights. Sources: *(B)* adapted from Natarajan *et al* (2022); (C) adapted from Mukkamala *et al* (2021).

Measuring the PPG waveform is more problematic for BP monitoring than arrhythmia analysis, which involves detecting mainly pulse intervals. Firstly, as shown in figure 29(A), the PPG amplitude and shape are sensitive to the PPG sensor contact pressure on the skin in accordance with the well-known oscillometric principle (Natarajan *et al* 2022, Mukkamala *et al* 2022a). Secondly, popular wristbands and smartwatches measure small PPG waveforms from the cutaneous circulation on the backside of the wrist that may preclude noise-robust feature extraction and reflect low-pressure rather than arterial vessels.

Physiological explanations on how the PPG waveform features relate to BP are crucial for facilitating trust in generalizing to inevitable unseen conditions but may be difficult to conceive due to lack of a prevailing theory. Conventional thinking and data suggest that there is no simple relationship. PPG interrogates small arteries that are viscoelastic and abundant in smooth muscle (Mukkamala *et al* 2015). The PPG waveform is therefore a lowpass filtered version of co-located BP oscillations, where the viscoelastic filter gain and corner frequency vary inversely with BP and independently of BP via rapidly contracting smooth muscle. As a result, as shown in figure 29(B), the PPG amplitude can increase or decrease for the same BP increase (Natarajan *et al* 2022). Pulse transit time, which is a cardinal feature and claimed to be detectable from a single PPG waveform (Baruch *et al* 2011), is likewise confounded by smooth muscle contraction for small artery wave travel paths (Mukkamala *et al* 2015). The variability of the viscoelastic filter could also be the reason that several popular PPG waveform features (e.g. diastolic peak) are not consistently discernible (Natarajan *et al* 2022).

Calibration is often considered to be the major challenge in the field of cuffless BP measurement and may be an even greater hurdle for PWA. The reason is that the machine learning-derived calibration model is expected to compromise many person-specific parameters for mapping the features to BP. To determine *N* person-specific model parameters, cuff BP measurements at *N* different BP levels from the person would be needed. Such a personalized calibration model is therefore impractical. The single cuff BP measurement, which is obtained for calibration, only provides a personalized model intercept to set the initial BP level.

Proving that PWA can accurately measure BP at least in controlled conditions is most important but tricky. Because of the cuff calibrations, the sole purpose of this approach is to track BP changes relative to the preceding cuff BP measurement. Interventions must therefore be invoked in human subjects to safely increase and decrease BP via different mechanisms. As shown in figure 29(C), it is imperative to correctly reveal the accuracy in tracking the BP changes above and beyond the initial, cuff BP measurement (Mukkamala *et al* 2021). Standard protocols for validating automatic cuff devices do not involve intra-subject BP changes and therefore cannot be leveraged including their bias and precision error limits of 5 and 8 mmHg (Mukkamala *et al* 2021). Accuracy must also be checked over time to verify the recommended time period for cuff recalibration.

If the PWA device is intended for ambulatory monitoring rather than on-demand measurement wherein it is held at heart level, then hydrostatic effects present a serious obstacle. For example, local BP obtained with a wrist-worn device increases by 7 mmHg for every 10 cm in which the hand is below heart level due to the weight of the blood column (Mukkamala *et al* 2022a). As shown in figure 29(D), the PPG waveform amplitude and shape therefore change when the device is at different vertical heights despite no change in systemic BP. Such changes must be distinguished from similar systemic BP-induced changes.

### Advances in science and technology to meet challenges

Research advances at each stage of this approach are accordingly needed.

An accurate yet convenient PPG sensor should be built. Targeting the finger digital arteries may be reasonable. It would allow for a ring form factor and high-fidelity waveforms even in low signal amplitude conditions (i.e. dark skin, cold) via transmission-mode infrared sensing (Mukkamala *et al* 2022a). Additional sensors should be incorporated to circumvent some of the confounders. For example, force sensing, which is typically not employed, could assess PPG sensor contact pressure variations (e.g. the finger constricts/expands in cold/warm environments), a thermometer could identify temperature-induced PPG waveform changes, and an inertial measurement unit may help detect the vertical height of the device in addition to motion artifact. Other sensors that offer BP information beyond a single PPG waveform (e.g. direct pulse transit time measurement) but do not compromise convenience should also be included.

Rich training databases are vital to create for effective machine learning. A database should comprise measurements of the PPG waveform via the intended device and reference BP from human subjects. The measurements must be available during diverse BP changes for the possibility of discovering generalizable features. Many heterogenous subjects should also be included for the possibility of defining more accurate calibration model parameters that are dependent on basic person information (e.g. demographics). Such a database may be straightforward to establish for the hypotension surveillance application, as many surgical and intensive care patients are hemodynamically unstable and receive invasive BP monitoring for routine care. Note that popular databases (e.g. MIMIC) may not suffice, as the finger clip PPG waveforms therein are heavily processed and likely not representative of PPG waveforms from other sites (Natarajan *et al* 2022). Creating a database for the more popular hypertension monitoring application may require a multi-group, multi-site effort and could leverage natural BP variations during daily life with an ambulatory cuff device as reference and/or proven interventions (e.g. cold pressor, mental arithmetic) (Mukkamala *et al* 2015) plus hypertensive medications with auscultation as reference.

Data-efficient machine learning methods are important to develop even with the availability of a comprehensive database. While a database can include many subjects, it will necessarily be more limited in terms of the critical intra-subject BP variations. Feature dimensionality reduction and other contemporary machine learning tools should be exploited.

Standard protocols must be established to validate PWA devices, especially for the hypertension monitoring application, and one protocol has already been proposed (IEEE 2019). However, further vetting of this protocol remains. Most notably, the protocol does not specify the interventions for changing BP, which could lead to devices that work only in narrow circumstances. Since PWA can offer many measurements over time, which could be averaged to reduce error and intra-person BP variations, even the error limits should be reconsidered (i.e. relaxed) (Mukkamala and Hahn 2018).

### Concluding remarks

PWA could offer great convenience in BP monitoring and may therefore be worthwhile to pursue. However, convenience alone is insufficient. The approach has to work, which means that it has to show significant added value over the cuff BP measurements for calibration. Future investigations will allow conclusive determination of whether optimal PWA implementations are viable for BP monitoring or not. Even if PWA proves unsuccessful, PPG, when combined with other sensing and actuation such as in the oscillometric finger pressing method (Mukkamala *et al* 2022a, 2022b), may still be invaluable for cuffless BP measurement.

## Acknowledgments

This work was supported by the US National Institutes of Health Grant HL146470. The author thanks Dr Anand Chandrasekhar, Dr Cederick Landry, and Mr Mark Freithaler for their contributions to figure 29.

## 22. Sources of inaccuracy in wearable photoplethysmography

### Jessica C Ramella-Roman

Florida International University

### Status

Photoplethysmography can be used as a diagnostic tool for managing cardiovascular disease, provided that high-quality waveforms of sufficient signal-to-noise ratio (SNR) can be obtained from which to extract waveform features. This analysis goes beyond the assessment of pulse peaks, potentially allowing continuous monitoring of several health-related metrics. The photoplethysmogram (PPG) waveform and its derivatives provide a wealth of information on cardiac functionality (Fine *et al* 2021b). Different fiducial points have been used to monitor heart rate (such as the pulse onset, the systolic peak, and the first derivative peak). Additional features have been used to assess cardiovascular function, including: pulse transit time has been used to assess blood pressure; the dicrotic notch and areas under the curve before and after the notch have been used to estimate stroke volume; first derivative parameters have been used to measure blood flow velocity; and higher derivative points for measuring risk for cardiovascular disease (Allen *et al* 2021). Finally, features have been combined using Artificial Intelligence (AI) techniques to characterize blood pressure.

Noise from various sources often impedes precise readings from PPG-based devices, reducing the accuracy of metrics derived from the PPG. Multiple sources of error limit the delivery and collection of light from PPG-based devices, altering the signal. Key factors are motion artifacts, and issues related to the skin-device interface such as sweat and the contact pressure of the sensor to the skin (Jo *et al* 2016, Reddy *et al* 2018, Bent *et al* 2020). Biological variables may also be limiting factors, such as skin tone, age, gender, obesity, and associated physiological differences, such as respiration, body site, temperature, and venous pulsation.

Several studies have characterized the performance of PPG-based devices and observed their limitations through both experimental and computational methods (Bent *et al* 2020, Ajmal *et al* 2021, Boonya-Ananta *et al* 2021); these and future studies are fundamental for instrument optimization and to foster new applications of PPG-based instrumentation. The characterization of errors in photoplethysmography has so far focused on issues related to motion artifact—by testing subjects in various levels of activity—and biological variables—by stratifying subjects by gender, age, race, and, most recently, skin tone (Shcherbina *et al* 2017).

### Current and future challenges

As of today, many continuous PPG-based devices are considered to be ‘general wellness’ devices by the US Food and Drug Administration (FDA), and, as such, do not require regulatory approval. For example, in the United States, currently less than ten among all wearable devices have received clearance for specific functionalities, from the Food and Drug Administration (FDA) (Fine *et al* 2021b). As an example, the FDA has recently granted approval to Apple to track a user’s atrial fibrillation history as part of the Apple Watch electrocardiogram application.

Persistent issues with the accuracy of PPG-derived parameters and regulatory costs limit the transition of most devices to medical-grade labeling. This is particularly true when photoplethysmography is used to ascertain other metrics beyond heart rate.

For example, many research laboratories and companies are working on PPG-based continuous blood pressure (BP) monitoring for the management of hypertension. Accurate and precise measurements, which are paramount when establishing diagnosis and treatment, are largely elusive due to sources of error in the PPG readings. As a result, personalized and repeated calibration of the PPG sensors through alternative systems (e.g. sphygmomanometer) are needed but are inconvenient and costly.

There are contradictory reports on the influence of skin tone on the functionality of wearable photoplethysmography. Many PPG sensors utilize green light, which is highly absorbed by hemoglobin and epidermal melanin, which give the skin its tone. While some studies show significant error in heart rate estimation (Preejith *et al* 2016, Hermand *et al* 2019) associated with skin tone, others indicate that such error is limited (Bent *et al* 2020). Furthermore, research on the influence of skin tone on the shape and features of the PPG waveform is ongoing and not definitive. This is a significant issue, and further studies are necessary to provide equitable access to care.

Obesity changes the skin’s optical properties and thickness, altering the PPG signature (Rodriguez *et al* 2022). Blood flow regulation, capillary density, and skin oxygenation are altered by an increase in Body Mass Index (BMI). Computational efforts (Ajmal *et al* 2021, Boonya-Ananta *et al* 2021) have shown a reduction of up to 60% in signal-to-noise ratio when obesity (BMI > 40) and darker skin tone (e.g. Type 6 Fitzpatrick scale) are combined, as well as PPG features such as the dicrotic notch and diastolic peak being less well defined.

Age is another factor altering the skin and its optical properties, ultimately changing how light travels through the skin. Skin thickness and perfusion decrease with age, and lower capillary recruitment have been noted combined with a reduction in artery compliance. All these parameters modify the PPG waveform amplitude and shape, potentially lowering its diagnostic capability and future applications.

### Advances in science and technology to meet challenges

New studies and alternative approaches to instrumentation are needed to improve the PPG signal-to-noise ratio (SNR) to use photoplethysmography for the characterization of blood pressure and other metrics for the management and prevention of cardiovascular disease.

Further characterization of the error associated with the biological variables listed above is a priority. Specifically, better statistics and stratification of subjects are necessary when addressing skin tone, obesity, age, and gender. To date, our knowledge of the absorption coefficient of the epidermis in populations with elevated skin tone (characterized as Fitzpatrick scale of VI and above) is lacking and based on studies with extremely limited number of individuals (Jacques 2013). Similarly, we still have not experimentally characterized the optical properties of individuals with obesity at different BMI levels. These issues play a fundamental role in optical transport in the skin and consequently in the formation of PPG features. Monte Carlo and other light transport models in the skin have been proposed to devise new instrumentation approaches. Still, they rely on sets of optical properties which are not appropriate for all users. This is a significant issue to be addressed through well-controlled studies of optical properties and full PPG assessment.

Using longer wavelengths beyond the PPG’s standard green light sources could alleviate some issues related to skin variability in tone, thickness, and perfusion. Near-infrared light travels deeper into the tissue as its absorption by melanin and hemoglobin is lower. This, combined with an intelligent selection of source and detector separation, could be used to improve the signal. Similarly, using multiple wavelengths could prove helpful by probing different tissue depths and isolating various skin layer influences (see section 4).

Finally, both the applied pressure on the skin and the location on the body where the measurement is taken show a strong influence on the PPG signal quality and waveform shape (Fine *et al* 2021b), but these factors could be mitigated together with motion artifacts with the addition of pressure sensors and accelerometers and consistent measurement site.

### Concluding remarks

There are many sources of error in current PPG instrumentation that limit the application of this modality to diagnostic metrics beyond heart rate and heart rate variability. Characterizing the PPG features and their association with different biological variables will provide opportunities for both instrument development and future applications. This is considered a crucial future step for this modality.

## Acknowledgments

Dr Ramella-Roman acknowledges the support of the National Science Foundation Engineering Research Center for Precise Advanced Technologies and Health Systems for Underserved Populations (PATHS-UP) (#1648451).

## 23. Wearable data analysis

### Jessilyn Dunn^1,2,3^, Md Mobashir Hasan Shandhi^1^ and Will Ke Wang^1^

^1^Department of Biomedical Engineering, Duke University, Durham, NC, United States of America

^2^Department of Biostatistics & Bioinformatics, Duke University, Durham, NC, United States of America

^3^Duke Clinical Research Institute, Durham, NC, United States of America

### Status

Digital health is a broad scope term encompassing mobile health, wearable devices, health information technology, telehealth and telemedicine, and personalized medicine (Health 2020). Biometric Monitoring Technologies (BioMeTs) are digital health tools that process data captured by mobile sensors and use algorithms to generate measures of behavioral and/or physiological function (Goldsack *et al* 2020). Digital biomarkers are digitally collected data from BioMeTs (e.g. interbeat-intervals from a photoplethysmography (PPG)-based heart rate monitor) that are transformed into indicators of health outcomes (e.g. diabetic state). Digital biomarkers can be used to provide biomedical insights or improve health decision-making (e.g. encourage healthy lifestyle behaviors). Research in digital biomarker development spans fields and disease states, from movement-related disorders to cancer to infectious disease, and can conceivably be applied to any area of health, wellness, and medicine.

There are two levels of data that are generated from BioMeTs: sample-level and processed data. Sample-level data are used as inputs into algorithms that convert that data to a second type of reported data (processed data) that is not a direct representation of the original analog signal. For example, ‘heart rate’ and ‘inter-beat interval’ are two processed data types that can be obtained from sample-level data (e.g. a 32 Hz PPG signal). Processed data are usually calculated through two main steps, where step one involves preprocessing of the sample-level signal and step two involves directly modeling or estimation of the target metric, such as heart rate. The preprocessing step is usually an ensemble of different techniques, including filtering, detrending, transformations, outlier detection and missing value imputations. It is important to note that the processed data are not a direct representation of the original analog signal measured by the sensor; instead, an algorithm was applied to produce that new type of data.

**Figure 30.**
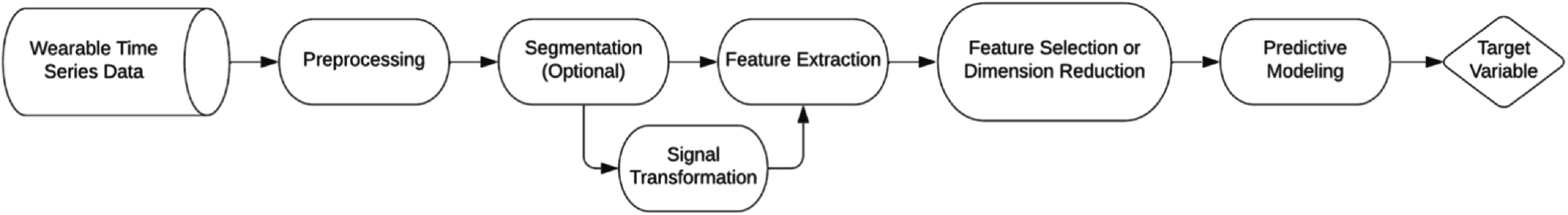
Common predictive modeling pipeline for wearable data.

Digital biomarkers are calculated using BioMeTs prediction algorithms, and these are typically developed through a two-part process of training and testing models to predict a target variable using the sensor data as an input. Common predictive modeling tasks using BioMeT data include regression (continuous target variable) and classification (categorical target variable), with a common pipeline of: (1) preprocessing and segmentation, (2) feature extraction and selection, and (3) predictive model building and testing (figure 30). A common practice before or parallel to predictive modeling is exploratory data analysis and unsupervised learning. Exploratory data analysis can help to uncover the nature and characteristics of the data at hand and how best to handle signal cleaning and artifact removal. Unsupervised learning can reveal hidden structure in the data and can be useful for hypothesis generation.

Most consumer wearable BioMeT manufacturers do not provide sample-level sensor data, but instead provide processed data as aggregate metrics. For example, Apple recently developed a Food and Drug Administration-cleared algorithm for binary (yes/no) detection of atrial fibrillation using the Apple Watch wrist-based electrocardiogram (ECG) and PPG. These aggregate metrics may themselves act as digital biomarkers, or the metrics may be used and combined by researchers and clinicians to develop composite digital biomarkers.

### Current and future challenges

One of the major challenges faced by the research and medical communities is that BioMeT manufacturer-developed algorithms are nearly always proprietary and information about their verification and validation is often not released to the public (Goldsack *et al* 2020). For robust and reproducible digital biomarkers, openness and transparency surrounding the evaluation of these digital tools is critical (Beam *et al* 2020, Bent *et al* 2020, Goldsack *et al* 2020, Bent *et al* 2021b).

Due to the wide range of BioMeTs and heterogeneous phenotypes, ensuring that the details of the predictive modeling pipeline (figure 1) are fit-for-purpose can be challenging. Because BioMeTs collect data in real world settings using different hardware and software, it is difficult to develop a single best practice method for data preprocessing, feature engineering and selection, and predictive modeling (Charlton *et al* 2022). As a result, researchers typically rely on previous literature to decide how best to handle the specific BioMeT data they are working with.

Preprocessing addresses noise and artifacts through detrending, filtering, and signal decomposition. Feature engineering collapses large time series segments into condensed metrics. Using engineered features as inputs into machine learning algorithms generates light-weight and interpretable models that are easy to deploy. Domain knowledge is key to determining how best to select model features. Domain-driven features for PPG may include for example the heart rate variability metrics SDNN and RMSSD, which are known to be related to important physiological processes. However, such domain-driven features do not always exist for every type of BioMeT or physiologic process and also may not fully capture the key characteristics of each signal. Because feature engineering and selection methods must be tailored to each specific domain area, research question, and prediction goal, there is no one optimal method.

In general, predictive models can be parametric or nonparametric and can follow either traditional statistical or machine learning frameworks. Models that are specific to time series data may be used for prediction tasks such as detecting arrhythmias or activity types (e.g. exercise) from PPG and/or movement data. Investigations applying predictive modeling to BioMeT data are far from exhausted. In recent years, deep learning models have become increasingly popular, achieving great performance in many areas of biomedical applications (Marcus 2018, Bock *et al* 2021). As opposed to parametric machine learning models such as logistic regression or linear regression, deep-learning models are an example of non-parametric machine learning models, where the predictive models do not assume a predetermined form of the relationship between the input data and the output labels. Deep-learning models learn only from the data given, and are also considered end-to-end, meaning that the steps of preprocessing and feature engineering are completely automated unless otherwise designed, an extremely desirable trait. However, due to their data-hungry nature, deep-learning models cannot easily learn from small datasets, and available wearable datasets are often considered small in comparison to the large image data repositories like the MNIST database (Lecun *et al* 1998). The process of feature engineering also allows researchers to incorporate domain knowledge into model building, which is difficult to achieve for deep learning models intuitively. More importantly, even though deep-learning models allow researchers and engineers to seemingly skip over the steps of preprocessing, feature engineering and statistical modeling, such models are far from easy to develop. Deep learning model architectures require intelligent design. In addition to building the model architectures using common units such as convolutional layers and long short-term memory networks, researchers and engineers are required to choose whether and how to implement sometimes vaguely understood techniques, such as batch normalization, drop-out layers, and residual networks. Due to their nonparametric nature, deep-learning models can also be extremely large, having to learn and save millions of parameters. This makes the models difficult to deploy onto wearable hardware, which are usually very limited in computation power and storage space. We must be cautious of the promise of deep learning to solve all modeling problems in biomedical applications.

Predictive modeling is also constrained by the limited computation power, battery life and storage space available on current wearable devices. These constraints are most challenging when designing predictive models to be used on wearable systems with immediate biofeedback, demanding the predictive models to be real-time and light-weight while simultaneously providing clinically-acceptable accuracy and sensitivity. A natural progression to the static model deployment strategies is online learning, where the deployed models continue to learn and update iteratively as a result of the arrival of new data. This is especially helpful for developing personalized predictive models, where deployed models can adapt to make more accurate predictions learning from each individual’s data, given the inherent physiological differences among individuals. Even with cloud computing becoming increasingly available and absorbing much of the heavy lifting of computational tasks, it is still a large challenge for current hardware to sustain the energy and storage space required to complete data collection, data storage/transmission, model updates and real-time biofeedback.

### Advances in science and technology to meet challenges

Recent advances in BioMeTs have improved PPG-based measurement of beat-to-beat heart rate, resting heart rate, heart rate variability, respiration, blood oxygen saturation, and more. There is strong evidence of their utility for detecting and monitoring diseases (e.g. arrhythmias, diabetes, influenza, COVID-19) (Dunn *et al* 2021, Goergen *et al* 2022, Shandhi *et al* 2022). Rapid adoption of wearable devices in the general population, with 85% and 21% of Americans owning smartphones and smartwatches, respectively, further increases the potential of these devices to augment the present healthcare ecosystem for remote monitoring of patients and for curbing the spread of infectious diseases. In addition, PPG-based BioMeTs have demonstrated promise in detecting key biomolecules that are important in health (e.g. interstitial glucose, blood glucose, hemoglobin, and glycated hemoglobin) (Dunn *et al* 2021, Bent *et al* 2021a). Despite the promise and potential that PPG-based BioMeTs have demonstrated in health monitoring, there is a lack of fundamental understanding of specific relationships and/or causality among certain diseases and physiologic processes that can be measured through digital biomarkers. For example, heart rate can change due to stress, disease, and other external factors. As a result, algorithms based solely on heart rate monitoring may not be able to differentiate between these conditions, which will result in lesser real-world utility of the developed algorithms. Multimodal BioMeTs with multiple sensor types can gather more physiological information to improve the likelihood of differentiating among similar disease conditions.

It is important to understand and be aware of the limitations of BioMeTs and the factors that can influence the accuracy of their measurements. Alarmingly, PPG-based pulse oximeters have recently been demonstrated to have lower accuracy in people with darker skin due to melanin’s absorption of light (Gottlieb *et al* 2022). To avoid such scenarios in the future, validation studies must include representative populations (Goldsack *et al* 2020). Mechanistic studies must also be performed to understand the underlying measurement mechanisms and isolate and learn how to address potential sources of error.

To avoid the potential of exacerbating existing inequities, technological advancements are needed to decrease the cost of BioMeTs to ensure their equitable distribution so that people who are underserved by our existing health care infrastructure due to social determinants can also benefit from these novel technologies.

Other challenges faced by BioMeTs ​​include a lack of regulatory oversight, limited funding opportunities, cost of computation and storage, lack of standards and validation methods, general mistrust of sharing personal data, and a shortage of open-source data and code. The progress in the field of wearable health data has been further stymied by a lack of cohesion across related research endeavors. Technological advancements in hardware development to miniaturize sensors and to add on-board computation ability and in signal processing to improve algorithms and to compress data to reduce storage requirements and ease of transporting volumes of data between systems can improve the challenges with cost of computation and storage. There have been several notable efforts from academia, industry, and funding agencies to integrate and coordinate efforts in sharing BioM*et al*gorithms and datasets (e.g. the Digital Biomarker Discovery Pipeline (DBDP), Open Wearables Initiative (OWEAR), All of Us research program by NIH) and to standardize digital health data (Open mHealth, IEEE Wearables Working Group). We need to continue and expand our efforts to advance the state-of-the-art of BioMeTs and digital biomarker discovery, including the adoption of benchmark datasets for algorithm comparison, and to bridge gaps in evaluation and applications across academia, government, and industry to establish mobile and digital health as an evidence-based field worthy of our trust.

### Concluding remarks

The accessibility of mobile and wearable technology affords an unprecedented opportunity to provide healthcare globally, conveniently, and to populations with limited healthcare accessibility such as low-income and rural populations. Mobile and digital health monitoring and interventions are promising because they can improve the health monitoring of patients who are unable to make frequent visits to a healthcare facility. Tools that allow for collaboration in improving algorithms, validating known digital biomarkers, and discovering new digital biomarkers will enable much-needed standardization and interoperability in this space.

## 24. Understanding the origins of the photoplethysmogram

### Panicos A Kyriacou

Research Centre for Biomedical Engineering, City, University of London, London EC1V0HB, United Kingdom

### Status

Photoplethysmography (PPG) is a non-invasive optical technique widely used for studying and monitoring the pulsations associated with changes in blood volume in a peripheral vascular bed (Kyriacou and Allen 2021). PPG is well-known for its well-established application in pulse oximetry, used for the continuous and non-invasive measurement of arterial blood oxygen saturation (SpO_2_). Over the past few decades, there has been a plethora of research in the field of PPG with potential applications beyond pulse oximetry, especially with the recent growth of wearable technologies utilising the technique of PPG. Despite the widespread use and acceptability of PPG, still the origin of the PPG signal has been the subject of continuing discussion and debate (Challoner and Ramsay 1974, Mannheimer *et al* 2007, Kamshilin *et al* 2015, Sidorov *et al* 2016, Moço *et al* 2018, Chatterjee *et al* 2020, Kyriacou and Chatterjee 2021).

During the 1980s, there was a plethora of research on the fundamental questions related to the origin of the PPG. After gaining a rudimentary understanding of PPG, the research mainly focussed on technological developments of PPG sensors and signal analysis techniques to extract various physiological information from the PPG. Also, the overwhelming acceptance of pulse oximeters in both the clinical and home settings in a way diminished and somewhat overshadowed progress towards further fundamental PPG research. Such research has regained momentum in recent years, prompted by research aiming to extend the application of PPG beyond pulse oximetry, especially for PPG-based wearable technologies. Hence the simple question was raised again: ‘Where is the PPG signal coming from and what does it represent?’

With the advancement in computational modelling and imaging technology, it has been possible to look deeper into the light-tissue interactions associated with PPG and perhaps to contribute further to the knowledge relating to the origin of the PPG, beyond what has been reported in the literature in the past decades. A wide spectrum of research has been carried out to investigate PPG in relation to; changes in haemodynamics, vascular mechanics and hemorheology; contributions of various absorbers and scatterers present in blood and tissue-layers; the effect of the tissue-anatomy and sensor location; the influence of pulsatile blood flow; the selection of optical wavelengths, and much more (Reuss 2005, Mannheimer 2007, Kamshilin *et al* 2015, Sidorov *et al* 2016, Moço *et al* 2018, Chatterjee *et al* 2020).

### Current and future challenges

Photoplethysmography utilises the absorptivity of light resulting from the variations in the physiological properties of the tissue components during the cardiac cycle. During systole, blood pumped out of the heart rushes throughout the body, including all the peripheral tissue sites. This systolic increase in blood volume results in increased absorbance of light in tissue compared to the diastolic state. The PPG waveform is formed by the unabsorbed light detected by the optical sensor (photodiode). In general terms, this relative change in light absorbance gives rise to the PPG pulsatile waveform synchronous with each heartbeat (Kyriacou and Chatterjee 2021).

Research into the origin of the PPG over the years has so far identified three main contributing factors relating to the origin of the PPG signal: (a) the red blood cell (RBC) orientation and deformation, (b) the volumetric distribution of the absorbers and blood volume variations, and (c) the mechanical movements of the capillariess.

Some of the initial findings in this subject suggested that the changes in red blood cell orientation with the cardiac cycle are the leading causes of forming a PPG wave. This hypothesis is based on the electrophysiological characteristics, where at the end of diastole (i.e. low blood flow), the red blood cells orient themselves randomly due to reduced shear stress; as blood flow increases, the red blood cells tend to align themselves along with the flow; and during systole, the alignment is parallel to the direction of the flow (Kyriacou and Chatterjee 2021). Previously, in-vitro and ex-vivo experiments were carried out by several research groups (Hertzman 1938, Challoner and Ramsay 1974) who also concluded that blood cell orientation and deformation have a potential role in the origin of the PPG signal. Additionally, this hypothesis is supported by the experimental observation by Lindberg and Oberg suggesting that the light transmission and reflection from an artificial blood vessel with continuous blood flow are dependent on blood volume changes and orientation as well as the deformability of the red blood cells (Lindberg and Oberg 1993). A similar observation has been reported from a more recent investigation by Shvartsman and Fine who simulated pulsatile blood flow and confirmed that PPG-like signals are associated with geometric changes in red blood cell aggregation (Shvartsman and Fine 2003). All these experimental verifications confirm the contribution of the red blood cell orientation/deformation, however, whether this is the only source of the PPG signal or not remains a question.

### Advances in science and technology to meet challenges

In recent years researchers utilised the volumetric model for investigating the origin of the PPG (Chatterjee *et al* 2020). The PPG signals originate from the pulsatile arterioles that branch from the upper blood net dermis, having a maximum density in the reticular dermis. The pulsatile vessel movement corresponding to the cardiac cycle results in periodic variations in the volumetric distribution of blood. Consequently, the optical absorbance in the vascular tissue bed periodically changes, forming the PPG signal. This supports the blood volume variation (BVV) model presented by Moco *et al* (2018) who validated the hypothesis through Monte Carlo simulations followed by experimental verifications using diffuse reflectance spectroscopy and videocapillaroscopy. Their investigation concluded that the PPG is formed due to the absorbance of light through the dermal arterioles, however, they still did not eliminate the possibility of co-occurrence of another source contributing to the PPG signal formation.

Blue and green light can also produce detectable PPG signals even though these wavelengths do not reach the pulsatile arterioles (Chatterjee *et al* 2020). The origin of green PPG seems to support the hypothesis by Kamshilin *et al* that the PPG formation is due to the modulation of the blood volume in the capillary bed due to the mechanical movements of capillaries (Kamshilin *et al* 2015, Sidorov *et al* 2016). More recently, the modulation in the optical properties of non-vascularised epidermis corresponding to the heart rate has also been reported by Martinelli (Martinelli *et al* 2019) where they suggest that the mechanical changes in the arterioles and capillaries cumulatively induce changes in the epidermis mechanical properties. Though a direct investigation regarding the effect of such epidermal changes on PPG has not been carried out yet, it is likely to be one of the factors affecting the PPG shape and hence its contribution to the origin of the PPG. Green light is widely used in many werables devices, especially those focusing on heart rate estimation, due to its high absorptivity for both deoxyhaemoglobin and oxyhemoglobin (Van Kampen and Zijlstra 1965). In addition green PPGs have been found to be more resistant to motion artefact, yielding high Signal-to-Noise (SNR) PPGs (Sun and Thakor 2016).

In order to investigate the origin of the PPG more comprehensively, it is crucial to have a profound understanding of the underlying light-tissue interactions. Investigating PPG light-tissue interactions has been challenging due to the limitations in the traditional analytical models. Diffusion approximation is used for analysing the light-tissue interactions in near-infrared spectroscopy, photoacoustic tomography, and optical coherence tomography, however, it fails to produce accurate results in small source-detector separations, a geometry usually used in PPG probes (Schmitt 1991). With the advancement of computational techniques, it has been now possible to explore Monte Carlo models for simulating light-tissue interactions in PPG (Reuss 2005, Chatterjee *et al* 2020). The comprehensive analysis of variables such as optical pathlength, depth of penetration and absorbance could lead to a more qualitative and quantitative assessment of the PPG origin.

### Concluding remarks

Understanding the origins of the PPG signal is an ongoing endeavour, and it will continue to challenge many researchers. Based on the investigations to date, it is inferred that all three of the hypotheses relating to the origin of the PPG, i.e. blood cell orientation, blood volume variations and mechanical movements of capillaries, indeed contribute towards the formation of the PPG signal. The current significant increase in the applications of PPG technologies and their potential contributions in applications in healthcare and wellbeing has motivated many researchers to revisit the challenging question, of ‘where does the PPG signal come from?’ The more we know about the origin of the PPG the more we can ‘unlock’ its relationship with various pathophysiological phenomena. Such new knowledge can lead to disruptive non-invasive technologies for monitoring in healthcare and wellbeing beyond the current state of the art.

## 25. Alternatives to photoplethysmography

### Carmen C Y Poon^1^ and Peter H Charlton^2^

^1^GMed IT

^2^Department of Public Health and Primary Care, University of Cambridge, Cambridge, United Kingdom

### Status

Photoplethysmography (PPG) is an optical technique that can detect blood volume changes in the microvascular tissue bed. By using a pair of light emitting diode (LED) and detector, the pulsation of blood at a peripheral site (e.g. the fingertip, toe, ear lobe, forehead) can be measured, digitized and displayed in details.

#### Brief history and milestones

**Figure 31.**
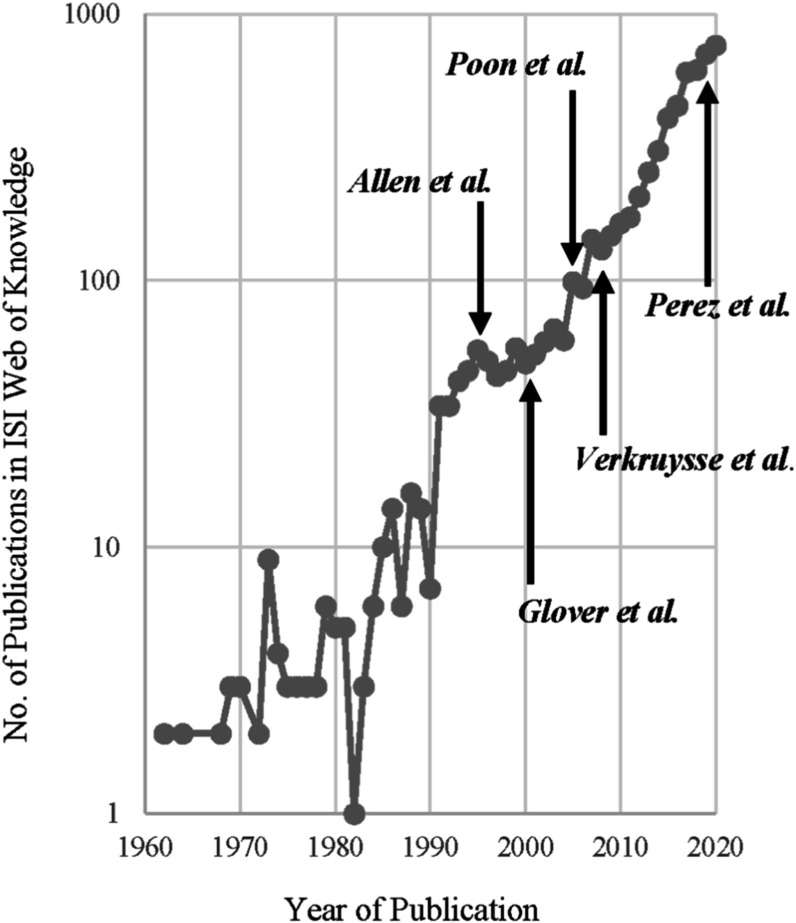
The number of publications in photoplethysmography and selected highly cited works in this field. A wide range of topics in photoplethysmography have been documented, for example, (a) the assessment of an artificial neural-network for the detection of peripheral vascular-disease from lower-limb pulse wave-forms (Allen and Murray 1995); (b) the correction of physiological motion effects in fMRI (Glover *et al* 2000); (c) the concept of a biometric security scheme in telemedicine and mobile health (Poon *et al* 2006); (d) the remote plethysmographic imaging using ambient light (Verkruysse *et al* 2008); and (e) the large-scale assessment of a smartwatch using consumer PPG-based wearables to identify atrial fibrillation (Perez *et al* 2019).

The research on PPG can be dated back to the 1930s (Allen 2007); however, the field did not gain a lot of momentum until mid-1990s (see figure 31), when PPG pulses obtained from the subjects’ toe were first classified by artificial intelligence for detecting peripheral vascular disease (Allen and Murray 1995). Since then, the applications of PPG have widened, for example, to correct physiological motion effects in functional magnetic resonance imaging (fMRI) (Glover *et al* 2000) and to build a biometric-based security scheme for sensor network in telemedicine and mobile health (Poon *et al* 2006). Furthermore, when fusing information from multiple LEDs and sensors at different wavelengths (i.e. multi-wavelength PPG), or other cardiovascular signals such as electrocardiogram, or ballistocardiogram, a spectrum of vital signs related to the vascular system can be estimated. These include but are not limited to the blood oxygen level, blood loss, respiratory rate, depth of anaesthesia during surgery, and even the cuff-less measurement of blood pressure (Poon and Zhang 2005). More recently, a large-scale assessment of a smartwatch to identify atrial fibrillation was also reported, in which 2161 subjects were screened out of 419 297 participants using consumer PPG-based wearables and subsequently asked to wear an electrocardiography patch for 7 d to confirm the presence or absence of atrial fibrillation (Perez *et al* 2019).

### Current and future challenges

#### Understanding the vasculature and its biology using PPG and other alternatives

Although PPG has been proposed independently to measure blood flow, blood pressure, and blood content analysis, these physical and biochemical quantities in fact represent different aspects of a vascular system. PPG, even with multi-wavelength analysis, may not be able to reflect the entire complex mix-up of the peripheral vasculature. In many cases, the measurement of one parameter is based on the assumption that many other parameters remain constant during the measurement period. This is an assumption that often holds valid only when the biological system is under certain stable conditions, but may be weakened when the body is in a disease state or during acute and transient phases. Understanding the vasculature using PPG is a big research challenge, as the vasculature involves not only the mechanical and physical properties of the vessels that give the pulsatile blood volume changes captured by PPG, but also many other components. For example, some other important aspects that must be considered include: the electrolyte analysis of the salts and minerals found in blood; the electrical impulses generated and communicated with other organ systems; and the protein-rich fluid that transport back and forth the circulatory system and lymphatic vessels to serve the important immune surveillance function. Alternatives to PPG can therefore provide different angles to understand the vasculature and its biology, for example, to understand systemic atherosclerotic disease (Geng *et al* 2022).

#### Limitations of wearable PPG with respect to other alternatives

Although PPG has been extensively used in clinical settings, its utility in daily life is yet to be seen. Several big research issues need to be solved before wearable PPG can be widely used in our daily lives. First, PPG signals are susceptible to motion artefacts, especially when used during exercise, in a free living condition, or in an environment where the ambient light varies. Second, PPG sensors often need to be firmly attached to the human body at a constant pressure in order to ensure good signal quality. The design becomes challenging when long-term measurements across days or large-scale population-wide screening using PPG in a crowded area are needed, where sometimes other non-contact RGB camera-based imaging techniques are preferred (Verkruysse *et al* 2008, Sun and Thakor 2016). Third, PPG is often considered as a power-hungry sensing technique compared to other sensing methods. Therefore, an energy-efficient sampling technique is needed, especially when a wearable device designed with PPG is to be used together with other ambulatory devices over a 24 h period or longer (Zheng *et al* 2016).

### Advances in science and technology to meet challenges

**Figure 32.**
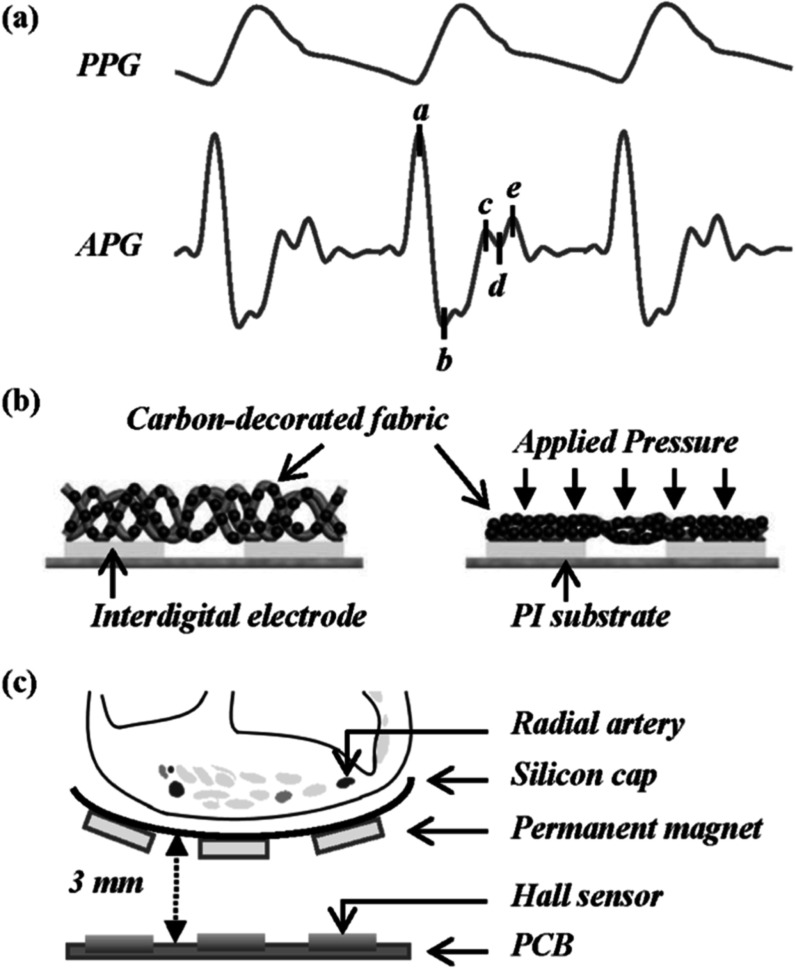
Typical processing technique and non-optical sensors used in alternatives of photoplethysmography (PPG). (a) The calculation of the second derivation of PPG to derive the acceleration plethysmography or acceleration PPG (Takazawa *et al* 1998). (b) A piezoresistive sensor for obtaining the impedance PPG (modified from Luo *et al* (2016)). (c) A series of Hall sensors for capturing the magnetic induction plethysmography (modified from Kim *et al* (2018)).

Targeting the limitations of PPG, other complementary alternatives have been studied either independently, or together with PPG in order to better understand the human vasculature (see figure 32). Different methods including processing techniques and various sensor types have been proposed, and the interested reader is referred to Meng *et al* (2022) for further details of several sensing techniques.

#### Acceleration plethysmography

Acceleration plethysmography (APG) or acceleration PPG uses the second derivative of the waveform of a digital PPG to stabilize the baseline and to separate components of the waveform more easily (Takazawa *et al* 1998). Five waves can normally be found in APG. They are called a, b, c, d, and e waves, respectively (see figure 32).

#### Triboelectric sensing

A triboelectric sensor also generates an electric potential when pressure is applied to it, with the particular advantage of producing a high signal-to-noise ratio. The plethysmogram obtained is similar to the second derivative of the photoplethysmogram (Ouyang *et al* 2017).

#### Piezoresistive sensing

The resistance of a piezoresistive sensor changes when either pressure is applied to it, or the sensor is strained (Meng *et al* 2022). This allows a pulse wave signal to be obtained, as studied for digitizing the radial pulse to estimate cuff-less blood pressure (Luo *et al* 2016). Compared to photoplethysmography, piezoresistive sensors have the advantage of having lower power requirements.

#### Piezoelectric sensing

A piezoelectric sensor generates an electric potential when pressure is applied to it (Meng *et al* 2022). The plethysmogram obtained by a piezoelectric sensor has been found to be similar to the first derivative of the photoplethysmogram (Qananwah *et al* 2020).

#### Capacitive sensing

The capacitance of a capacitive sensor changes when pressure is applied to it (Meng *et al* 2022). The use of a capacitance sensor to measure the pulse wave at the radial artery was demonstrated in Schwartz *et al* (2013).

#### Magnetoelastic sensing

The magnetism of a magnetoelastic sensor changes when pressure is applied to it (Meng *et al* 2022). Recently, textile magnetoelastic sensors have been developed for pulse wave monitoring which are waterproof and suitable for use in sweaty conditions (Zhao *et al* 2021).

#### Magnetic induction plethysmography

The use of a magnetic field-sensing semiconductor Hall sensor is another alternative to PPG (Kim *et al* 2018). By placing a series of permanent magnets of 200 G together with some magnetoresistance sensors in the close vicinity of a peripheral artery, e.g. the radial artery, the pulse waveform can be reconstructed. The systolic and diastolic blood pressure can then be estimated from the signals.

#### Doppler ultrasound and acoustic sensing

Doppler ultrasound has long been used for detecting blood flow. More recently, a wireless carotid neckband Doppler system with wearable ultrasound sensors has been demonstrated to be capable of continuously monitoring the carotid flow velocity pulse wave and displaying the blood flow dynamics and the peak systolic velocity on an external smartphone (Song *et al* 2019). The system was designed with two 2.5 MHz ultrasonic sensors and quantized the acquired Doppler signals by 14-bit analog-to-digital-converters at 40 MHz. Moreover, other acoustic sensors, which can be used for measuring chest sounds, detecting cardiac output, and diagnosing heart problems, have been designed as wearables for sensing pulse waves at the radial artery (Sharma *et al* 2019, Sharma and Rodriguez-Villegas 2021).

#### Impedance plethysmography

Impedance plethysmography detects changes in the conductivity of a region of the body caused by changes in the volumes of tissues. It is already used for respiratory monitoring, and has recently been used to monitor the pulse wave at the upper leg (Haapala *et al* 2021).

#### Speckle plethysmography

Speckle plethysmography is a form of laser imaging which measures the speed of moving light-scattering particles (Ghijsen *et al* 2018). It can be used to obtain a pulse wave, which originates from the scattering of moving red blood cells (Ghijsen *et al* 2018).

### Concluding remarks

PPG is becoming a mature technology commonly implemented in wearable sensing devices. Nevertheless, various alternatives to PPG are emerging to better understand vascular biology and to meet the needs of wider usage in free living and ambient light varying conditions. These complementary methods can be used to study the vasculature and the effects of diseases on the vasculature. Although the measurement of PPG and its alternatives are often made at the periphery, signals collected from them often carry information that is related to the central aorta of a human body. Future works that test PPG and their alternatives in controlled patient groups will be valuable to understand these techniques and their potential applications during the progression of various diseases.

## Acknowledgments

PHC acknowledges funding from the British Heart Foundation (FS/20/20/34626).

## Conflict of interest

The authors Jing Liu and Ni Zhao have a non-provisional patent (US10542894B2) for the multi-wavelength photoplethysmography-based techniques for cardiovascular monitoring.

## Data availability statement

No new data were created or analysed in this study.
